# Sexual dimorphism in cancer: insights from transcriptional signatures in kidney tissue and renal cell carcinoma

**DOI:** 10.1093/hmg/ddab031

**Published:** 2021-02-02

**Authors:** Ruhina S Laskar, Peng Li, Szilvia Ecsedi, Behnoush Abedi-Ardekani, Geoffroy Durand, Nivonirina Robinot, Jean-Noël Hubert, Vladimir Janout, David Zaridze, Anush Mukeria, Dana Mates, Ivana Holcatova, Lenka Foretova, Beata Swiatkowska, Zoran Dzamic, Sasa Milosavljevic, Robert Olaso, Anne Boland, Jean-François Deleuze, David C Muller, James D McKay, Paul Brennan, Florence Le Calvez-Kelm, Ghislaine Scelo, Estelle Chanudet

**Affiliations:** Section of Genetics, International Agency for Research on Cancer (IARC-WHO), 69372 Lyon, France; Laboratory of Population Health, Max Planck Institute for Demographic Research, 18057 Rostock, Germany; Section of Genetics, International Agency for Research on Cancer (IARC-WHO), 69372 Lyon, France; Section of Genetics, International Agency for Research on Cancer (IARC-WHO), 69372 Lyon, France; Section of Genetics, International Agency for Research on Cancer (IARC-WHO), 69372 Lyon, France; Section of Genetics, International Agency for Research on Cancer (IARC-WHO), 69372 Lyon, France; Section of Genetics, International Agency for Research on Cancer (IARC-WHO), 69372 Lyon, France; Science and Research Center, Faculty of Health Sciences, Palacky University, 77900 Olomouc, Czech Republic; Department of Epidemiology and Prevention, Russian N.N. Blokhin Cancer Research Centre, 115478 Moscow, Russian Federation; Department of Epidemiology and Prevention, Russian N.N. Blokhin Cancer Research Centre, 115478 Moscow, Russian Federation; Department of Environmental Health, National Institute of Public Health, 050463 Bucharest, Romania; Department of Public Health and Preventive Medicine, Charles University, Second Faculty of Medicine, 15006 Prague, Czech Republic; Department of Cancer Epidemiology and Genetics, Masaryk Memorial Cancer Institute, 60200 Brno, Czech Republic; Department of Environmental Epidemiology, Nofer Institute of Occupational Medicine, 91-348 Lodz, Poland; Clinic of Urology, Clinical Center of Serbia (KCS), University of Belgrade - Faculty of Medicine, 11000 Belgrade, Serbia; International Organisation for Cancer Prevention and Research, 11070 Belgrade, Serbia; Université Paris-Saclay, CEA, Centre National de Recherche en Génomique Humaine, 91057, Evry, France; Université Paris-Saclay, CEA, Centre National de Recherche en Génomique Humaine, 91057, Evry, France; Université Paris-Saclay, CEA, Centre National de Recherche en Génomique Humaine, 91057, Evry, France; Faculty of Medicine, School of Public Health, Imperial College London, W21NY London, UK; Section of Genetics, International Agency for Research on Cancer (IARC-WHO), 69372 Lyon, France; Section of Genetics, International Agency for Research on Cancer (IARC-WHO), 69372 Lyon, France; Section of Genetics, International Agency for Research on Cancer (IARC-WHO), 69372 Lyon, France; Section of Genetics, International Agency for Research on Cancer (IARC-WHO), 69372 Lyon, France; Unit of Cancer Epidemiology, Department of Medical Sciences, University of Turin, 8-10124 Turin, Italy; Section of Genetics, International Agency for Research on Cancer (IARC-WHO), 69372 Lyon, France

## Abstract

Sexual dimorphism in cancer incidence and outcome is widespread. Understanding the underlying mechanisms is fundamental to improve cancer prevention and clinical management. Sex disparities are particularly striking in kidney cancer: across diverse populations, men consistently show unexplained 2-fold increased incidence and worse prognosis.

We have characterized genome-wide expression and regulatory networks of 609 renal tumors and 256 non-tumor renal tissues. Normal kidney displayed sex-specific transcriptional signatures, including higher expression of X-linked tumor suppressor genes in women. Sex-dependent genotype–phenotype associations unraveled women-specific immune regulation. Sex differences were markedly expanded in tumors, with male-biased expression of key genes implicated in metabolism, non-malignant diseases with male predominance and carcinogenesis, including markers of tumor infiltrating leukocytes. Analysis of sex-dependent RCC progression and survival uncovered prognostic markers involved in immune response and oxygen homeostasis.

In summary, human kidney tissues display remarkable sexual dimorphism at the molecular level. Sex-specific transcriptional signatures further shape renal cancer, with relevance for clinical management.

## Introduction

Sexual dimorphism is common in disease incidences, responses to therapy and outcomes, a fact often neglected in research and clinical practice as in-depth investigations of these differences remain limited (1). The sex of an individual is one of the most stable risk factors for various malignancies, and cancer mortality rates are consistently higher in men (2). We recently conducted a systematic analysis of sex-discrepancy in cancer incidence over time, country and age, highlighting the singularity of kidney cancer (3). Renal cell carcinoma (RCC), the major form of kidney cancer, accounts for 400 000 new cancer cases worldwide. Sex differences are observed in RCC progression, prognosis and outcome, with men typically presenting with larger tumors with higher stage and grade (4). The sex prevalence of established risk factors, i.e. obesity, hypertension and tobacco smoking, has changed dramatically over the past decades. Meanwhile RCC male: female sex ratio has consistently remained 2:1, worldwide and across age groups (3), ruling out variations in exposures and female hormonal protection as the main cause of the observed sex differences.

Mounting evidence suggest differences in molecular features between men and women. The genotype-tissue expression (GTEx) project (5) has identified widespread sex differences in gene expression across several human tissue types (6). Pan cancer analysis from The Cancer Genome Atlas (TCGA) similarly revealed sex differences in global genomic characteristics, including within clinically actionable genes, in various cancers (7,8). Dunford *et al.* highlighted a subset of X-linked tumor suppressor genes that escape X-inactivation in tumors from women, named EXITS genes (9). Bi-allelic expression of EXITS genes in women was recently hypothesized to contribute to reduced cancer rates in women (10). Overall, most studies to date were conducted on tumor tissues and using a multi-cancer design with restricted series of individual cancer types. Normal human kidney tissues are still poorly represented in GTEx (*n* = 85) and TCGA (68 paired tumor/normal tissues). RCC specific studies are rare, mostly confined to mice models or lacking genome-wide representation. The only sex-specific gene expression study targeting exclusively RCC tissues included around 6000 genes (11). Well-powered systematic gene expression studies dedicated to understanding sex difference in normal kidney tissues and RCC tumorigenesis are needed.

Genetic factors are known to influence susceptibility to complex disorders, including cancer (1). Together with a wide range of collaborators worldwide, we jointly conducted kidney cancer genome-wide association studies (GWAS) in search of genetic polymorphisms associated with RCC. The analysis of 10 784 cases and 20 406 controls led to the identification of 13 genome-wide significant risk loci (12). Sex-specific GWAS on the European ancestry subset of 5488 men and 3391 women with RCC provided evidence for sex-specific associations in RCC genetic susceptibility (13). These sex-specific associations included two known RCC risk loci, namely 14q24.2 (transcription regulator *DPF3*) and 2p21 (HIF-like transcription factor *EPAS1*). Genotype-driven variations in gene regulation, reported as expression quantitative trait loci (eQTL), are known to display prominent sex-bias (14). Sex-biased eQTLs contribute to the genetic basis of complex traits, as illustrated in blood (15). Genetic regulation of gene expression has not yet been investigated in human kidney tissues. We hypothesized that this could be of particular relevance in the context of RCC sexual dimorphism.

Here, we have characterized genome-wide expression profiles, co-expression networks and genotype-gene expression associations within large series of RCC tissues and their non-tumor counterparts to elucidate transcriptional differences in kidney tissues of men versus women, and assess potential impact on tumor development and prognosis.

## Results

### Sex-specific characteristics

The characteristics of men and women considered in our discovery series are described in Table 1, including age at diagnosis, geographical origin, tumor stage and status of established RCC risk factors.

**Table 1:** Characteristics of the RCC cases included in the IARC discovery series

	Men (*N* = 362)	Women (*N* = 247)	*P*-value
Mean age at diagnosis (±SD)	60.0 (±10.1)	62.3 (±9.5)	0.005
Country of recruitment, *N* (%)			
Romania	61 (16.9)	27 (10.9)	0.001
Poland	2 (0.6)	4 (1.6)	
Russia	71 (19.6)	74 (30.0)	
Czech Republic	227 (62.7)	137 (55.5)	
Serbia	1 (0.3)	5 (2.0)	
Tumor stage, *N* (%)			
Early stages (I and II)	234 (64.6)	173 (70.0)	0.19
Late stages (III and IV)	128 (35.4)	74 (30.0)	
Tumor grade, *N* (%)			
Grade 1	53 (14.6)	47 (19.0)	0.23
Grade 2	181 (50.0)	125 (50.6)	
Grade 3	70 (19.3)	38 (15.4)	
Grade 4	17 (4.7)	7 (2.8)	
Missing	41 (11.3)	30 (12.2)	
Smoking status, *N* (%)			
Never	140 (38.7)	164 (66.4)	<0.0001
Former	101 (27.9)	28 (11.3)	
Current	107 (29.6)	44 (17.8)	
Missing	14 (3.9)	11 (4.5)	
Mean BMI (±SD)	28.6 (±4.6)	29.2 (±5.3)	0.14
Missing, *N* (%)	19 (5.2)	11 (4.5)	
Hypertension, *N* (%)			
No	175 (48.3)	139 (56.3)	0.04
Yes	174 (48.1)	98 (39.7)	
Missing	13 (3.6)	10 (4.0)	

All cases originated from Eastern Europe, although distribution per country varied for men and women. Overall men were also diagnosed at a younger age, were more likely to be current or former smoker and to have hypertension. Analysis of gene expression variations explained by each covariate revealed that sex was the main driver of variation for most genes, together with age, country of origin as well as stage of disease in tumor tissues, whereas body mass index (BMI), smoking and hypertension explained very little overall variance (<0.01%). Age, country of origin and stage of tumor were adjusted for in all downstream analyses.

### Sex-specific gene expression and gene regulation of normal kidney tissues

#### Gene expression by sex

A total of 58 autosomal genes and 42 genes on sex-chromosome were significantly differentially expressed in women compared to men in non-tumor kidney tissues (Supplementary Material, Table S1). We validated our findings of differential expression according to sex on a subset of 61 samples from the array-based discovery series for which RNA sequencing was available. Of the 100 genes identified in the discovery series, 91 passed quality control within RNA sequencing data. We confirmed significant (*q* < 0.05) differential expression in men compared with women for 59 of 91 (64.8%) genes (Supplementary Material, Table S2). The direction of change was concordant for most of the genes between the two different technical approaches. Further, 45 of 91 genes showed significant sex-difference in expression in an independent series of 60 normal kidney samples from TCGA-KIRC, also with concordant direction of change for the majority of genes (Supplementary Material, Table S3). As expected, the concordance of effect sizes was much stronger between both RNAseq-based datasets (International Agency for Research on Cancer [IARC] validation and TCGA series, *R*^2^ = 0.94) (Supplementary Material, Fig. S2).

**Figure 1:**
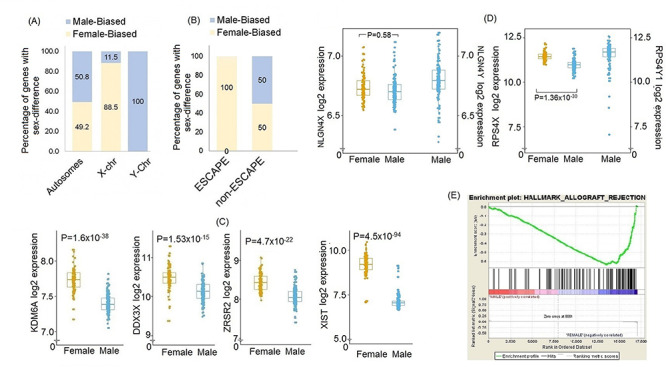
Sex differences in gene expression within normal kidney tissues showing. (**A**) Proportion of male biased (overexpressed in men) and female biased (overexpressed in women) genes among autosomes, and X and Y chromosomes. (**B**) Enrichment of female biased genes that escape from X-inactivation (ESCAPE). (**C**) Expression of different EXITS genes in male and female tissues. (**D**) Example of gene dosage differences for X-linked genes with Y homologues. The *NLGN4* gene shows no statistical difference in expression of its X-linked copy between sexes, whereas the *RPS4* gene fully escapes X-inactivation in women leading to unequal gene dosage compared with men hemizygous for the X-chromosome. Both display high expression of their Y-homolog in men, albeit different degrees of evidence for functional redundancy. (**E**) GSEA functional enrichment suggested enrichment of immune related pathways in women as compared with men with allograft rejection pathway as an example.

Multiple X-chromosome genes showed higher expression in women (Fig. 1A). This reflected the enrichment of genes that escape X-chromosome inactivation in women, termed ESCAPE genes (Fig. 1B). It included two established tumor suppressor escapees, in other words EXIT genes, previously suspected to be associated with excess cancer in men, namely *KDM6A* and *DDX3X*, both showing 25% higher expression in women (FC = 0.8; *q* = 1.7 × 10^−38^ and *q* = 1.5 × 10^−15^, respectively). Additional genes with tumor suppressor activity were also differentially expressed, such as X-inactivation initiator *XIST* and ESCAPE splicing factor *ZRSR2* (Fig. 1C). X-linked genes with Y homologs expressed in kidney and suspected to variably escape X-chromosome inactivation depending on tissue types, including *NLGN4X*, *PRKX* and *TMSB4X,* showed no differences in expression between men and women, whereas lower expression of others, like *RPS4X,* in men, suggested X-inactivation escape, albeit incomplete (FC = 0.72), and coupled to Y-homolog expression (Fig. 1D). Differences were overall more subtle among autosomal genes. Genes overexpressed in men compared with women included tumor specific antigen *CCDC146* (FC = 1.4; *q* = 7.1 × 10^−12^), whereas the expression of *UGT1A6*, known as modulator of hormones and drugs metabolism, was higher in women (FC = 0.7; *q* = 2.9 × 10^−8^).

Looking at gene-disease associations of the genes displaying sexual dimorphism in expression in normal tissues, we identified 59 genes associated with various human diseases in the NIH Genetic Association Database (GAD), of which 47 were associated with a disease having sex difference in incidence rates. Several of these genes were associated with various neurodevelopmental and neurodegenerative disorders, malignancies and renal disorders like nephrotic syndrome, chronic kidney disease, renal hyperuricemia, gout and diseases of reproductive system (Supplementary Material, Table S4). Pathway enrichment analysis of genes differentially expressed between men and women in kidney tissues identified immune related pathways as enriched in women in both IARC and TCGA datasets (Fig. 1E); in contrast, no pathways were enriched in men at false discovery rate (FDR) *q*-value < 0.1 in IARC series (Supplementary Material, Table S5).

#### Morphological characteristics of normal kidney tissues

A centralized pathological review of 173 of the non-tumor tissues qualitatively assessed the presence and severity of chronic renal parenchymal changes (CRPC). A total of 34 non-tumor tissues showed various degrees of CRPC, characterized by a predominance of chronic interstitial nephritis patterns. The presence of CRPC in non-tumor tissues was significantly more frequent in women (21/70; 30%) compared with men (13/103; 13%; *P* = 0.005). One-fourth of the female non-tumor tissues with CRPC (5/21) displayed moderate to severe forms, whereas mild CRPC forms were predominant in men (12/13), suggesting a trend for CRPC to be more extensive in the normal kidney tissues of women (*P* = 0.23).

#### Sex-difference in organization of gene networks

**Figure 2:**
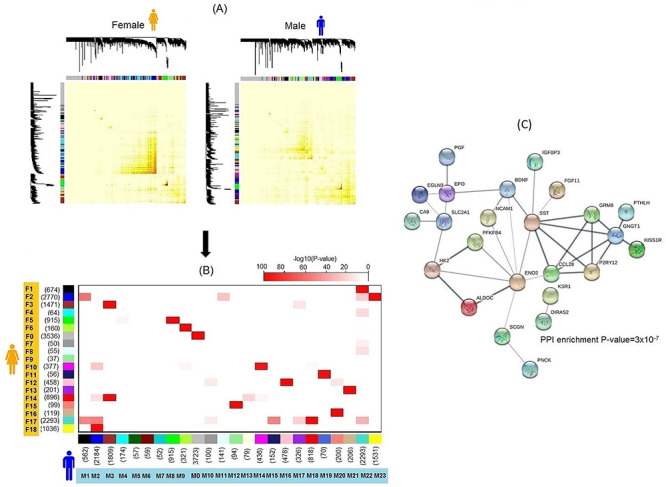
Co-expression networks in male and female normal kidney tissues. (**A**) Functional modules identified in women and men. (**B**) Their concordance in transcript membership represented by high –log 10-*P*-value of concordance. (**C**) Protein–protein interaction networks showing strong enrichment for transcript members of male-specific module M6

To have a better understanding of the transcriptional architecture in normal kidney tissues, we constructed men- and women-specific co-expression networks. Genes were grouped in sets of highly co-regulated genes, named ‘modules’. Overall, 23 modules in men and 18 modules in women were identified (Fig. 2A), mostly overlapping. The shared modules were enriched for similar pathways, depicting the conservation of functional organization in both sexes (Supplementary Material, Fig. S3). Two male modules, M5 and M6, did not have significant overlap with female interaction networks (Fig. 2B) and were mainly composed of transcripts with very little co-expression in women. The M6 module was significantly enriched for genes involved in canonical glycolysis and response to hypoxia and showed strong protein–protein interaction among the module members (Fig. 2C). Differences were also observed in finer network structure for sex chromosomal genes. For example, *XIST* was a member of female co-regulated network but assigned to the set of poorly interacting transcripts in men. Moreover, the y-chromosomal homologs *UTY*, *ZFY* and *DDX3Y* of the identified EXITS genes *KDM6A*, *ZFX* and *DDX3X* respectively did not replace the x-chromosomal homologs in the functional networks and were often part of independent functional networks in men.

#### Sex differences in gene expression regulation

**Figure 3:**
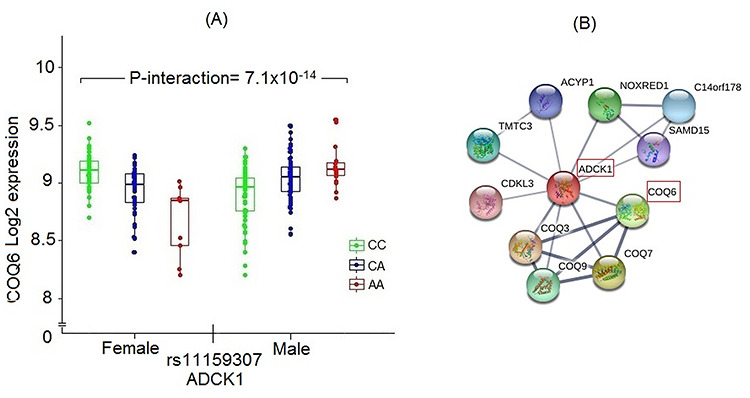
Sex-specific regulation in normal kidney. (**A**) Box plots showing expression of *COQ6* by genotype rs11159307 mapping to *ADCK1* gene having strong sex-specific association. (**B**) Significant protein–protein interaction between proteins coded by the two genes suggesting probable functional significance of the association.

To complement our findings, we assessed sex differences in genetic regulation of gene expression. Although no autosomal or X-chromosomal cis-eQTL with sex-interaction signal passed a Bonferroni correction for multiple testing (4.4 × 10^−13^), we identified 36 trans-eQTLs with significant sex interaction in normal kidney tissues at the same Bonferroni threshold (Supplementary Material, Table S6). Multiple trans-eQTLs had overlapping, upstream or downstream genes that mapped to a regulatory RNA gene, and/or were in regions with DNase hypersensitivity and histone marks in several cell types and cell lines, an indication that the e-QTLs may be within active regulatory regions and promoters or enhancers. Several putative transcription factors binding motifs were also predicted to be altered by the e-QTL variants. The target genes of 13 trans-eQTLs affecting multiple genes were enriched (FDR < 0.05) for at least one transcription factor binding site, indicating co-regulation of the genes. Target genes were strongly enriched for several immune-related pathways including interferon, chemokine and cytokine signaling. Among the single target eQTLs, rs11159307, an intronic variant of *ADCK1* gene showing strong sex interaction (*P*_interaction_ = 7.1 × 10^−14^) in association with *COQ6* expression (Fig. 3A). Coq6 and Adck1 have shared protein–protein interaction networks (Fig. 3B) and are important components of Coenzyme Q biosynthesis that has central role in mitochondrial oxidative phosphorylation. Genetic defects in *COQ6* gene cause renal and neurological manifestations of primary CoQ10 deficiency, like steroid resistant nephrotic syndrome, cerebellar ataxia, seizures and mental retardation (16).

### Sex difference in gene expression and gene regulation of tumor kidney tissues

#### Gene expression by sex in tumors

**Figure 4:**
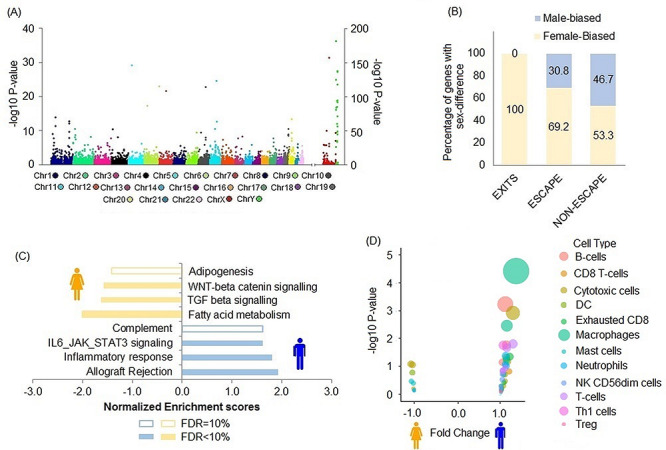
Sex difference in RCC tissues. (**A**) Sex differences in gene expression spread across all chromosomes in tumor tissues as represented by the number of probes with low *P*-value of association with sex. *P*-values represented in *Y* axis are *P*-values of differential expression analysis between men and women. (**B**) Proportion of male and female biased EXITS, ESCAPE and non-ESCAPE X chromosome genes. (**C**) GSEA enrichment scores of immune related genes in male tumors and metabolism related genes in female tumors. Positive enrichment scores depict enrichment of pathways in men and negative depicts enrichment in women. (**D**) Higher expression of gene markers for tumor infiltrating leukocytes in male tumors. Positive FC depicts higher expression in men and negative depicts higher expression in women.

Sex difference in gene expression among tumor tissues was widespread across chromosomes (Fig. 4A), and a total of 1610 autosomal and 103 sex chromosome genes showed sex differences in expression (Supplementary Material, Table S7). We similarly replicated differential expression according to sex in independent cases from the TCGA KIRC series (Supplementary Material, Table S8). Aligned with findings in normal tissues, expression of EXITs genes with important role in RCC was higher in women. This encompassed *DDX3X and KDM6A* with similar fold change (FC) as in normal kidney tissues, as well as *KDM5C* (FC = 0.9). Unlike most genes escaping X-inactivation, a few ESCAPE genes showed higher expression in tumors from men, including the mitochondrial enzyme *MAOA* (FC = 1.8; *q* = 4.5 × 10^−17^), associated with the progression of prostate cancer, as well as, to a lesser extent, G-Protein coupled receptor *GPR143* and the *MSL3* gene involved in dosage compensation pathway in Drosophila (FC = 1.1; *q* = 0.01 and *q* = 0.02, respectively). About half of the genes supposed to follow dosage compensation mechanism by X-inactivation in women actually displayed higher expression in women, suggesting specific ESCAPE mechanisms (Fig. 4B). As in the analysis of normal renal tissue, variable dosage compensation of several non-pseudoautosomal X-linked genes was also observed in tumor tissues.

Top autosomal genes with higher expression in men notably included tumor suppressor gene *RNASET2*, immune related genes like *CCDC146* and *LRRC41*, metabolism-related genes like *ITPR3* and *PTGS1*, heat shock protein *DNAJB13*. Genes with higher expression in women included several mitochondrial energy metabolism genes such as *UQCRH*, *UQCRHL, FABP4* and *HMGCS2*, urate transporter gene *URAT1* (Supplementary Material, Table S7). GSEA analysis identified enrichment of fatty acid metabolism pathway in women and immune related pathways in men (Fig. 4C) that replicated independently in the TCGA series (Supplementary Material, Fig. S3). Multiple established markers of tumor infiltrating leukocytes (17) (Supplementary Material, Table S9) also showed higher expression for men in the IARC series (Fig. 4D) as well as TCGA datasets (Supplementary Material, Fig. S5). Differentially expressed genes in men versus women RCC tissues were enriched in genes associated with metabolic diseases and immune disorders in the GAD catalog, in addition to renal, neurological and chemo-dependence disorders observed in normal kidney tissues (Supplementary Material, Table S10).

#### Morphological characteristics of tumors tissues

Given the observed higher expression of gene markers for tumor infiltrating leukocytes in male tumors, we further explored the morphological characteristics of 341 tumor specimens centrally reviewed. The presence of inflammatory stroma was significantly more frequent in men (23/197; 12%) compared with women (6/138; 4%; *P* = 0.014). There were no significant differences in the extent of inflammation among those 29 cases.

#### Sex-difference in organization of gene networks in tumor tissues

Co-expression network analysis in tumor tissues identified 18 co-regulated modules in women and 19 modules in men. As observed in normal tissues, modules were highly concordant in gene membership between sexes (Supplementary Material, Fig. S6a). Overall, tumor tissues modules were enriched with genes involved in several hallmarks of malignant transformation including angiogenesis, cell-cycle regulation, cell adhesion and extracellular matrix organization (Supplementary Material, Fig. S6b). The main women-specific gene network was enriched for ion-transport and excretory functions, whereas the only men specific module was not enriched for any functional pathways.

#### Sex differences in gene expression regulation in tumors

No cis-eQTL with sex-genotype interaction was identified at the Bonferroni threshold of significance (4.4 × 10^−13^) in 574 GWAS-GE pairs in tumor tissues. In contrast to normal tissues only four trans-eQTLs with significant sex interaction were identified in tumor tissues (Supplementary Material, Table S11). Only one eQTL near regulatory RNAs was associated with multiple gene expression; these genes were not enriched for any transcription factor binding or biological pathways.

#### Role of sex differences in tumor progression and prognosis

**Figure 5:**
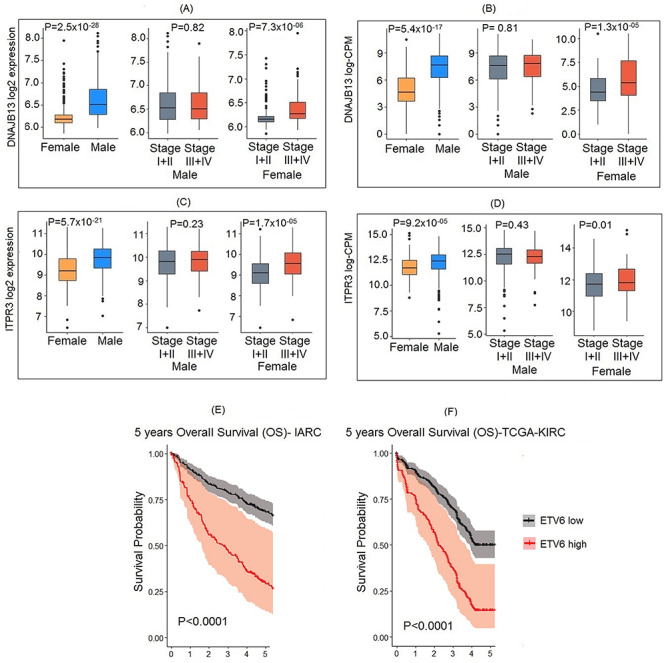
Sex-specific gene expression in tumor progression and prognosis. (**A**, **C**) *DNAJB13* and *ITPR3* having significant sex difference in expression are overexpressed in late stages in female tumors only, showing sex-specific effects in progression. (**B**, **D**) Replication of the findings for *DNAJB13* and *ITPR3* in TCGA-KIRC series. (**E**) Expression of sex-specific gene *ETV6* predicts 5 year overall survival. Low and high are expression values lower and higher than mean respectively. (**F**) Replication of the findings in TCGA-KIRC series.

The presence of sex differences in gene expression signatures in tumor tissues raised the possibility that these genes have a role in progression and prognosis of RCC in a sex dependent manner. Among the 1713 genes with sex differences in expression, 310 genes were differently expressed between early (stages I and II) and late (stages III and IV) stage tumors (Supplementary Material, Table S12). These genes were enriched for various FGFR-signaling and metabolism related pathways in Kyoto Encyclopedia of Genes and Genomes and Reactome gene sets (Supplementary Material, Table S13). Of note, top genes with sex differences in expression within tumor tissues, namely *ITPR3* and *DNAJB13,* show sex-dependent association with stage, which was replicated in TCGA series (Fig. 5A–D).

Expression of 44 of the 1713 genes with sex differences in expression were associated with death rate within 5 years of follow-up in sex stratified analysis adjusting for age and stage after correcting for multiple testing. Association with 5 year OS could be replicated in 21 of them in the TCGA-KIRC dataset, with same direction and mostly similar effect size (Supplementary Material, Table S14). Higher expression levels of *ETV6* oncogene were consistently associated with twice the risk of death within 5 years (IARC series: hazard ratio [HR] 2.24, 95% confidence interval [CI] 1.58–3.19; TCGA series: HR 1.87, 95% CI 1.33–2.66; Fig. 5E and F). A similar association with poor survival was notably observed for high expression levels of signal transductor *BAIAP2L1*, the cancer associated cell surface protein gene *CDCP1*, immune related biomarker *RARRES1*, stress-induced protein gene *TRIB3* and interleukin receptor *IL20RB*, albeit to a lesser extent (HR 1.6–1.1). Conversely, high expression levels of DNA damage checkpoint *CDC14B* were consistently associated with improved overall survival, with over 40% reduction in the hazard of death within 5 years (IARC series: HR 0.27, 95% CI 0.14–0.51; TCGA series: HR 0.60, 95% CI 0.47–0.78). A similar behavior was notably observed for tumor suppressor genes *ID4*, *ANKRD47* and *CLEC3B*, as well as for the circadian clock gene *CRY2, CDCP1* interaction protein gene *FYN*, cell invasion inhibitor *ICAM2* and cell surface tetraspanin *TSPAN7*. Overall, genes associated with survival were enriched for metabolism related pathways (Supplementary Material, Table S15). No association was observed between sex-biased eQTLs and stage or survival either in sex-combined or sex-stratified analysis (Supplementary Material, Table S16).

## Discussion

Studying the differences in molecular architecture between sexes is indispensable for understanding differential susceptibility and managing sexually dimorphic diseases like RCC. In the current study, we take advantage of a unique set of gene expression, genetic and clinical data of RCC patients to provide an outline of the molecular basis of sex differences in normal and tumor kidney tissues and their role in tumor progression and prognosis in RCC.

Among the key findings of the study, is the evidence of sex-specific transcriptional signatures in normal kidney tissues, affecting typical tumor suppressor genes escaping silencing, as well as other genes such as X-inactivation initiator *XIST*, singularly expressed from the inactive X and splicing factor *ZRSR2*, which has been notably associated with excess male risk in leukemia (9)*.* Bi-allelic expression of EXITS genes was hypothesized to provide enhanced cancer protection to women, a phenomenon observed in tumor tissues from several cancer types with male-predominance, including RCC (10,18). Contrary to the previous findings of Y-homologs of EXITS genes like *UTY* replacing *UTX/KDM6A* in male mice brain functional networks (19), we did not detect such replacements of the X-homologs in functional networks of EXITS genes. The hemizygous nature of X-chromosomes resulting in lower expression of potential EXITS genes in normal kidney tissues of men, coupled with variable dosage compensation, may confer increased susceptibility of RCC in men.

The association of sex-different genes with non-malignant diseases having male predominance, such as intellectual disability (20), autism (21), schizophrenia (22), nephrotic syndrome (23), hypertension (24) and hyperuricemia/gout (25), also suggests underlying functional relevance of the genes in the kidney–brain axis. Given that many of these diseases are also associated with RCC (26,27), their underlying role in RCC sex differences might benefit from further exploration.

Sex-specific gene expression was markedly expanded in tumor tissues, with impact on tumor progression and survival. Amplification of sex-differences during tumorigenesis may reflect underlying differences in pathophysiology or tumor microenvironment during tumorigenesis in men and women (8). The unbalanced dosage of X-linked tumor suppressor genes observed here supports the EXITS hypothesis (9). Besides, sex differences in the expression of genes implicated in metabolic disorders, obesity and tobacco related chemo-dependence are suggestive of sex-biased genome-environment interactions that may modulate sexual dimorphism in RCC. Their influence on RCC tumorigenesis should be prioritized in future molecular epidemiology studies. In particular, pathway enrichment analyses of genes with sex-related differential expression are pointing towards sex differences in metabolism, in line with previous findings (8,11). Metabolic shifts to glycolysis and fatty acid synthesis are important for progression and prognosis of RCC (28). Recent evidence suggests sex difference in glycolytic pathways, especially male-specific decreased survival resulting from glycolytic gene overexpression in glioma (29). Our observation of sex-specific association of metabolism-related genes with stage and survival warrants further exploration of sex-specific roles of metabolic shunts in the context of RCC progression and outcome. Detailed clinical studies are notably needed to dissect out the sex-bias of tumor metabolism and their utility in developing new markers and improving metabolic imaging techniques (30).

Our results suggest that the sex-specific immune context observed in non-tumor kidney tissues is relevant for understanding sexual dimorphism in the development of RCC. This is in line with mounting evidence that sex differences in immune responses result in different disease susceptibility in men and women, primarily in autoimmune and infectious diseases (31). In our series, the overexpression of immune or inflammation related genes in tumors from men also indicates the presence of a sex-specific inflammatory environment within the tumor, supported by morphological observations. Inflammatory tumor microenvironment is an important hallmark of cancer and is known to affect proliferation, survival, metastasis and alter response to therapeutic agents (32). Treatment with immune checkpoint inhibitors in patients with advanced disease has emerged as a promising intervention in the management of RCC (33). Tumor antigenicity and inflammation of the tumor microenvironment are necessary to obtain response to immune-checkpoint inhibitors (34). In the context of the improved efficacy of immune checkpoint inhibitors reported in men as compared with women (35), our findings advocate systematic studies exploring sex differences in tumor immunity to gain essential knowledge, which could contribute to optimize clinical management.

Our study unveils prognostic markers with sex-dependent expression, and strong effect size, independently replicated in the TCGA series. This included genes previously suspected to be associated with RCC survival, notably immune related biomarkers, namely *RARRES1* (36,37), interleukin receptor *IL20RB* (38,39), immune activator *CLEC3B* (40,41) and tetraspanin *TSPAN*7 (42,43), as well as several hypoxia-inducible factors target genes such as *CDCP1* (44), *FYN* (45) and *TRIB3* (46), of particular interest given the key role of oxygen and metabolism homeostasis in kidney cancer. Mounting evidence is suggesting the pro-tumorigenic signaling protein CDCP1 as a promising therapeutic target for multiple cancer types, including at the metastatic stage (47). TRIB3 is also being investigated as a cancer biomarker and therapeutic target, especially in the context of its stress adjusting action that links homeostasis, metabolic disease and cancer (48). Among the newly identified sex-dependent RCC prognosis markers in our study are *ETV6*, *ANKRD47* and *CRY2.* Involved in the maintenance of the vascular network, ETV*6* is historically known to be implicated in numerous chromosomal rearrangements in leukemia (49), and was recently established to promote migration and invasion in liver cancer (50). Interestingly, *ANKRD47* promotes cell invasion in hepatocellular carcinoma in an oxygen-dependent manner (51), while the circadian clock gene *CRY2* correlates with overall survival in breast, pancreas, colon and liver cancer (52,53). Given the tight interconnections between the hypoxic response and the circadian pathway in cancer (54), the potential sex-dependant role of hypoxia-inducible factors and their interacting partners would deserve further investigations to elucidate whether sexual dimorphism in such key regulatory mechanisms may further explain sex differences in cancer incidence and outcome.

The main limitation of this study resides in the restricted data available for external replication, in particular for eQTL results. Nevertheless, our study provides a unique resource, consolidating tissue-specific data sets, including in the absence of diseases. Of note, the presence of sex-specific eQTLs in non-tumor kidney tissues echoes previous findings of sex-specific gene regulatory structures in blood (15) and human brain (55). In-silico functional characterization of most of the trans-eQTLs suggested probable regulatory function with target genes enriched for immune related pathways, similar to previous observation in human brain (55). These results are also consistent with our findings of enriched immune related pathways in normal kidney tissues from women as well as the known greater risk of immune related diseases for women (56).

In conclusion, our study of sex-specific transcriptional signatures within large series of normal and tumor kidney tissues demonstrates widespread sex differences in gene expression and regulation in non-tumor kidney tissues. Our findings suggest that EXITS and female-specific immune mechanisms may contribute to sexual dimorphism in RCC incidence. Molecular differences were further extended in tumors, affecting tumor progression and survival in a sex dependent manner. Differential expression of immune and metabolic genes is of particular relevance in the context of sex differences in RCC prognosis and treatment responses.

## Materials and Methods

### Patient materials

A total of 256 paired tumor and non-tumor renal tissues and an additional 353 tumor tissues were collected from 609 cases diagnosed with clear-cell RCC recruited within two IARC case-control studies conducted in Russia, Czech Republic, Romania, Poland and Serbia. The tumors were staged according to International Union Against Cancer TNM classification of malignant tumors 7th Edition. The study protocol was approved by the institutional review boards of IARC and all collaborating centers and hospitals. Written informed consent was obtained from all participating patients.

### Gene expression

IARC discovery series: Total RNA was extracted from tumor and non-tumor tissues using total RNA isolation NucleoSpin RNA II kit (Macherey-Nagel GmbH & Co. KG Düren Germany) following the manufacturer’s instructions. RNA quality was assessed on an Agilent 2100 Bioanalyzer using the Agilent RNA 6000 Nano Kit (Agilent Technologies, Santa Clara, CA, USA) and subjected to gene expression analysis using Illumina HumanHT-12 v4 expression BeadChips (Illumina, Inc., San Diego, USA). Raw signal data were extracted using Ilumina GenomeStudio software (Illumina Inc, San Diego, CA, USA) and samples having signal-to-noise ratios (P95/P05) < 9.5 were excluded. Raw expressions from the samples were pre-processed with variance-stabilizing transformation and quantile normalization using the lumi R package (57) from Bioconductor (Buffalo, NY, USA). The data were log2 transformed and batch corrected using ComBat (Boston University, Boston, MA USA and Broad Institute, Cambridge, MA, USA) (58). Remapping of the Illumina probe-sets to human reference genome hg19 (GRCh37) left with 36 596 uniquely mapped probes. We further excluded probes with detection rate < 10% in tumor and non-tumor samples, leaving a total of 22 063 and 21 844 probes, respectively. Differential analysis of gene expression between men and women was conducted using linear models adjusting for covariates that contributed to explain overall and/or gene-wise variance using principal component analyses (PCA) and variation partition algorithm (59). FC represented the men: women ratio of expression. Probes with *q*-values < 0.05 after multiple testing correction using FDR were considered significant. Data are available on NCBI Gene Expression Omnibus (accession number GSE167093). For technical validation, we used RNAseq raw count data for 61 normal and 98 tumor samples overlapping with the series described previously. To generate the raw counts, FASTAq files from two experiments were mapped using STAR (60), quantified using HTSeq (61) and merged. Raw counts were batch corrected and normalized using the trimmed mean of M-values normalization implemented in edgeR (62). The normalized counts were converted to counts per million and log2 transformed.

TCGA replication: Clinical and level 3 RNA-seq raw counts of 60 normal and 457 RCC tumor tissue samples of the kidney renal clear-cell carcinoma (KIRC) set downloaded from TCGA data portal were analyzed as per the technical validation protocol described previously.

### Co-expression network

The weighted gene co-expression network analysis implemented in the WGCNA R package (63) was used to define co-expression modules in kidney tissues. For multiple probes per gene in expression data sets, probe with the highest expression variance across the samples were retained resulting in including in the analyses 16 780 final probes for non-tumor tissues and 16 954 for tumors. Weighted co-expression analysis started with a similarity matrix constructed by computing the absolute pairwise Pearson correlations between all transcript pairs, followed by converting it into unsigned adjacency matrix by raising it to power parameter ‘β’ (soft-thresholding) calculated by the scale-free topology criterion (63). The adjacency matrix provides a measure of connection strengths between transcript pairs. To understand the modular structures, adjacency matrix was converted to topological overlap matrix (TOM), a measure of both direct and indirect interactions between two genes. Modules of highly co-regulated genes were then identified by grouping genes based on the topological overlap of their connectivity using hierarchical clustering, and dynamic cut-tree algorithm to separate clustering dendrogram branches into gene modules. Each module was assigned a unique color and labelled with a number for ease of comparison. Functional enrichment for each module was obtained using gene ontology and all association with FDR *q*-value < 0.05 was considered significant. Sex-specific networks were constructed independently in men and women, but using the same soft-thresholding β of 7 (Supplementary Material, Fig. S1). Male and female modules were compared by examining the number of genes overlapping in both sexes (64). Fisher’s exact test was used to compute the significance of overlap of genes between the modules of men and women.

### Pathway analysis

Gene set enrichment analysis was performed to investigate whether enrichment of 50 Hallmark gene sets of well-defined biological states or processes differed by sex, using GSEA v3.0 (65). Enrichment analyses of top genes ranked by significance from eQTL, tumor progression and survival analysis were carried out using enrichR (66,67).

### eQTL analysis

A total of 251 and 574 GWAS-GE pairs were used for eQTL analysis in normal and tumor tissues respectively. Genotyping data were a subset from two previously published IARC-Centre National de Genotypage (CNG) GWAS scans and the samples included here were genotyped using HumanHap 317k, 550 or 610Q and Omni5 platforms (12,68). Single nucleotide polymorphisms (SNPs) with call rate < 90%, departure from Hardy Weinberg equilibrium in controls at *P* < 10^−7^ and minor allele frequency < 0.05 were excluded. Imputation of genotypes was done by minimac v3 (69) using the samples of European ancestries from the 1000 Genomes Project (phase 1 release 3) as the reference panel (70). Approximately 6 million SNPs were retained for the final analysis after post imputational QC steps (*r*2 > 0.3). Potential population stratification was assessed using EIGENSTRAT software v5.0.2 (71). For the final analysis we used the QC checked imputed SNPs across the different platforms.

For eQTL analysis, the sex-specific effects were calculated by implementing a model for each probe against SNP, sex and SNP*sex interaction term in the MatrixEQTL R package (72), after adjustment for relevant covariates as per previously defined PCA and variance partition analyses. 1 Mb upstream and downstream range was defined as cis- regulating window. *P*-value of interaction term was used to select significant probe-SNP associations and Bonferroni correction threshold of 4.4 × 10^−13^ was used as significance threshold. All significant signals for a transcript and SNPs at high linkage disequilibrium (LD > 0.5) were considered as one and the eQTL with least *P*-value was reported.

### Histological review of the presence and extent of inflammation in tumor and non-tumor tissues

A total of 341 tumor tissues and 173 non-tumor tissues were centrally reviewed at IARC by one single Pathologist (BAA), blind to the type of tumor as to any characteristics of the cases, including demographic information. In the tumor tissues, the presence of inflammation was reported together with an estimate of the proportion of inflammatory cells. In non-tumor tissues, pathological changes of the renal parenchyma, collectively designed as CRPC, were reported and classified as mild, or moderate to severe, according to a qualitative assessment of the extent of glomerular sclerosis, tubular atrophy, interstitial fibrosis, interstitial inflammation and atherosclerotic changes.

### Sex difference in progression and survival analysis

Genes with differential expression between early and late stages were identified using linear models controlling for age and grade of tumors separately in men and women.

Five-year overall survival status was available for 309 men and 219 women from the discovery series. For the genes with differential expression by sex, using overall survival status, survival time (time to death) and gene expression as continuous variable, sex as strata and adjusted for age, stage and grade of cancer, Cox proportional hazards models were fitted to calculate HR and Wald *P*-values in the IARC data set. To detect sex difference in predicting survival, coxph models with sex-interaction was used. The genes associated with survival in this discovery series was replicated in the TCGA series. Survival analysis was done using the survival R package (73). Visualization of the predicted survival proportion by time for the significant genes from the coxph models were performed using the survminer R package (74). All data analyses and visualization were conducted using R statistical software version 3.5 (75) and ggplot2 (76).

## Supplementary Material

Supplementary_Figures_ddab031

Supplementary_Tables_ddab031
