# Supplementary material for: Sexual dimorphism in cancer: insights from transcriptional signatures in kidney tissue and renal cell carcinoma
Source: Hum Mol Genet. 2021 Feb 2;30(5):343–55. doi: 10.1093/hmg/ddab031 (PMC8098110; doi:10.1093/hmg/ddab031)
Supplement: Supplementary_Figures_ddab031 [file supplementary_figures_ddab031.pdf]

Supplementary Figures

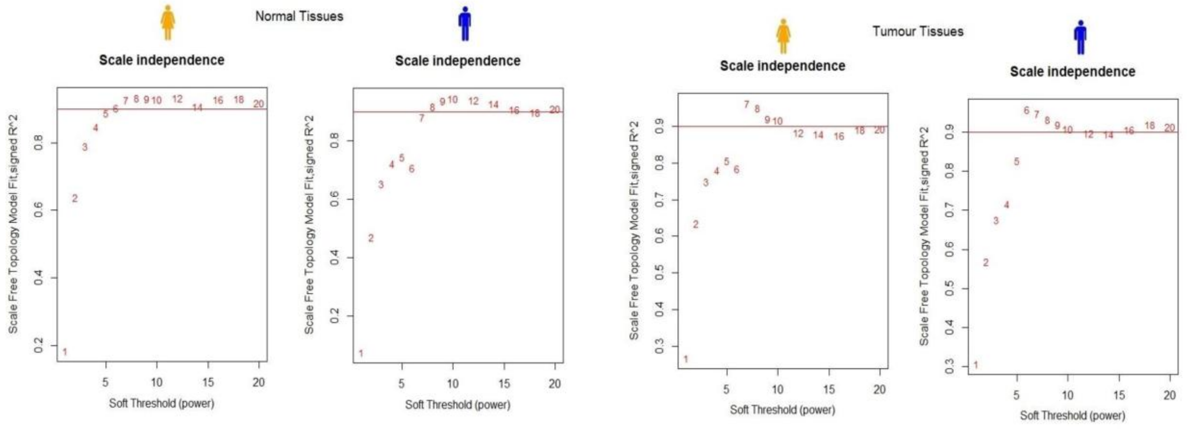

Fig S1: Summary network indices (y-axes) as functions of the soft thresholding power (x-axes) for co-expression network using WGCNA. The approximate scale-free topology fit index curve reached a high value (in this case, roughly 0.90) around the soft-thresholding power of 7 for both sets

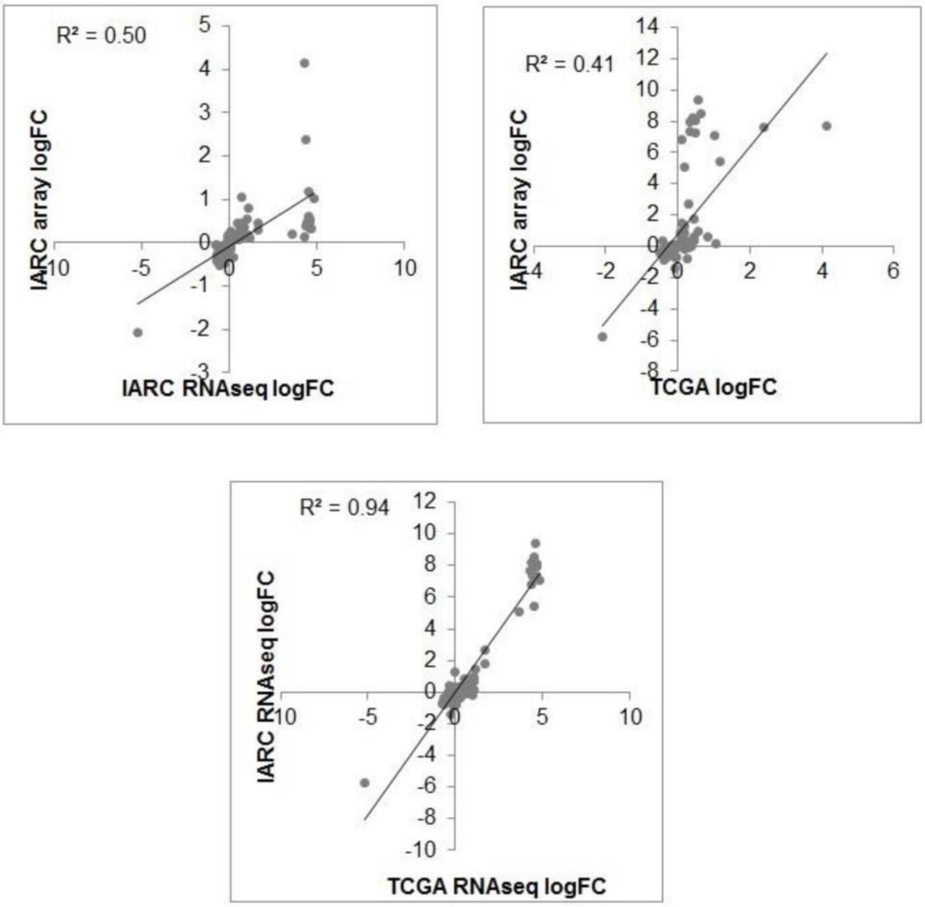

Fig S2: Correlation of effect sizes (logFoldChange) and direction of 90 genes with sex difference in expression among IARC Illumina expression array, IARC RNAseq and TCGA-KIRC RNAseq datasets in normal tissues

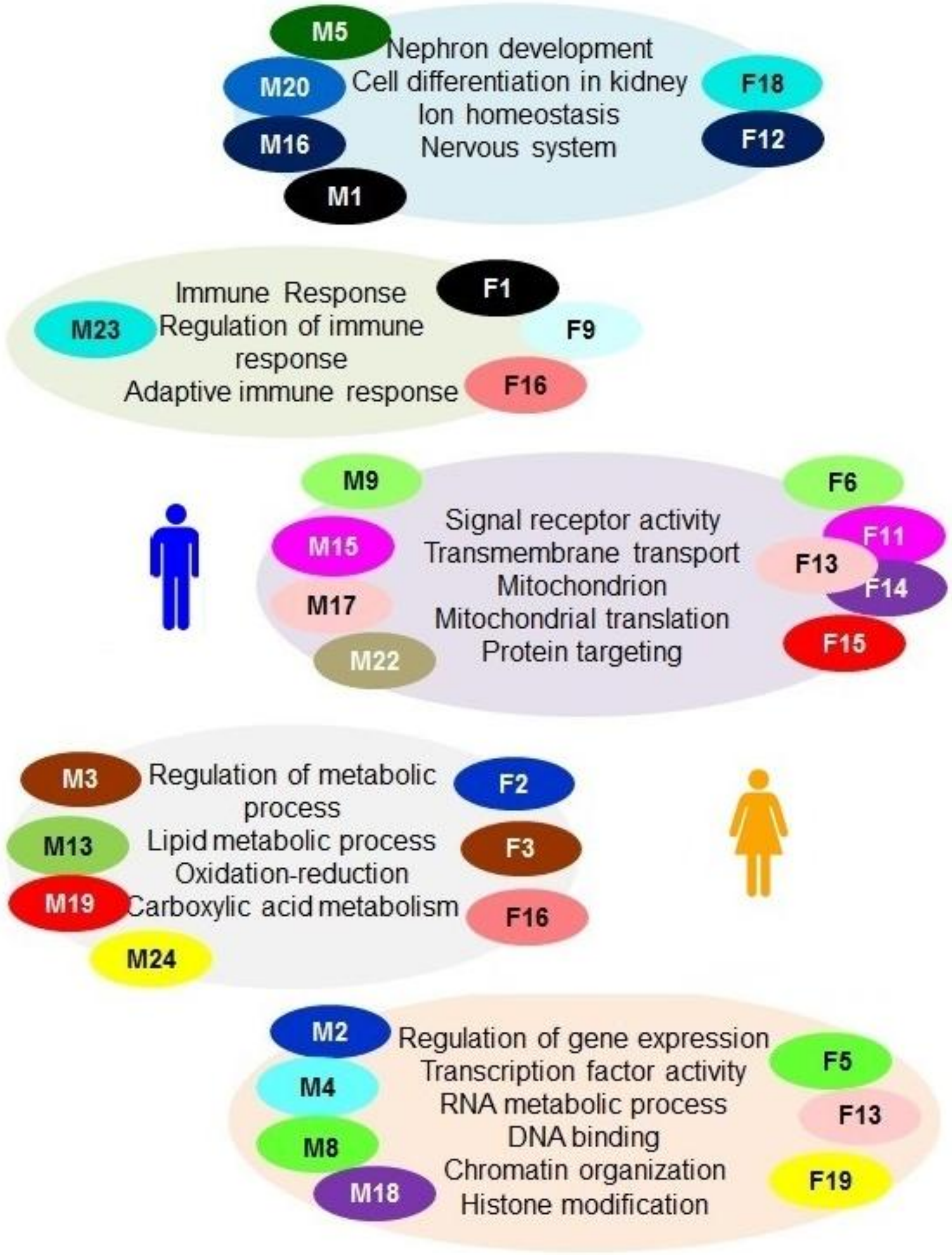

Fig S3 : Functional organization of transcripts in modules shared by sexes in normal kidney tissues are shown. Modules enriched for similar functional processes in both the sexes are grouped together. Modules starting with M stands for male modules and F for female modules. The conserved modules contained genes involved in kidney and nervous system development processes, immune response, cellular signalling and regulation of metabolic and gene expression processes.

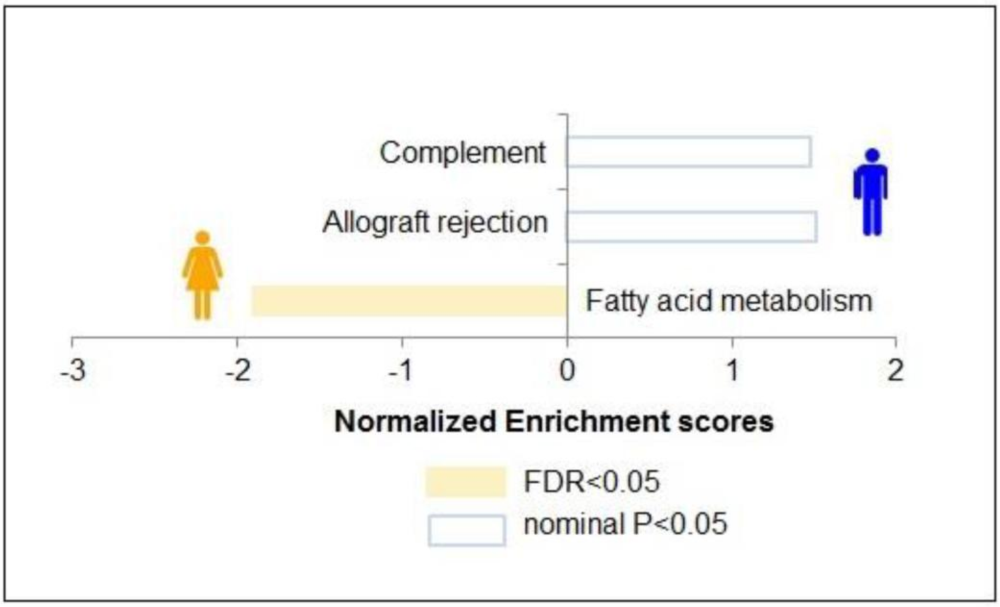

Fig S4: GSEA replication of pathways enriched in males and females in TCGA-KIRC dataset. Positive enrichment scores depicts enrichment of pathways in men and negative depicts enrichment in women.

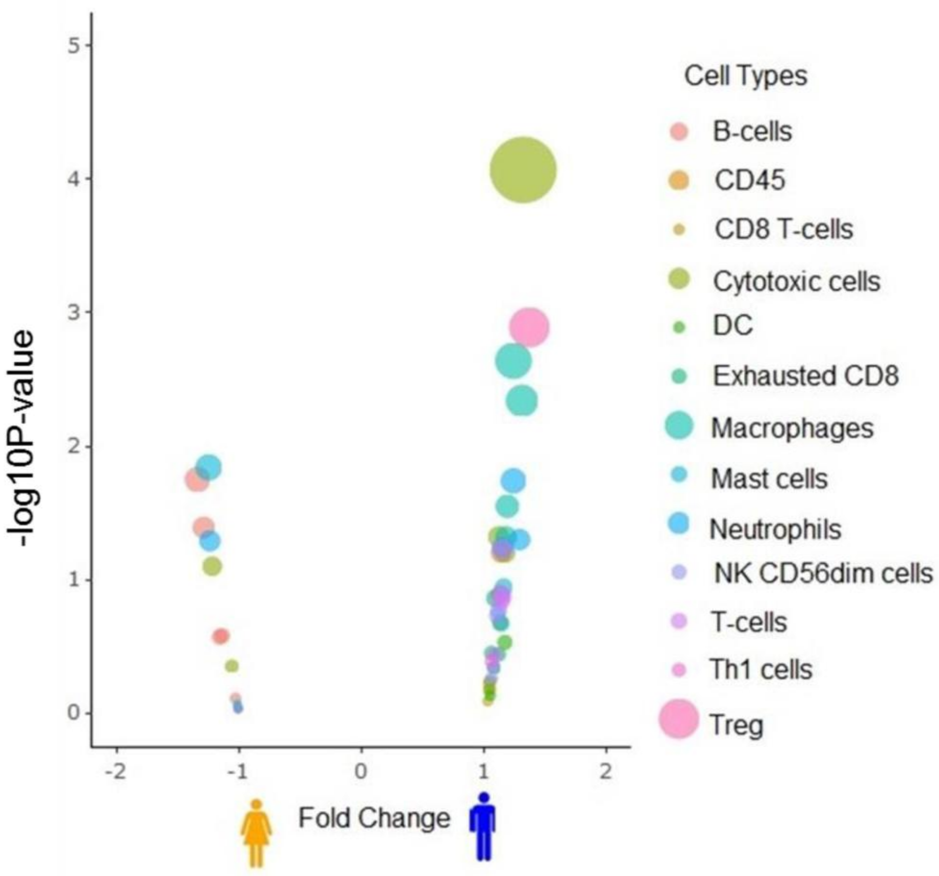

Fig S5: Replication of higher expression of gene markers for tumour infiltrating leukocytes in male tumours in TCGA-KIRC dataset. Positive fold change depicts higher expression in men and negative depicts higher expression in women

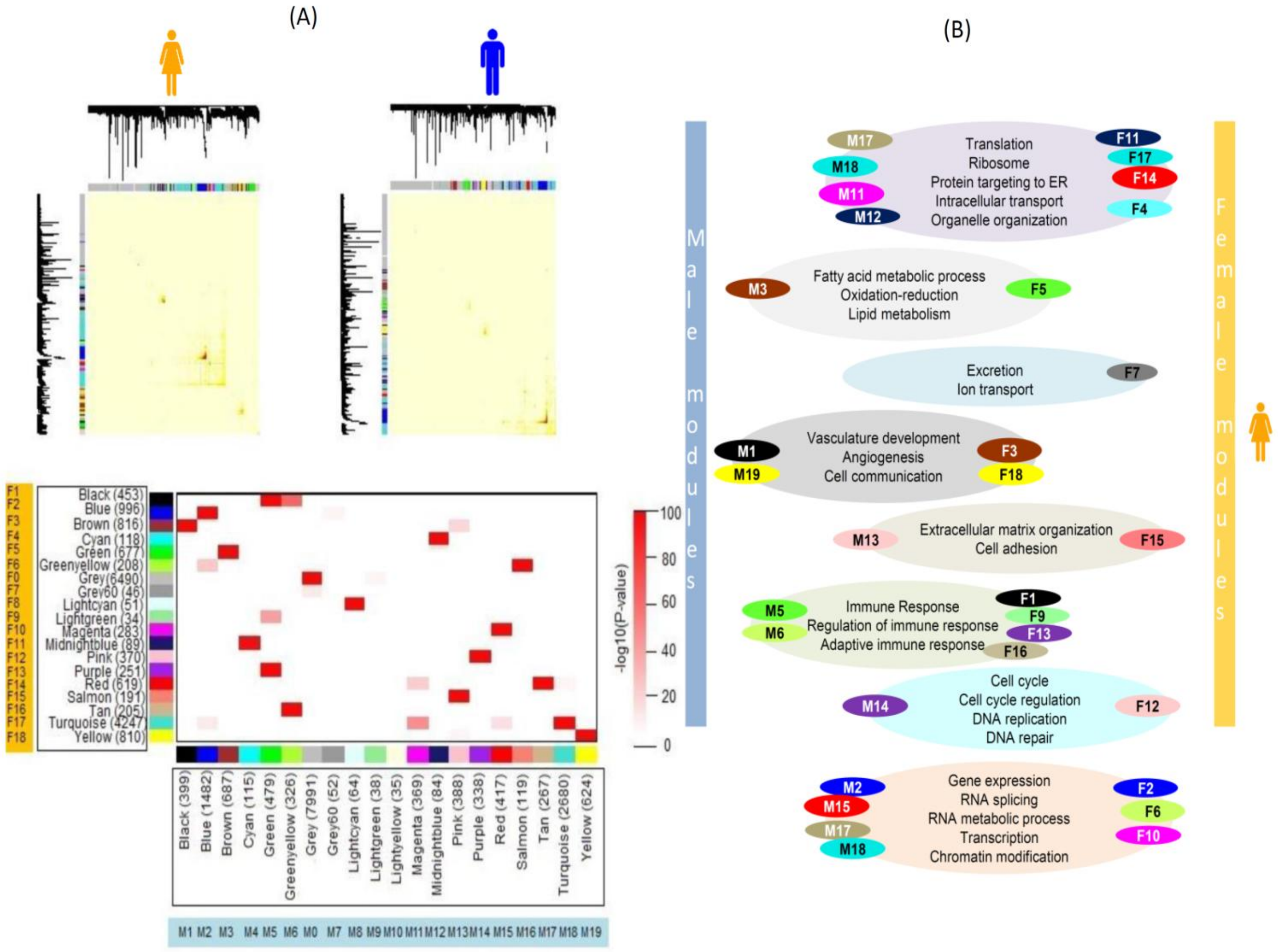

Fig S6: Co-expression networks in male and female Tumour kidney tissues: (A) Functional modules identified in females and males and their concordance in transcript membership represented by high –log10-Pvalue of concordance test (B) Functional organization of transcripts in modules shared by sexes in normal kidney tissues are shown. Modules enriched for similar functional processes in both the sexes are grouped together. Modules starting with M stands for male modules and F for female modules. The conserved modules contained genes involved in kidney and nervous system development processes, immune response, cellular signalling and regulation of metabolic and gene expression processes and also tumour related process like angiogenesis, cell-cycle regulation, cell adhesion and extracellular matrix organization.
