# Supplementary material for: Sexual dimorphism in cancer: insights from transcriptional signatures in kidney tissue and renal cell carcinoma
Source: Hum Mol Genet. 2021 Feb 2;30(5):343–55. doi: 10.1093/hmg/ddab031 (PMC8098110; doi:10.1093/hmg/ddab031)
Supplement: Supplementary_Tables_ddab031 [file supplementary_tables_ddab031.pdf]

Supplementary Table 1

List of genes differentiallly expressed in normal kidney tissues of men and women as per array based expression analyses

Autosomes

Probe	Chromosome	Symbol	Log Fold Change	P Value	Q Value
ILMN_3280952	5	RPS4XP6	-0.235053164	8.16E-33	7.75E-30
ILMN_1698179	3	TAGLN3	0.816486184	2.36E-28	1.98E-25
ILMN_1653286	13	MIR4500HG	0.293703634	1.91E-20	1.19E-17
ILMN_1690209	1	RHEX	0.39135797	6.02E-15	3.06E-12
ILMN_1790555	7	CCDC146	0.4589711	1.42E-14	7.07E-12
ILMN_1795243	13	RPS4XP16	-0.319242044	2.38E-13	1.15E-10
ILMN_1723803	4	SLC2A9	0.167237146	8.67E-13	4.12E-10
ILMN_1758907	2	UGT1A6	-0.437287824	6.64E-11	2.84E-08
ILMN_2344221	21	IGSF5	0.180680782	1.31E-10	5.50E-08
ILMN_1809259	11	HRASLS2	0.4529218	3.65E-10	1.50E-07
ILMN_3199929	11	RPS4XP13	-0.149882765	5.50E-10	2.23E-07
ILMN_1668426	15	SPESP1	0.173411117	1.11E-09	4.41E-07
ILMN_3201216	10	RPS4XP11	-0.107446963	2.43E-09	9.33E-07
ILMN_1745806	17	PEMT	0.208819326	7.04E-09	2.61E-06
ILMN_2211546	20	HAO1	0.123341893	1.34E-08	4.87E-06
ILMN_1737041	10	HABP2	-0.436472573	1.43E-08	5.13E-06
ILMN_1717572	11	PGA5	0.173224498	1.89E-07	6.34E-05
ILMN_3263469	20	RP4-610C12.3	0.082713587	2.08E-07	6.89E-05
ILMN_1772627	4	NSG1	0.360109542	3.40E-07	0.000109198
ILMN_3241554	1	KANK4	-0.169943363	5.83E-07	0.00018452
ILMN_1688318	20	FRG1B	0.196989522	7.48E-07	0.000233445
ILMN_1685125	20	FRG1JP	0.079928733	1.33E-06	0.00039893
ILMN_1810604	11	ELMOD1	-0.142120741	2.70E-06	0.000787304
ILMN_1691790	6	DACT2	-0.168613205	2.94E-06	0.000840903
ILMN_2206474	20	SYNDIG1	-0.157275575	2.96E-06	0.000840903
ILMN_1781060	3	SYN2	0.103944192	4.59E-06	0.001269124
ILMN_1674366	1	LHX4	-0.084716429	8.68E-06	0.002341913
ILMN_1662049	8	AGPAT5	0.168058688	1.27E-05	0.003303749
ILMN_2108735	20	EEF1A2	0.186000729	1.98E-05	0.005033596
ILMN_1689829	5	DNAH5	0.07193262	2.60E-05	0.006522666
ILMN_1793241	5	SRD5A1	0.075417249	2.71E-05	0.006720485
ILMN_1716359	2	SLC19A3	-0.164550129	3.18E-05	0.007707992
ILMN_1736730	6	LRRC16	-0.120651846	4.07E-05	0.009666333
ILMN_1719661	16	SEPX1	0.268526114	5.85E-05	0.013733018
ILMN_1676413	2	VSNL1	-0.110233675	6.63E-05	0.015417357
ILMN_1697701	19	PLEKHJ1	0.090772907	6.76E-05	0.01554438
ILMN_1704537	1	PHGDH	0.272155328	7.55E-05	0.017179157
ILMN_1718929	22	CRYBB3	0.093935282	7.73E-05	0.01741399
ILMN_2174711	21	C21orf129	-0.067411268	9.13E-05	0.02015504
ILMN_1681526	22	SLC5A1	-0.26705712	0.000105	0.022433316
ILMN_1685709	1	TMEM125	-0.144786371	0.000105	0.022433316
ILMN_1767113	2	AOX1	0.442871774	0.000106	0.022532826
ILMN_2154836	21	BTG3	0.122883645	0.000116	0.024064621
ILMN_1754244	17	MYH8	-0.332217294	0.000116	0.024064621
ILMN_1681543	2	RHBDD1	-0.069198102	0.000133	0.027461089
ILMN_2365881	2	ATG16L1	0.096374095	0.000144	0.029420379
ILMN_2105441	4	IGJ	-0.407752977	0.000151	0.030575538
ILMN_3246037	14	ASPG	-0.270132642	0.000159	0.031901585
ILMN_1701052	17	TUBG2	-0.136420745	0.000185	0.036747734
ILMN_1747586	8	TG	-0.045227828	0.000188	0.036922417
ILMN_2360202	5	PPP2R2B	-0.070279046	0.0002	0.038326273
ILMN_3247082	2	FAM150B	0.315787573	0.000206	0.03918762
ILMN_1751886	14	REC8	-0.174881194	0.000212	0.039950758
ILMN_1658333	1	ECM1	0.088509593	0.000246	0.045161795
ILMN_1696657	1	LRRN2	-0.22774655	0.000256	0.046675605
ILMN_1780756	14	RBM23	-0.087209492	0.000264	0.047329773
ILMN_1667578	8	DUSP26	-0.108274094	0.000281	0.049717029
ILMN_1714445	1	SLC6A9	-0.060968651	0.000282	0.049717029

Sex- Chromosomes

Probe	Chromosome	Symbol	Log Fold Chang	P Value	Q Value	Genes described as escaping X-inactivation or having a functional Y-homolog (Dunford et al., 2017)
ILMN_1783142	Y	RPS4Y1	4.14	5.21E-106	1.14E-101	
ILMN_1755537	Y	EIF1AY	2.40	1.03E-96	1.12E-92	
ILMN_1764573	X	XIST	-2.07	4.54E-94	2.48E-90	
ILMN_1670821	Y	TXLNGY	1.19	9.30E-92	4.06E-88	
ILMN_1685690	Y	KDM5D	1.01	3.22E-88	1.17E-84	
ILMN_2191331	Y	RPS4Y2	1.06	1.60E-73	4.36E-70	
ILMN_1739587	Y	UTY	0.63	1.35E-68	2.95E-65	
ILMN_3212921	Y	GYG2P1	0.49	2.10E-63	4.16E-60	
ILMN_2143383	Y	TTTY14	0.56	2.12E-59	3.86E-56	
ILMN_1776195	Y	TMSB4Y	0.49	8.98E-59	1.51E-55	
ILMN_2090059	Y	ZFY	0.40	2.36E-58	3.69E-55	
ILMN_2056795	Y	USP9Y	0.34	1.30E-52	1.78E-49	
ILMN_2210199	Y	NLGN4Y	0.33	3.03E-50	3.89E-47	
ILMN_1654488	X	KDM6A	-0.32	1.39E-41	1.69E-38	ESCAPE gene with functional Y homolog
ILMN_1710136	X	HDHD1A	-0.40	1.18E-39	1.29E-36	ESCAPE gene
ILMN_1673275	X	TRAPPC2	-0.31	8.54E-34	8.48E-31	
ILMN_2166831	X	RPS4X	-0.47	1.36E-30	1.23E-27	ESCAPE gene with functional Y homolog
ILMN_1684873	X	ARSD	-0.51	7.38E-30	6.45E-27	ESCAPE gene
ILMN_2077896	Y	TTTY15	0.13	4.06E-28	3.29E-25	
ILMN_1772163	Y	PRKY	0.19	1.37E-27	1.07E-24	
ILMN_2371700	X	HAUS7	0.56	2.71E-26	2.04E-23	
ILMN_1773868	X	ZRSR2	-0.34	6.45E-25	4.70E-22	ESCAPE gene
ILMN_1704431	X	JPX	-0.28	4.27E-24	3.01E-21	
ILMN_1664348	X	PNPLA4	-0.32	6.56E-23	4.34E-20	ESCAPE gene
ILMN_2364088	X	GEMIN8	-0.17	9.48E-20	5.60E-17	
ILMN_1687484	X	ZFX	-0.20	1.58E-18	9.07E-16	ESCAPE gene with functional Y homolog
ILMN_1794392	X	DDX3X	-0.32	2.73E-18	1.53E-15	ESCAPE gene with functional Y homolog
ILMN_1665717	X	EIF2S3	-0.25	3.56E-16	1.90E-13	ESCAPE gene
ILMN_1701604	X	TBC1D8B	0.17	1.70E-11	7.59E-09	
ILMN_1755419	X	EIF1AX	-0.14	2.34E-11	1.02E-08	ESCAPE gene with functional Y homolog
ILMN_1806313	Y	RBMY1E	0.12	1.81E-09	7.05E-07	
ILMN_1728540	X	FUNDC1	-0.18	1.70E-08	5.99E-06	ESCAPE gene
ILMN_1664001	X	ARSF	-0.36	5.51E-08	1.88E-05	
ILMN_1727462	X	ARSE	-0.28	2.14E-07	6.99E-05	ESCAPE gene
ILMN_1681777	X	SHROOM2	0.10	1.05E-05	0.0028	
ILMN_1754528	Y	DAZ4	0.06	1.74E-05	0.0045	
ILMN_1778956	X	STS	-0.04	3.60E-05	0.0087	ESCAPE gene
ILMN_1772894	X	TMEM27	-0.46	8.14E-05	0.0181	ESCAPE gene
ILMN_2383383	X	PIR	-0.11	9.30E-05	0.0203	ESCAPE gene
ILMN_2319424	X	GYG2	-0.10	0.0002	0.0370	ESCAPE gene
ILMN_1679299	X	IGSF1	-0.05	0.0002	0.0419	
ILMN_1667018	X	ACE2	-0.34	0.0003	0.0471	

Supplementary Table 2

Technical validation using RNA sequencing-based differential expression in normal kidney tissues of men and women

Gene Symbol	hromosom	Log Fold Change	Q Value	Genes described as escaping X-inactivation or having a functional Y-homolog (Dunford et al., 2017)
JPX	X	-0.66	3.70E-14	
GYG2P1	Y	4.64	1.72E-13	
KDM6A	X	-0.70	9.82E-13	ESCAPE gene with functional Y homolog
HDHD1A	X	-0.66	1.86E-11	ESCAPE gene
PRKY	Y	3.64	6.02E-11	
ZFX	X	-0.63	6.42E-11	ESCAPE gene with functional Y homolog
NLGN4Y	Y	4.64	1.83E-10	
TMSB4Y	Y	4.44	2.17E-10	
KDM5D	Y	4.83	2.97E-09	
TTTY14	Y	4.63	3.75E-09	
TTTY15	Y	4.34	8.18E-09	
ZFY	Y	4.38	1.15E-08	
TXLNGY	Y	4.52	1.75E-08	
USP9Y	Y	4.69	2.54E-08	
UTY	Y	4.51	4.29E-08	
TRAPPC2	X	-0.40	5.61E-08	
ARSD	X	-0.55	8.25E-08	ESCAPE gene
EIF1AY	Y	4.36	1.20E-07	
DDX3X	X	-0.46	1.43E-07	ESCAPE gene with functional Y homolog
ZRSR2	X	-0.54	7.68E-07	ESCAPE gene
XIST	X	-5.18	8.17E-07	
PNPLA4	X	-0.55	9.64E-07	ESCAPE gene
STS	X	-0.73	9.89E-07	ESCAPE gene
RPS4Y1	Y	4.29	1.41E-06	
MIR4500HG	13	1.68	2.35E-05	
SLC2A9	4	1.11	3.08E-05	
TBC1D8B	X	0.56	5.67E-05	
RPS4X	X	-0.49	9.14E-05	ESCAPE gene with functional Y homolog
HRASLS2	11	1.69	0.0001	
EIF1AX	X	-0.46	0.0001	ESCAPE gene with functional Y homolog
IGSF5	21	0.94	0.0002	
SPESP1	15	1.05	0.0003	
TAGLN3	3	1.11	0.0005	
GEMIN8	X	-0.24	0.0006	
PEMT	17	0.65	0.0006	
HAUS7	X	1.01	0.0012	
CCDC146	7	0.46	0.0016	
PHGDH	1	0.69	0.0019	
NSG1	4	0.86	0.0020	
IGSF1	X	-0.72	0.0034	
PPP2R2B	5	-0.44	0.0040	
EIF2S3	X	-0.28	0.0044	ESCAPE gene
SLC19A3	2	-0.49	0.0047	
HAO1	20	1.19	0.0080	
SEPX1	16	0.74	0.0088	
AGPAT5	8	0.30	0.0126	
ELMOD1	11	-0.54	0.0133	
PLEKHJ1	19	0.41	0.0137	
CRYBB3	22	0.74	0.0169	
AOX1	2	0.70	0.0251	
FUNDC1	X	-0.25	0.0257	ESCAPE gene
FRG1B	20	0.25	0.0270	
LRRC16	6	-0.16	0.0275	
EEF1A2	20	1.01	0.0337	
SHROOM2	X	0.24	0.0359	
FAM150B	2	0.62	0.0376	
LRRN2	1	-0.45	0.0389	
DNAH5	5	0.54	0.0408	
SYN2	3	0.55	0.0437	
RHBDD1	2	-0.12	0.0575	
UGT1A6	2	-0.42	0.0578	
HABP2	10	-0.66	0.0745	
LHX4	1	-0.28	0.1166	
PIR	X	-0.18	0.1324	ESCAPE gene
ARSE	X	-0.31	0.1535	ESCAPE gene
SLC6A9	1	-0.31	0.2421	
SYNDIG1	20	-0.18	0.2473	
KANK4	1	-0.25	0.2497	
BTG3	21	0.16	0.2616	
RBM23	14	0.07	0.3260	
TG	8	-0.21	0.3468	
MYH8	17	0.28	0.4088	
TMEM125	1	0.10	0.4140	
IGJ	4	-0.34	0.4789	
ATG16L1	2	-0.08	0.5020	
DUSP26	8	0.13	0.5071	
GYG2	X	-0.09	0.5227	ESCAPE gene
TMEM27	X	-0.19	0.5344	ESCAPE gene
ACE2	X	-0.19	0.5482	
REC8	14	-0.12	0.5766	
RPS4XP16	13	0.07	0.5828	
ECM1	1	0.14	0.6173	
VSNL1	2	0.12	0.6834	
PGA5	10	-0.05	0.7551	
ARSF	X	-0.10	0.7556	
SGK494	17	0.07	0.7721	
TUBG2	17	0.04	0.8234	
SLC5A1	22	-0.06	0.8515	
DACT2	6	0.03	0.8886	
SRD5A1	5	0.01	0.9042	
ASPG	14	0.03	0.9244	

Supplementary Table 3

Independent replication of differential expression in normal tissues of men and women using the TCGA-KIRC series

Gene Symbol	Chromosome	Log Fold Change	Q Value	Genes described as escaping X-inactivation or having a functional Y-homolog (Dunford et al., 2017)
TTTY14	Y	9.38	9.39E-25	
GYG2P1	Y	8.09	4.72E-22	
ZFY	Y	8.22	9.45E-22	
UTY	Y	8.52	1.28E-21	
TMSB4Y	Y	7.30	9.89E-21	
EIF1AY	Y	7.65	8.56E-18	
USP9Y	Y	7.97	3.16E-17	
NLGN4Y	Y	7.32	3.43E-17	
KDM5D	Y	7.12	5.52E-16	
TTTY15	Y	6.81	6.48E-16	
PRKY	Y	5.10	1.56E-15	
RPS4Y1	Y	7.72	1.75E-14	
HDHD1A	X	-0.78	7.75E-13	ESCAPE gene
JPX	X	-0.62	1.48E-12	
KDM6A	X	-0.67	1.60E-12	ESCAPE gene with functional Y homolog
TRAPPC2	X	-0.54	3.48E-11	
EIF1AX	X	-0.46	3.61E-11	ESCAPE gene with functional Y homolog
ZRSR2	X	-0.51	5.98E-11	ESCAPE gene
TXLNGY	Y	5.42	8.42E-11	
XIST	X	-5.76	1.81E-09	
ZFX	X	-0.54	2.30E-09	ESCAPE gene with functional Y homolog
DDX3X	X	-0.50	1.05E-07	ESCAPE gene with functional Y homolog
PNPLA4	X	-0.59	5.34E-07	ESCAPE gene
RPS4X	X	-0.45	6.42E-07	ESCAPE gene with functional Y homolog
STS	X	-0.74	1.86E-06	ESCAPE gene
EIF2S3	X	-0.30	7.87E-06	ESCAPE gene
LHX4	1	-1.41	2.91E-05	
GEMIN8	X	-0.29	9.64E-05	
MIR4500HG	13	2.69	0.0001	
ARSD	X	-0.42	0.0001	ESCAPE gene
GYG2	X	-0.72	0.0002	ESCAPE gene
HAUS7	X	0.97	0.0008	
AGPAT5	8	0.36	0.0009	
HRASLS2	11	1.79	0.0022	
FRG1B	20	0.34	0.0027	
PGA5	10	1.30	0.0061	
DNAH5	5	0.89	0.0108	
IGSF1	X	-0.68	0.0128	
SRD5A1	5	0.30	0.0151	
SGK494	17	-0.79	0.0184	
SLC2A9	4	0.91	0.0253	
PEMT	17	0.37	0.0268	
TBC1D8B	X	0.43	0.0271	
TAGLN3	3	0.64	0.0325	
CCDC146	7	0.39	0.0360	
FAM150B	2	-0.09	0.0577	
HAO1	20	1.49	0.0613	
PLEKHJ1	19	0.18	0.0775	
FUNDC1	X	-0.15	0.0920	ESCAPE gene
RBM23	14	0.07	0.1028	
SLC19A3	2	-0.39	0.1077	
ATG16L1	2	0.19	0.1264	
ELMOD1	11	-0.36	0.1344	
IGSF5	21	0.31	0.1438	
ARSF	X	-0.84	0.1593	
ARSE	X	-0.41	0.1671	ESCAPE gene
RHBDD1	2	0.07	0.1752	
BTG3	21	0.16	0.1854	
TMEM27	X	-0.47	0.1967	ESCAPE gene
VSNL1	2	-0.38	0.2100	
AOX1	2	0.62	0.2176	
PHGDH	1	0.19	0.2610	
PPP2R2B	5	-0.21	0.2684	
DUSP26	8	-0.29	0.2691	
SLC6A9	1	-0.18	0.2789	
SPESP1	15	0.20	0.2961	
MYH8	17	-0.36	0.3035	
PIR	X	-0.09	0.3697	ESCAPE gene
SYN2	3	0.31	0.4261	
KANK4	1	-0.22	0.4565	
ASPG	14	-0.41	0.4593	
RPS4XP16	13	-0.13	0.4719	
UGT1A6	2	-0.23	0.4780	
HABP2	10	-0.33	0.4883	
SEPX1	16	0.21	0.5005	
CRYBB3	22	0.36	0.5009	
FRG1JP		-0.19	0.5017	
TUBG2	17	-0.07	0.5563	
IGJ	4	0.39	0.5702	
ACE2	X	-0.29	0.5830	
REC8	14	-0.08	0.6431	
EEF1A2	20	-0.21	0.6436	
SLC5A1	22	-0.17	0.7223	
TG	8	-0.11	0.7433	
TMEM125	1	0.05	0.7766	
SHROOM2	X	0.03	0.8087	
SYNDIG1	20	-0.04	0.8399	
LRRC16	6	-0.01	0.8615	
LRRN2	1	-0.02	0.9383	
DACT2	6	-0.01	0.9555	
ECM1	1	0.00	0.9991	

Supplementary Table 4

Gene-Disease association of genes differentially expressed in normal kidney tissues of men and women

Gene Name		Association Type	Disease Name	Sex difference	PMID
Angiotensin I converting enzyme 2	ACE2	Genetic Variation	Hypertensive disease, Diabetes Mellitus, Diabetic Nephropathy	Yes	16866021, 18022600, 19286756, 16215952, 20160196, 19684612, 17473847, 16211375
		Altered Expression	Cardiovascular Diseases, Diabetes Mellitus, Hypertensive disease, Diabetic Nephropathy, Kidney Diseases	Yes	24564768, 22811473, 23823602, 15538735, 19077419
Arylsulfatase E	ARSE	GeneticVariation	Brachytelephalangic Chondrodysplasia Punctata	Yes	12567415, 9409863
Autophagy related 16 like 1	ATG16L1	Genetic Variation	Inflammatory Bowel Diseases: Crohn Disease, Ulcerative Colitis	Yes	17068223, 18499543, 18671817, 18587394, 19276991
		Altered Expression	Crohn Disease		24036151
ylglycerol-3-phosphate O-acyltransfera	AGPAT5	Genetic Variation	Tobacco Use Disorder	Yes	20379614
Aldehyde oxidase 1	AOX1	Altered Expression	Xanthinuria, Type I	NA	14551354
arylsulfatase D	ARSD	Genetic Variation	Neurodevelopmental Disorders: Autistic Disorder	Yes	22037497, 21839838
		AlteredExpression	Autistic Disorder		26731442
arylsulfatase F	ARSF	AlteredExpression	Non-Small Cell Lung Carcinoma, Renal carcinoma	Yes	20682707, 19534619
asparaginase	ASPG	Genetic Variation	Acute lymphocytic leukemia, Allergic disposition	Yes	12518376, 25987655
BTG anti-proliferation factor 3	BTG3	AlteredExpression	Ovarian carcinoma, stomach carcinoma, lung carcinoma, renal carcinoma	Yes	25238703, 23657964, 23419616, 19221000
coiled-coil domain containing 146	CCDC146	Genetic Variation	Tobacco Use Disorder	Yes	20379614
crystallin beta B3	CRYBB3	Genetic Variation	Cataract	Yes	23508780
evelled binding antagonist of beta caten	DACT2	Genetic Variation	Tobacco Use Disorder	Yes	20379614
		Altered Expression	Colorectal neoplasms, lung carcinoma	Yes	18370954, 22806826
deleted in azoospermia 4	DAZ4	Genetic Variation	Oligospermia	NA	16963411
DEAD-box helicase 3, X-linked	DDX3X	Genetic Variation	Medulloblastoma, Intellectual Disability	Yes	25043297, 26235985
		Altered Expression	Breast, liver, endometrial carcinoma	Yes	23696831, 16818630, 22492871
dynein axonemal heavy chain 5	DNAH5	Genetic Variation	Ciliary Motility Disorders	No	24150548
dual specificity phosphatase 26	DUSP26	Altered Expression	Glioma	Yes	19043453
extracellular matrix protein 1	ECM1	Genetic Variation	Inflammatory Bowel Diseases: Crohn Disease, Ulcerative Colitis	Yes	19817673, 19861958, 18438406
ryotic translation initiation factor 1A, X-l	EIF1AX	Genetic Variation	Uveal melanoma	Yes	23793026
	EIF1AY	Genetic Variation	Autistic Disorder	Yes	19605777
tic translation initiation factor 2 subunit	EIF2S3	Genetic Variation	nervous system disorder	Yes	23063529
ELMO domain containing 1	ELMOD1	Genetic Variation	Narcolepsy, Tobacco Use Disorder	Yes	20677014, 20379614
	GYG2	Genetic Variation	Leigh Disease	NA	24100632
hyaluronan binding protein 2	HABP2	Genetic Variation	Hepatitis C, Liver Cirrhosis	Yes	19105210
		Altered Expression	Hepatitis, Chronic liver disease		22989567
hydroxyacid oxidase 1	HAO1	Genetic Variation	Hyperoxaluria	NA	12110000, 24996905
		Altered Expression	Primary hyperoxaluria, type I	NA	26758691
immunoglobulin superfamily member 1	IGSF1	Genetic Variation	Central hypothyroidism	Yes	23143598
immunoglobulin superfamily member 5	IGSF5	Genetic Variation	Tobacco Use Disorder	Yes	20379614
KN motif and ankyrin repeat domains 4	KANK4	Genetic Variation	Nephrotic Syndrome	Yes (in children)	25961457
		Altered Expression	Rheumatoid Arthritis	Yes	24397353
LIM homeobox 4	LHX4	Genetic Variation	Pituitary Diseases	NA	18445675, 15998782
		Altered Expression	Pituitary Diseases	NA	25955177
leucine rich repeat neuronal 2	LRRN2	Genetic Variation	Tobacco Use Disorder	Yes	20379614
myosin heavy chain 8	MYH8	Genetic Variation	Congenital joint contractures	NA	15992699
neuroligin 4 X-linked	NLGN4X	Genetic Variation	Autistic Disorder	Yes	12669065
neuroligin 4 Y-linked	NLGN4Y	Genetic Variation	Autistic Disorder	Yes	19605777
sphatidylethanolamine N-methyltransfe	PEMT	Genetic Variation	Metabolic Syndrome X, Fatty Liver, breast carcinoma	Yes	18230680, 20492333
		Altered Expression	Liver neoplasms, obesity	Yes	9989271, 23724137
phosphoglycerate dehydrogenase	PHGDH	Genetic Variation	Neu-Laxova syndrome	NA	24836451
pirin	PIR	Altered Expression	Leukemia, Myelocytic, Acute	Yes	20010624
atin like phospholipase domain containi	PNPLA4	Altered Expression	Obesity	Yes	17609260
ein phosphatase 2 regulatory subunit B	PPP2R2B	Genetic Variation	Neurodegenerative Disorders	Yes	11719278
meiotic recombination protein	REC8	Genetic Variation	Azoospermia	NA	18570052
ribosomal protein S4 Y-linked 1	RPS4Y1	Genetic Variation	Anxiety Disorders	Yes	25558953
shroom family member 2	SHROOM2	Genetic Variation	colorectal cancer	Yes	22634755
solute carrier family 19 member 3	SLC19A3	Genetic Variation	Brain Diseases	Yes	23469184
		Altered expression	Breast carcinoma	Yes	12861052
solute carrier family 2 member 9	SLC2A9	Genetic Variation	Renal hyperuricemia, Gout, Hypertensive disease, chronic kidney disease,	Yes	18327257, 24805955, 25435339, 23043931
solute carrier family 5 member 1	SLC5A1	Genetic Variation	Familial renal glucosuria, Congenital glucose-galactose malabsorption	NA	19965550, 11406349
solute carrier family 6 member 9	SLC6A9	Genetic Variation	Hypertensive disease, Alcoholic Intoxication,	Yes	19556729, 19650813
steroid 5 alpha-reductase 1	SRD5A1	Genetic Variation	Polycystic Ovary Syndrome, Prostate neoplams, breast neoplasms	Yes	20200332, 20634197, 20634197
steroid sulfatase	STS	Genetic Variation	Ichthyosis X-Linked	Yes	20236202
synapsin II	SYN2	Genetic Variation	Bipolar disorder, Schizophrenia	Yes	16131404
transgelin 3	TAGLN3	AlteredExpression	Alcoholic Intoxication, Chronic	Yes	15948156
thyroglobulin	TG	Genetic Variation	Thyroid Diseases and carcinoma, congenital hypothyroidism, Autoimmune Diseases	Yes	18514160, 21757724
collectrin, amino acid transport regulato	CLTRN	Genetic Variation	Hartnup Disease	NA	18424768
		AlteredExpression	Conventional (Clear Cell) Renal Cell Carcinoma	Yes	27417314
thymosin beta 4 Y-linked	TMSB4Y	Genetic Variation	Carcinoma of Male Breast	Yes	26702755
trafficking protein particle complex 2	TRAPPC2	Genetic Variation	Disorder of skeletal system	Yes	12361953
tubulin gamma 2	TUBG2	Genetic Variation	Breast Carcinoma	Yes	20508983
glucuronosyltransferase family 1 memb	UGT1A6	Genetic Variation	Breast, Colorectal carcinoma	Yes	20686835, 17085674
ubiquitin specific peptidase 9 Y-linked	USP9Y	Genetic Variation	Male infertility	NA	18062861
anscribed tetratricopeptide repeat cont	UTY	Genetic Variation	Coronary heart disease	Yes	18511697
visinin like 1	VSNL1	Genetic Variation	Cognition Disorders	Yes	24598588
X inactive specific transcript	XIST	Genetic Variation	Intellectual Disability, Mammary neoplasms	Yes	10982188, 17545591
Zinc Finger Protein X-Linked	ZFX	Genetic Variation	Neurodevelopmental Disorders	Yes	26740508

Supplementary table 5

GSEA analysis of normal kidney tissues of men and women

	IARC			TCGA		
Pathways in women	Normalized Enrichment Scores	Nominal P Value	Q Value	Normalized Enrichment Scores	Nominal P Value	Q Value
INTERFERON ALPHA RESPONSE	-2.48	0.00	0.00	-1.25	0.10	0.21
ALLOGRAFT REJECTION	-2.39	0.00	0.00	-1.61	0.00	0.01
INTERFERON GAMMA RESPONSE	-2.21	0.00	0.00	-1.53	0.00	0.02
WNT BETA CATENIN SIGNALING	-1.64	0.01	0.08	-0.92	0.63	1.00
FATTY ACID METABOLISM	-1.62	0.00	0.07	-0.97	0.55	0.99

	IARC			TCGA		
Pathways in men	Normalized Enrichment Scores	Nominal P Value	Q Value	Normalized Enrichment Scores	Nominal P Value	Q Value
HEME METABOLISM	1.41	0.07	0.21	-1.19	0.14	0.34
TNFA SIGNALING VIA NFKB	1.41	0.05	0.25	-1.75	0.00	0.00
OXIDATIVE PHOSPHORYLATION	1.33	0.09	0.25	2.32	0.00	0.00
BILE ACID METABOLISM	1.45	0.08	0.28	-0.62	1.00	1.00
HYPOXIA	1.25	0.12	0.31	-0.89	0.74	1.00
ANDROGEN RESPONSE	1.46	0.06	0.36	-0.96	0.54	0.97

Supplementary table 6

eQTLs in normal kidney tissue

SNP	Chromosome	Position	**Overlapped Gene**	**Type**	**Nearest Upstream Gene**	**Type**	**Nearest Downstream Gene**	**Type**	A1	A2	MAF	latory proteins b	Chromatin structure (DNAse sensitive)	chromatin state	TF binding motifs altered by the variant	Previous eQTL hits	Probe	Gene	Gene_chromosome	Interaction P-value	TF enriched	Pathways enriched
rs12728928	1	210763914	HHAT	protein_coding, intronic					C	G	0.05			EHMks (ESDR, BRN)	BRCA, Evi-1,LBP-1, Pax-4, RFX-5, TATA		ILMN_1705892	SH2D1A	X	7.59E-20		
rs67756583	3	22635435			HMGB1P5	pseudogene	AC092421.1	pseudogene	T	C	0.06			EHMs (ESC)	AP-2rep,SP1		ILMN_1698367	CD84	1	2.79E-13	-	Adaptive Immune System_Homo sapiens
																	ILMN_1699160	ITK	5	6.77E-14		
																	ILMN_1658399	KLRG1	12	2.22E-13		
																	ILMN_1665943	MAP4K1	19	1.03E-13		
																	ILMN_1705892	SH2D1A	X	5.52E-17		
																	ILMN_2319000	MATK	19	8.37E-18		
rs139042028	4	110819862			KRT19P3	pseudogene	EGF	protein_coding	A	G	0.05				Hoxa7		ILMN_1745397	OAS3	12	4.39E-14		
rs17487930	4	16492952	RP11-446J8.1	antisense, intronic					A	C	0.06				Foxd3, Maf, Mrg:Hoxa9, Mrg, TAL1	1	ILMN_1712431	FAM113B	12	6.64E-13		
rs189149546	4	189181022			RP11-366H4.1	lincRNA	RP11-219G10.1	pseudogene	C	G	0.05				BDP1, CTCFL, CTCF, E2A, Ets, Lrf, Cmo2-complex, Mxi, Myf, NF-1, NRSF, RXRA, Rad21, SMC3, SPI, TAL1		ILMN_1664543	IFIT3	10	9.82E-13		
rs6844914	4	175564860	GLRA3	3UTR					C	T	0.06				Egr-1		ILMN_1658399	KLRG1	12	1.52E-13	-	-
																	ILMN_2395981	PYHIN1	1	8.02E-13		
rs75344855	4	168794423			RP11-521F1.1	pseudogene	RP11-310I9.1	antisense	C	T	0.06				Gfi1,Hoxc9		ILMN_1690907	CCR6	6	3.20E-13	-	Cytokine-cytokine receptor interaction
																	ILMN_1777519	ITGB7	12	3.25E-15		
																	ILMN_1665943	MAP4K1	19	5.93E-13		
																	ILMN_1705892	SH2D1A	X	2.74E-14		
																	ILMN_1765109	TNFRSF25	1	3.56E-13		
rs76145187	4	168722184			RP11-521F1.1	pseudogene	RP11-310I9.1	antisense	G	A	0.05						ILMN_1690907	CCR6	6	1.51E-14		
rs111860654	5	74583987			ANKRD31	protein_coding	CTD-2235C13.2	antisense	G	A	0.07				CACD, Klf4, SEF-1, YY1		ILMN_1657871	RSAD2	2	2.57E-13		
rs77317693	5	115326824	LVRN	protein_coding, intronic					C	T	0.05				CDP		ILMN_1712431	FAM113B	12	1.58E-13	-	T cell receptor signaling pathway
																	ILMN_1669927	ICOS	2	8.26E-14		
																	ILMN_1699160	ITK	5	1.56E-14		
																	ILMN_1679185	LEF1	4	7.81E-14		
																	ILMN_2319000	MATK	19	3.11E-15		
																	ILMN_2395981	PYHIN1	1	1.29E-16		
																	ILMN_1784737	S1PR4	19	1.61E-14		
rs78022469	5	127442453	SLC12A2	protein_coding, intronic					A	G	0.08		MUS,BRN,LNG	EHMs (FAT)	Mef2		ILMN_1657871	RSAD2	2	6.92E-16		
rs182344030	5	140103718			VTRNA1-2	misc_RNA	VTRNA1-3	misc_RNA	T	C	0.05			EHMs (LIV)	ELF1, Myc, YY1, Zfx		ILMN_1705892	SH2D1A	X	7.70E-16		
rs79571133	5	127385865	CTC-228N24.3	lincRNA, intronic					A	C	0.07				Dbx1, Foxd3,Foxj2, Foxl1, HNF1, PLZF, Pou2f, Sox		ILMN_1657871	RSAD2	2	3.64E-14		
rs16879930	6	89304573			RP11-459O1.2	lincRNA	RNGTT	protein_coding	T	C	0.05				Arh::Arnf, Arnt, Dmbx1, Hic1, SREBP		ILMN_1701789	IFIT3	10	3.82E-13		
rs35480366	7	614991	PRKAR1B	protein_coding, intronic					G	A	0.06		6 tissues	EHMks (MUS, BRN, GI)	BCL, Egr-1, Rad21, Zfp691, Znf143		ILMN_2162972	LYZ	12	7.38E-14		
rs73742237	7	142170045	TRBV5-4	TR_V_gene, upstream gene variant					G	A	0.07		11 tissues	PHMs (BLD,BONE), EHMs (11 tissues)	GCNF, HNF4, RXR::LXR, TCF4, Znf143		ILMN_1729749	HERC5	4	2.67E-14	IRF1, IRF8, STAT2, STAT1, CEBPB, POLR2A**	Interferon Signaling, Antiviral mechanism by IFN-stimulated genes
																	ILMN_1760062	IFI44	1	3.24E-13		
																	ILMN_1745471	IRF9	14	9.50E-13		
																	ILMN_2054019	ISG15	1	1.20E-15		
																	ILMN_1659913	ISG20	15	3.17E-15		
																	ILMN_1674063	OAS2	12	3.88E-19		
																	ILMN_1745397	OAS3	12	9.93E-20		
																	ILMN_1691731	PARP14	3	4.04E-14		
																	ILMN_1657871	RSAD2	2	8.32E-16		
																	ILMN_1799467	SAMD9L	7	2.84E-17		
																	ILMN_1691364	STAT1	2	9.57E-14		
																	ILMN_1742618	XAF1	17	4.65E-14		
rs10505505	8	128739771	CASC11	lincRNA, intronic					T	G	0.06	TCF4	7 tissues		Fox,Foxa	1	ILMN_2319000	MATK	19	2.26E-13		
rs2094960	9	12446555			RNU2-47P	snRNA	TYRP1	protein_coding	A	G	0.06				Hoxa4, Irf, Mef2, SIX5		ILMN_1745397	OAS3	12	1.72E-13	STAT1,STAT2**	-
																	ILMN_1657871	RSAD2	2	7.62E-16		
rs72749106	9	86394748	GKAP1	protein_coding, intronic					T	C	0.05				Ap-1		ILMN_1705892	SH2D1A	X	4.12E-20		
rs1628670	10	132667323			Y_RNA	misc_RNA	MIR378C	miRNA	T	G	0.05			EHMs (GI)	Pou1f1,Sox		ILMN_1778723	AMICA1	11	2.06E-14	STAT6**	Hemostasis, Chemokine signaling pathway
																	ILMN_1677505	CCL21	9	1.40E-13		
																	ILMN_2413808	CD53	1	1.27E-13		
																	ILMN_1698367	CD84	1	2.57E-13		
																	ILMN_1725090	CTHRC1	8	7.14E-14		
																	ILMN_1702301	DOCK10	2	2.16E-14		
																	ILMN_1799725	DOCK2	5	6.12E-15		
																	ILMN_2353633	EMR2	19	4.89E-15		
																	ILMN_1712431	FAM113B	12	1.89E-14		
																	ILMN_1652825	IL10RA	11	3.81E-13		
																	ILMN_1699160	ITK	5	9.13E-13		
																	ILMN_1699644	MARCH3	5	2.68E-13		
																	ILMN_1660114	MMRN1	4	3.11E-14		
																	ILMN_1780898	PRKCH	14	1.82E-14		
																	ILMN_2383611	PTPRE	10	1.68E-14		
																	ILMN_1801504	RUNX1	21	3.55E-13		
																	ILMN_1697554	SASH3	x	5.19E-14		
																	ILMN_1717334	VAV1	19	1.90E-13		
rs1456887	11	113853470	HTR3A	protein_coding, intronic					C	T	0.05		IPSC	EHMs (SPLN)	NRSF,Rad21		ILMN_1745397	OAS3	12	2.42E-14		
rs61876940	11	9948200	SBF2/RP11-1H15.2	protein_coding, intronic/antisense					G	A	0.05			EHMs (LIV)	Mrg1::Hoxa9		ILMN_1750805	ARHGAP30	1	3.44E-14	IK2F1, RUNX1*	Chemokine signaling pathway
																	ILMN_1690907	CCR6	6	1.07E-18		
																	ILMN_1777519	ITGB7	12	4.28E-13		
																	ILMN_1699160	ITK	5	9.23E-15		
																	ILMN_1658399	KLRG1	12	1.34E-13		
																	ILMN_1705892	SH2D1A	X	4.69E-18		
																	ILMN_2319000	MATK	19	3.05E-13		
rs73004043	11	113487585			DRD2	protein_coding	TMPRSS5	protein_coding	C	T	0.05			EHMs (STRM, BONE)	F1, Hoxa7, Hoxb7, Hoxd10, Hoxd8, Ncx, Nkx6-1, F		ILMN_1674063	OAS2	12	6.05E-15	STAT1, STAT2**	Interferon alpha/beta signaling
																	ILMN_1745397	OAS3	12	4.09E-23		
																	ILMN_1657871	RSAD2	2	4.56E-14		
rs1696430	12	130499183			TMEM132D	protein_coding	RP11-474D1.4	lincRNA	G	A	0.06	CTCF,RAD21	18 tissues	PHMs(ESC, IPSC, BRN), EHMs(6 tissues)	E2A,ZEB1		ILMN_1674063	OAS2	12	3.51E-17	STAT1,STAT2,CUX1**	Interferon alpha/beta signaling
																	ILMN_1745397	OAS3	12	2.79E-15		
																	ILMN_1799467	SAMD9L	7	2.16E-13		
rs192720382	12	22315041	ST8SIA1	protein_coding, intronic					C	G	0.05			EHMs(BLD)	34 altered motifs		ILMN_1705892	SH2D1A	X	1.87E-14		
rs73226438	13	74023847	LINC00393	lincRNA, intronic					T	G	0.05		7 tissues	EHMs(5 tissues)	Barx1,Dx5, Gbx2, Hoxb6, Isx, Nobox, Pou2f, TATA		ILMN_1681301	AIM2	1	5.26E-14	MAF,SPI1, BCL, MYB, STAT6, IK2F1,GATA3, KDM2B, SAI, E2A, UTX**	Immune System, T cell receptor signaling pathway, Cytokine-cytokine receptor interaction, Jak-STAT signaling pathway
																	ILMN_1770673	AKNA	9	1.13E-13		
																	ILMN_1778723	AMICA1	11	1.84E-14		
																	ILMN_1777998	ARHGAP25	2	2.03E-16		
																	ILMN_1750805	ARHGAP30	1	1.03E-21		
																	ILMN_1663916	ARHGAP9	12	3.03E-15		
																	ILMN_2098616	C5orf39	5	4.90E-16		
																	ILMN_1690907	CCR6	6	2.60E-29		
																	ILMN_2335754	CD1E	1	1.85E-15		
																	ILMN_1698367	CD84	1	1.50E-17		
																	ILMN_1768482	CD8A	2	6.88E-16		
																	ILMN_2415303	CLEC10A	17	5.81E-17		
																	ILMN_1713749	CORO1A	16	3.72E-14		
																	ILMN_2129505	CYBASC3	11	7.34E-14		
																	ILMN_1712431	FAM113B	12	4.71E-18		
																	ILMN_1726597	FAM65B	6	2.09E-16		
																	ILMN_2366330	FERMT3	11	1.24E-14		
																	ILMN_1669927	ICOS	2	3.87E-16		
																	ILMN_1813572	IL16	15	2.18E-15		
																	ILMN_1684349	IL2RB	22	7.09E-18		
																	ILMN_2342579	IL7R	5	9.63E-15		
																	ILMN_1777519	ITGB7	12	1.44E-23		
																	ILMN_1699160	ITK	5	7.60E-24		
																	ILMN_1658399	KLRG1	12	7.89E-23		
																	ILMN_1679185	LEF1	4	1.39E-16		
																	ILMN_2162972	LYZ	12	1.03E-19		
																	ILMN_1665943	MAP4K1	19	4.47E-21		
																	ILMN_2319000	MATK	19	3.82E-21		
																	ILMN_1717207	MMP25	16	2.64E-17		
																	ILMN_1692295	MYO1G	7	1.50E-18		
																	ILMN_1738675	PTPN6	12	1.25E-14		
																	ILMN_1672417	PTPRCAP	11	4.29E-18		
																	ILMN_2395981	PYHIN1	1	2.42E-22		
																	ILMN_2255579	RAB37	17	5.31E-17		
																	ILMN_2362902	RASSF5	1	8.90E-15		
																	ILMN_1787461	RUNX3	1	1.34E-16		
																	ILMN_1784737	S1PR4	19	4.52E-18		
																	ILMN_2196078	SLAMF6	1	9.18E-20		
																	ILMN_1778599	SP140	2	2.05E-16		
																	ILMN_1785202	STAT4	2	6.03E-16		
																	ILMN_1765109	TNFRSF25	1	8.37E-16		
																	ILMN_1705892	SH2D1A	X	8.61E-27		
																	ILMN_1697554	SASH3	X	3.03E-19		
																	ILMN_1748283	PIM2	X	1.76E-16		
																	ILMN_1662026	BTK	X	1.14E-14		
																	ILMN_2112256	TNFRSF4	1	7.65E-17		
rs11159307	14	78370235	ADCK1	protein_coding, intronic					A	C	0.32			EHMs(BLD)	xp1, GR, Gfi1, Gfi1b, Maf, Mef2, Nkx2, Pou2f2, RF	3	ILMN_1783985	COQ6	14	7.11E-14		
rs12894373	14	34143440	NPAS3	protein_coding, intronic					A	G	0.06		BLD	EHMs(BRN)	Evi-1,Homez		ILMN_1745397	OAS3	12	1.02E-17	STAT1, STAT2**	Interferon alpha/beta signaling
																	ILMN_1657871	RSAD2	2	1.68E-15		
rs112549897	16	29832950	PAGR1/MVP	protein_coding, intronic					C	G	0.06		25 tissues	PHMs (19 tissues), EHMs(8 tissues)	BCL	6	ILMN_1745397	OAS3	12	3.38E-16	STAT1, STAT2**	Interferon alpha/beta signaling
																	ILMN_1657871	RSAD2	2	1.64E-13		
rs72782371	16	27238910	NSMCE1	protein_coding, intronic					C	T	0.05		BLD,OVRY	EHMs(7 tissues)	AhR, bATF, NF-E2, Pax-6, Sin3Ak-20		ILMN_1664543	IFIT3	10	5.42E-18	STAT1,STAT2,IRF8**	Interferon alpha/beta signaling
																	ILMN_1745397	OAS3	12	6.01E-18		
																	ILMN_1657871	RSAD2	2	8.94E-18		
rs11078364	17	16936529			RP11-416I2.1	lincRNA	MPRIP	protein_coding	G	A	0.06			EHMs(6 tissues)	Egr-1,Sp4	3	ILMN_1745397	OAS3	12	5.75E-14		
rs16973141	17	66625824	RP11-118B18.1	lincRNA, intronic					C	T	0.05			EHMs(ESDR)	Hlx1	2	ILMN_2408851	ARHGAP30	1	3.84E-14	BCL11A, CEBPB**	Arrhythmogenic right ventricular cardiomyopathy
																	ILMN_1690907	CCR6	6	2.50E-18		
																	ILMN_1777519	ITGB7	12	3.48E-14		
																	ILMN_1658399	KLRG1	12	5.43E-14		
																	ILMN_1679185	LEF1	4	3.41E-17		
																	ILMN_1705892	SH2D1A	X	9.45E-16		
																	ILMN_1692295	MYO1G	7	5.58E-15		
rs4985723	17	16998241	MPRIP	protein_coding, intronic					A	T	0.06			PHMs(IPSC), EHMs (8tissues)	bx1, Ebi-1, Foxp1, Mef2, Ncx, PLZF, TATA, Zfp10	5	ILMN_2054019	ISG15	1	1.38E-13	STAT1,STAT2,RELA**	Interferon alpha/beta signaling
																	ILMN_1674063	OAS2	12	1.43E-14		
																	ILMN_1745397	OAS3	12	2.48E-16		
rs77052944	17	17111905	MPRIP/RP11-45M22.	tein_coding, intronic/protein_coding, intronic					C	T	0.05				E2A,TBX5,ZEB1	5	ILMN_1657871	RSAD2	2	5.55E-14		
rs7227296	18	44528456	KATNAL2	protein_coding, intronic					T	C	0.08				ERalpha-a, HNF4, NR4A, SREBP, STAT	1	ILMN_1659990	C7orf68	7	3.47E-13		

SNP	Chromosome	Position	**Overlapped Gene**	**Type**	**Nearest Upstream Gene**	**Type**	**Nearest Downstream Gene**	**Type**	A1	A2	MAF	latory proteins b	Chromatin structure (DNAse sensitive)	chromatin state	TF binding motifs altered by the variant	Previous eQTL hits	Probe	Gene	Gene_chromosome	Interaction P-value	TF enriched	Pathways enriched
rs2072967	20	40003221			EMILIN3	protein_coding	RP4-620E11.5	pseudogene	G	A	0.05			EHMs(6 tissues)	ERalpha-a, MZF1::1-4, PRDM1, RXRA	1	ILMN_1681301	AIM2	1	1.07E-13	MAF, SPI1, BCL11A, MYB, STAT6, IK2F1,GATA3, KDM2B, SAI**	Immune System, Immunoregulatory interactions between a Lymphoid and a non-Lymphoid cell
																	ILMN_1770673	AKNA	9	1.50E-13		
																	ILMN_1778723	AMICA1	11	1.02E-14		
																	ILMN_1777998	ARHGAP25	2	2.77E-16		
																	ILMN_1750805	ARHGAP30	1	3.45E-22		
																	ILMN_1663916	ARHGAP9	12	3.05E-15		
																	ILMN_2098616	C5orf39	5	2.07E-14		
																	ILMN_1712532	CARD9	9	1.75E-13		
																	ILMN_1677505	CCL21	9	5.60E-13		
																	ILMN_1690907	CCR6	6	1.97E-29		
																	ILMN_2335754	CD1E	1	5.50E-17		
																	ILMN_1698367	CD84	1	9.04E-18		
																	ILMN_1768482	CD8A	2	1.56E-17		
																	ILMN_2415303	CLEC10A	17	3.72E-16		
																	ILMN_1713749	CORO1A	16	2.34E-14		
																	ILMN_1702301	DOCK10	2	1.81E-14		
																	ILMN_1799725	DOCK2	5	2.42E-13		
																	ILMN_1712431	FAM113B	12	5.86E-20		
																	ILMN_1726597	FAM65B	6	2.46E-17		
																	ILMN_2366330	FERMT3	11	1.03E-14		
																	ILMN_1669927	ICOS	2	2.06E-14		
																	ILMN_1813572	IL16	15	3.13E-18		
																	ILMN_1684349	IL2RB	22	4.59E-19		
																	ILMN_2342579	IL7R	5	1.37E-14		
																	ILMN_1777519	ITGB7	12	7.57E-21		
																	ILMN_1699160	ITK	5	1.64E-24		
																	ILMN_1658399	KLRG1	12	4.18E-23		
																	ILMN_1679185	LEF1	4	1.98E-16		
																	ILMN_1677920	LTF	3	4.72E-14		
																	ILMN_2162972	LYZ	12	1.65E-20		
																	ILMN_1665943	MAP4K1	19	7.68E-22		
																	ILMN_2319000	MATK	19	1.18E-24		
																	ILMN_1717207	MMP25	16	1.66E-20		
																	ILMN_1692295	MYO1G	7	1.90E-19		
																	ILMN_1795762	PLEK	2	1.23E-13		
																	ILMN_1672417	PTPRCAP	11	7.59E-18		
																	ILMN_2395981	PYHIN1	1	9.96E-23		
																	ILMN_2255579	RAB37	17	5.88E-18		
																	ILMN_2375319	RASGRP2	11	3.04E-13		
																	ILMN_2362902	RASSF5	1	4.28E-15		
																	ILMN_1787461	RUNX3	1	2.42E-16		
																	ILMN_1784737	S1PR4	19	1.70E-18		
																	ILMN_2196078	SLAMF6	1	2.80E-20		
																	ILMN_1778599	SP140	2	1.10E-18		
																	ILMN_1785202	STAT4	2	1.29E-16		
																	ILMN_1711383	STK4	20	2.19E-14		
																	ILMN_1765109	TNFRSF25	1	2.00E-16		
																	ILMN_2112256	TNFRSF4	1	4.66E-15		
																	ILMN_1705892	SH2D1A	X	7.48E-28		
																	ILMN_1697554	SASH3	X	5.09E-19		
																	ILMN_1748283	PIM2	X	1.69E-18		
																	ILMN_1662026	BTK	X	8.75E-17		

Supplementary Table 7

List of genes with significant sex difference in array based expression in RCC tissues

Autosomes

Probe	Chromosome	Symbol	Log Fold Change	P Value	Q Value
ILMN_3280952	5	LOC391777	-0.19	5.42E-33	4.79E-30
ILMN_1689425	11	DNAJB13	0.37	2.51E-28	1.78E-25
ILMN_1671565	6	RNASET2	0.81	8.94E-27	6.17E-24
ILMN_1790555	7	CCDC146	1.25	2.22E-25	1.44E-22
ILMN_1815500	6	ITPR3	0.57	5.75E-21	3.52E-18
ILMN_1712755	1	LRRC41	0.52	1.57E-17	8.67E-15
ILMN_3263469	20	LOC100130623	0.09	6.15E-17	3.31E-14
ILMN_1737110	1	LOC651957	0.57	2.65E-16	1.33E-13
ILMN_3239775	11	ODZ4	0.35	3.49E-16	1.71E-13
ILMN_2232936	1	UQCRH	-0.50	7.74E-15	3.56E-12
ILMN_1815308	2	SDC1	0.56	4.97E-14	2.19E-11
ILMN_3199929	11	LOC390183	-0.17	5.87E-14	2.54E-11
ILMN_2103480	19	ZNF320	0.35	6.47E-14	2.75E-11
ILMN_1668312	4	SLC2A9	0.37	7.5E-14	3.12E-11
ILMN_1690209	1	C1orf186	0.56	4.66E-13	1.9E-10
ILMN_2339835	9	PTGS1	0.66	6.91E-13	2.77E-10
ILMN_3201216	10	LOC441550	-0.12	7.11E-13	2.8E-10
ILMN_1688318	20	MGC72104	0.26	9.14E-13	3.54E-10
ILMN_1718136	1	UQCRHL	-0.40	1.77E-12	6.75E-10
ILMN_1709377	19	ZNF28	0.19	2.59E-12	9.67E-10
ILMN_1663490	19	ZNF541	0.37	4.59E-12	1.66E-09
ILMN_1751495	13	FGF14	0.07	1.33E-11	4.44E-09
ILMN_1767113	2	AOX1	0.58	1.56E-11	5.13E-09
ILMN_1664024	2	APOB	0.57	1.9E-11	6.16E-09
ILMN_1781859	4	UGT2B28	0.18	2.43E-11	7.77E-09
ILMN_1663716	10	PCDH21	0.33	2.89E-11	9.12E-09
ILMN_1799887	1	CTSE	0.21	3.72E-11	1.15E-08
ILMN_3245458	1	SNORA61	0.33	5.28E-11	1.55E-08
ILMN_2174805	17	CD300LG	-0.27	5.19E-11	1.55E-08
ILMN_1724686	3	CLDN1	0.57	5.49E-11	1.59E-08
ILMN_2412046	2	TRIM54	0.56	1.02E-10	2.88E-08
ILMN_1802192	10	C10orf99	0.74	1.26E-10	3.47E-08
ILMN_1746359	12	RERG	0.44	1.37E-10	3.74E-08
ILMN_1685125	20	LOC642236	0.11	2.93E-10	7.78E-08
ILMN_1751400	17	SKAP1	0.37	7.82E-10	1.98E-07
ILMN_2111932	1	SERINC2	0.35	1.14E-09	2.86E-07
ILMN_3247082	2	FAM150B	0.62	1.22E-09	3.02E-07
ILMN_1814327	3	AGTR1	-0.35	1.63E-09	3.91E-07
ILMN_1718866	5	C5orf46	0.81	2.67E-09	6.27E-07
ILMN_2180239	21	DOPEY2	0.29	2.88E-09	6.69E-07
ILMN_1766083	11	URAT1	-0.45	4.61E-09	1.06E-06
ILMN_1804415	12	SMAGP	0.23	4.64E-09	1.06E-06
ILMN_1791328	2	STK39	0.40	5.28E-09	1.18E-06
ILMN_2338452	14	SERPINA1	0.68	5.59E-09	1.23E-06
ILMN_1808487	10	PLA2G12B	0.39	6.06E-09	1.32E-06
ILMN_2215639	2	TUBA3D	0.66	7.34E-09	1.59E-06
ILMN_1681601	3	SUCNR1	-0.19	8.51E-09	1.8E-06
ILMN_1661657	11	TRPC2	0.19	9.05E-09	1.9E-06
ILMN_2193717	1	RAB42	0.09	9.47E-09	1.97E-06
ILMN_1773006	8	FABP4	-0.52	1.32E-08	2.67E-06
ILMN_1761793	22	APOL1	0.25	1.32E-08	2.67E-06
ILMN_1673421	1	LOC440704	0.25	1.35E-08	2.7E-06
ILMN_1755414	12	C12orf36	0.31	1.54E-08	3.03E-06
ILMN_1669802	3	CYP8B1	0.29	1.62E-08	3.15E-06
ILMN_1760247	19	CD70	0.64	1.77E-08	3.39E-06
ILMN_1730777	17	KRT19	0.82	1.89E-08	3.56E-06
ILMN_1760315	11	VWCE	0.34	1.97E-08	3.69E-06
ILMN_2061446	3	AADACL1	0.25	2.07E-08	3.83E-06
ILMN_2165354	13	DCLK1	0.30	2.29E-08	4.2E-06
ILMN_2216815	6	MAP7	-0.35	2.73E-08	4.98E-06
ILMN_3251346	11	NOX4	-0.24	3.04E-08	5.45E-06
ILMN_1689059	19	ZNF329	-0.15	3.18E-08	5.66E-06
ILMN_1699071	21	C21orf7	0.38	3.38E-08	5.97E-06
ILMN_3284137	13	LOC646208	0.16	3.41E-08	5.97E-06
ILMN_1676247	20	JPH2	0.13	3.58E-08	6.22E-06
ILMN_1703511	3	PDZRN3	-0.09	3.8E-08	6.55E-06
ILMN_2414878	9	STXBP1	-0.17	4.54E-08	7.71E-06
ILMN_3200484	19	LOC126235	-0.05	5.29E-08	8.91E-06
ILMN_2068499	8	POU5F1P1	0.26	5.76E-08	9.55E-06
ILMN_1751086	11	ATL3	0.20	5.81E-08	9.57E-06
ILMN_1806293	12	LIN7A	-0.19	6.07E-08	9.92E-06
ILMN_1787815	20	TRIB3	0.47	6.46E-08	1.04E-05
ILMN_1735124	20	OXT	0.13	6.74E-08	1.08E-05
ILMN_1692591	9	RNF183	0.18	7.21E-08	1.14E-05
ILMN_1653501	3	SEMA3B	0.18	7.31E-08	1.15E-05
ILMN_1652407	20	ZMYND8	0.16	8.29E-08	0.000013
ILMN_1738796	17	PITPNC1	-0.09	1.03E-07	1.59E-05
ILMN_1789040	13	SLITRK5	-0.19	1.09E-07	1.66E-05
ILMN_1655904	1	ROR1	-0.10	1.07E-07	1.66E-05
ILMN_1679060	6	LOC642559	0.30	1.36E-07	2.04E-05
ILMN_1657234	2	CCL20	0.61	1.4E-07	2.08E-05
ILMN_1796179	6	HIST1H2BK	0.38	1.56E-07	2.29E-05
ILMN_1751276	11	BDNF	-0.19	1.6E-07	2.34E-05
ILMN_1719646	19	ZIK1	-0.07	1.69E-07	2.45E-05
ILMN_1669338	1	CYP4X1	-0.07	1.73E-07	2.48E-05
ILMN_1795336	10	PTER	0.42	1.86E-07	2.63E-05
ILMN_1684210	1	NPAL3	0.17	2.06E-07	2.88E-05
ILMN_1733831	1	PGBD5	-0.24	2.36E-07	3.25E-05
ILMN_1696317	12	CACNA2D4	0.15	2.39E-07	3.27E-05
ILMN_1785852	2	OBFC2A	0.21	2.72E-07	3.66E-05
ILMN_1763638	1	BCAR3	-0.20	2.92E-07	3.87E-05
ILMN_1758825	4	ABLIM2	0.17	2.93E-07	3.87E-05
ILMN_2255579	17	RAB37	0.16	3.03E-07	3.95E-05
ILMN_1713266	1	FAM46C	0.34	3.27E-07	4.25E-05
ILMN_3307505	11	MGC34821	-0.06	3.43E-07	4.43E-05
ILMN_1746720	18	TTC39C	0.15	3.5E-07	0.000045
ILMN_2245305	20	ABHD12	0.17	3.85E-07	4.89E-05
ILMN_1756049	12	NT5DC3	0.31	4.13E-07	5.21E-05
ILMN_1776181	11	BIRC3	0.43	4.25E-07	5.32E-05
ILMN_1663281	19	ZNF702P	0.20	4.27E-07	5.32E-05
ILMN_3307693	20	WFDC2	0.49	4.71E-07	5.83E-05
ILMN_2355831	2	FHL2	0.40	4.92E-07	6.06E-05
ILMN_1718537	10	HPS6	-0.11	5.17E-07	6.31E-05

Sex-Chromosomes

Probe	Chromosome	Symbol	Log Fold Change	P Value	Q Value	Genes described as escaping X-inactivation or having a functional Y-homolog (Dunford et al., 2017)
ILMN_1783142	Y	RPS4Y1	4.01	6.85E-187	1.51E-182	
ILMN_1764573	X	XIST	-2.38	2.31E-162	2.55E-158	
ILMN_1755537	Y	EIF1AY	1.98	2.78E-142	2.04E-138	
ILMN_1685690	Y	JARID1D	0.96	3.16E-141	1.74E-137	
ILMN_3238417	Y	LOC100133662	2.40	3.12E-129	1.38E-125	
ILMN_1670821	Y	CYorf15A	1.06	4.14E-122	1.52E-118	
ILMN_2191331	Y	RPS4Y2	0.92	4.35E-99	1.37E-95	
ILMN_1739587	Y	UTY	0.61	2.71E-98	7.46E-95	
ILMN_2210199	Y	NLGN4Y	0.45	6.41E-89	1.41E-85	
ILMN_1772163	Y	PRKY	0.40	4.61E-88	9.25E-85	
ILMN_1756506	Y	CYorf15B	0.37	5.34E-75	9.06E-72	
ILMN_2056795	Y	USP9Y	0.24	9.57E-64	1.51E-60	
ILMN_2052433	Y	CYorf14	0.29	5.79E-60	8.52E-57	
ILMN_1654488	X	KDM6A/UTX	-0.32	1.57E-53	2.16E-50	ESCAPE gene with functional Y homolog
ILMN_1776195	Y	TMSB4Y	0.22	1.45E-51	1.78E-48	
ILMN_2371700	X	UCHL5IP	1.16	1.11E-50	1.29E-47	
ILMN_2143383	Y	TTTY14	0.21	3.70E-48	4.08E-45	
ILMN_2077896	Y	TTTY15	0.12	9.46E-39	9.94E-36	
ILMN_1704431	X	LOC554203	-0.31	2.63E-34	2.42E-31	
ILMN_1710136	X	HDHD1A	-0.31	6.49E-33	5.51E-30	ESCAPE gene
ILMN_1794392	X	DDX3X	-0.41	2.43E-32	1.98E-29	ESCAPE gene with functional Y homolog
ILMN_1687484	X	ZFX	-0.24	2.65E-32	2.09E-29	ESCAPE gene with functional Y homolog
ILMN_2166831	X	RPS4X	-0.40	2.61E-30	1.98E-27	ESCAPE gene with functional Y homolog
ILMN_1804958	Y	ZFY	0.10	1.71E-29	1.26E-26	
ILMN_1684873	X	ARSD	-0.36	5.35E-21	3.37E-18	ESCAPE gene
ILMN_1813240	X	EIF1AX	-0.26	5.57E-20	3.32E-17	ESCAPE gene with functional Y homolog
ILMN_1663640	X	MAOA	0.84	7.79E-20	4.52E-17	ESCAPE gene
ILMN_1680892	X	TREX2	0.12	1.72E-18	9.72E-16	
ILMN_1798224	X	KDM5C	-0.08	1.81E-16	9.31E-14	ESCAPE gene with functional Y homolog
ILMN_1773868	X	ZRSR2	-0.25	5.04E-16	2.42E-13	ESCAPE gene
ILMN_1673275	X	TRAPPC2	-0.19	8.58E-15	3.86E-12	
ILMN_1805512	X	WDR13	0.26	3.12E-12	1.15E-09	
ILMN_2080080	X	MAP7D2	0.64	7.91E-12	2.73E-09	
ILMN_1665717	X	EIF2S3	-0.21	1.24E-11	4.20E-09	ESCAPE gene
ILMN_1664348	X	PNPLA4	-0.14	1.06E-10	2.96E-08	ESCAPE gene
ILMN_1748116	X	GEMIN8	-0.11	2.81E-10	7.56E-08	
ILMN_1652006	X	SMC1A	-0.09	5.00E-10	1.28E-07	ESCAPE gene
ILMN_1697189	X	PNCK	0.95	1.49E-09	3.65E-07	
ILMN_1728540	X	FUNDC1	-0.16	4.70E-09	1.06E-06	ESCAPE gene
ILMN_1683609	X	UBE1	-0.24	1.66E-08	3.22E-06	
ILMN_1672807	X	CA5B	-0.11	1.79E-08	3.41E-06	ESCAPE gene
ILMN_1806349	X	SLC6A8	0.43	2.44E-07	3.30E-05	
ILMN_1729515	X	PIN4	-0.08	2.44E-07	3.30E-05	
ILMN_1656934	X	REPS2	0.30	2.86E-07	3.82E-05	
ILMN_1681737	X	TMSB15A	-0.24	3.63E-07	4.63E-05	
ILMN_2373728	X	ASB9	-0.11	1.25E-06	0.0001	
ILMN_1687592	X	WWC3	0.14	1.41E-06	0.0001	
ILMN_1715638	X	GAGE4	-0.11	7.08E-06	0.0005	
ILMN_1748283	X	PIM2	0.23	7.48E-06	0.0005	
ILMN_2124241	X	MUM1L1	0.15	8.75E-06	0.0006	
ILMN_1806198	X	VSIG1	0.09	9.53E-06	0.0006	
ILMN_2134855	X	LOC728758	-0.06	1.04E-05	0.0007	
ILMN_1754528	Y	DAZ4	0.06	1.30E-05	0.0008	
ILMN_1664001	X	ARSF	-0.16	2.38E-05	0.0014	
ILMN_1757537	X	RGAG4	-0.05	2.63E-05	0.0015	
ILMN_1656165	X	USP9X	-0.10	2.93E-05	0.0016	ESCAPE gene with functional Y homolog
ILMN_1765860	X	DOCK11	0.21	4.24E-05	0.0021	
ILMN_3217172	X	LOC286444	0.14	9.36E-05	0.0036	
ILMN_3238818	X	LOC649076	0.09	0.0001	0.0049	
ILMN_1676348	X	CXorf36	-0.07	0.0002	0.0052	
ILMN_1684771	X	PGRMC1	-0.19	0.0002	0.0054	
ILMN_3202024	X	LOC392437	0.17	0.0002	0.0055	
ILMN_1809291	X	TSPAN7	-0.24	0.0002	0.0057	
ILMN_1806473	X	BEX5	-0.18	0.0002	0.0060	
ILMN_2334760	X	ARMCX3	-0.15	0.0002	0.0071	
ILMN_1800253	X	ZNF711	-0.04	0.0002	0.0072	
ILMN_1701604	X	TBC1D8B	0.09	0.0003	0.0084	
ILMN_1798546	X	HDAC6	0.13	0.0003	0.0088	
ILMN_1738885	X	WDR40B	0.05	0.0004	0.0101	
ILMN_2376263	X	SMARCA1	-0.11	0.0004	0.0105	
ILMN_1779370	X	ARHGEF9	-0.04	0.0004	0.0108	
ILMN_1756261	X	GPR143	0.12	0.0005	0.0112	ESCAPE gene
ILMN_3248906	X	ZC4H2	-0.06	0.0005	0.0113	
ILMN_1773117	X	BCOR	-0.10	0.0006	0.0129	
ILMN_2362245	X	HNRNPH2	-0.14	0.0007	0.0146	
ILMN_1750051	X	AMER1	-0.07	0.0007	0.0156	
ILMN_1690327	X	CLCN5	-0.07	0.0008	0.0169	
ILMN_2373566	X	PJA1	-0.09	0.0009	0.0180	
ILMN_2367070	X	ACOT9	0.08	0.0009	0.0180	
ILMN_1806914	X	FATE1	-0.08	0.0010	0.0194	
ILMN_3240003	Y	LOC100133012	0.07	0.0011	0.0202	
ILMN_3226505	X	MSL3	0.13	0.0011	0.0203	ESCAPE gene
ILMN_1794803	X	NDP	0.08	0.0011	0.0209	
ILMN_1810210	X	DACH2	-0.03	0.0012	0.0220	
ILMN_3239383	X	CA5BP	-0.03	0.0014	0.0241	
ILMN_2279961	X	LAMP2	0.11	0.0018	0.0283	
ILMN_3283155	X	LOC642357	0.06	0.0019	0.0293	
ILMN_2370241	X	TEX11	0.27	0.0020	0.0303	
ILMN_1662557	X	MAP7D3	0.04	0.0021	0.0307	
ILMN_1784822	X	PPP1R3F	-0.05	0.0022	0.0317	
ILMN_1775742	X	RNF128	0.16	0.0023	0.0336	
ILMN_1689353	X	APLN	-0.16	0.0025	0.0348	
ILMN_1708131	X	BHLHB9	-0.06	0.0026	0.0358	
ILMN_1738767	X	PLP2	0.09	0.0027	0.0372	
ILMN_2379130	X	IRAK1	0.10	0.0028	0.0379	
ILMN_1679299	X	IGSF1	-0.04	0.0030	0.0397	
ILMN_2349071	X	GPR64	0.18	0.0030	0.0401	
ILMN_1687840	X	ABCB7	0.09	0.0031	0.0404	
ILMN_1732831	X	CHST7	-0.11	0.0032	0.0414	
ILMN_1711566	X	TIMP1	0.20	0.0032	0.0419	
ILMN_1803824	X	ZDHHC9	-0.12	0.0035	0.0436	
ILMN_3293694	Y	LOC727768	0.07	0.0038	0.0460	
ILMN_2160428	X	IL1RAPL1	-0.04	0.0041	0.0474	

ILMN_1740180	6	SNX3		-0.19	5.31E-07	6.44E-05
ILMN_2325337	22	APOL2		0.29	5.51E-07	6.64E-05
ILMN_1674344	18	PARD6G		-0.14	5.64E-07	6.76E-05
ILMN_1722127	8	RAD54B		0.13	5.67E-07	6.76E-05
ILMN_1652464	2	TUBA3E		0.35	5.84E-07	6.92E-05
ILMN_2402556	8	ZHX1		-0.08	6.13E-07	7.24E-05
ILMN_1767474	1	HAO2		-0.14	6.7E-07	7.86E-05
ILMN_3243117	14	TTC9		-0.05	6.83E-07	7.97E-05
ILMN_1683146	11	FTH1		-0.18	7.11E-07	8.21E-05
ILMN_1719661	16	SEPX1		0.28	7.19E-07	8.26E-05
ILMN_1753913	1	GPR177		-0.15	7.39E-07	8.44E-05
ILMN_1711120	8	ARC		-0.04	7.86E-07	8.94E-05
ILMN_1733421	10	PRKCQ		0.14	8.21E-07	9.29E-05
ILMN_1659462	1	DUSP23		0.25	8.27E-07	9.31E-05
ILMN_1691506	15	NGRN		-0.14	8.72E-07	9.77E-05
ILMN_1670779	16	DPEP1		0.18	8.82E-07	9.82E-05
ILMN_1808117	12	C1QL4		-0.32	9.25E-07	0.000103
ILMN_1784863	7	CD36		-0.38	1.01E-06	0.000111
ILMN_1737398	15	PTPLAD1		-0.18	1.03E-06	0.000113
ILMN_1795976	10	SFXN2		-0.10	1.06E-06	0.000115
ILMN_1683112	9	FANCC		-0.11	1.09E-06	0.000118
ILMN_2411236	7	NRCAM		0.31	1.12E-06	0.000119
ILMN_2172755	8	DEPDC6		0.18	1.11E-06	0.000119
ILMN_1679093	19	ZNF581		0.18	1.11E-06	0.000119
ILMN_1800225	3	PPARG		-0.21	1.2E-06	0.000127
ILMN_2362681	16	CES2		0.28	1.27E-06	0.000132
ILMN_1704629	1	SLC1A7		0.11	1.27E-06	0.000132
ILMN_1729123	9	PPP2R4		0.19	1.3E-06	0.000134
ILMN_1811364	2	SGPP2		0.27	1.33E-06	0.000136
ILMN_1664068	5	ERGIC1		0.19	1.34E-06	0.000137
ILMN_1760593	12	CRY1		-0.13	1.37E-06	0.000139
ILMN_1815203	1	HMGCS2		-0.58	1.38E-06	0.00014
ILMN_1745599	5	LOC643977		0.12	1.41E-06	0.000141
ILMN_2408946	15	PDE8A		-0.07	1.42E-06	0.000141
ILMN_1681462	2	REG1B		0.38	1.44E-06	0.000144
ILMN_1654737	9	TRIM32		-0.08	1.46E-06	0.000144
ILMN_1716384	2	ATL2		-0.10	1.5E-06	0.000148
ILMN_1806787	22	CSDC2		-0.24	1.55E-06	0.000152
ILMN_1763641	19	ZNF614		-0.16	1.56E-06	0.000152
ILMN_1714567	11	AHNAK		0.21	1.59E-06	0.000154
ILMN_2182850	11	C11orf85		0.08	1.6E-06	0.000155
ILMN_1658464	13	GTF3A		0.16	1.78E-06	0.00017
ILMN_1659106	1	PHLDA3		0.20	1.84E-06	0.000174
ILMN_3274601	1	LOC343184		0.10	1.85E-06	0.000174
ILMN_1785191	6	TMEM14A		-0.24	1.91E-06	0.00018
ILMN_1662880	5	FIS		0.36	1.96E-06	0.000183
ILMN_1804051	7	SNX8		0.13	2.04E-06	0.00019
ILMN_1693293	12	LOC648176		0.06	2.32E-06	0.000215
ILMN_1685493	12	TMCC3		-0.13	2.36E-06	0.000218
ILMN_1796835	1	RWDD3		-0.10	2.51E-06	0.00023
ILMN_1699665	21	CLIC6		0.51	2.59E-06	0.000234
ILMN_1658044	8	LOC648814		0.26	2.56E-06	0.000234
ILMN_3223871	7	LOC729920		0.09	2.57E-06	0.000234
ILMN_2374362	9	FAM108B1		-0.08	2.61E-06	0.000235
ILMN_1664175	1	VAMP4		-0.09	2.64E-06	0.000237
ILMN_2348975	1	NASP		-0.10	2.69E-06	0.00024
ILMN_1804328	8	WWP1		-0.15	2.97E-06	0.000263
ILMN_1769911	12	SLC38A1		0.24	3.02E-06	0.000266
ILMN_1717572	11	PGA5		0.30	3.04E-06	0.000268
ILMN_1704398	7	FZD9		-0.08	3.12E-06	0.000273
ILMN_3239629	19	LOC729679		0.22	3.16E-06	0.000275
ILMN_2086238	17	SMYD4		0.13	3.17E-06	0.000275
ILMN_1789648	6	SCGN		-0.50	3.21E-06	0.000278
ILMN_1796801	17	ABCA8		-0.16	3.24E-06	0.000279
ILMN_1697922	4	LOC645671		0.05	3.28E-06	0.000281
ILMN_1747744	5	LHFPL2		0.24	3.33E-06	0.000284
ILMN_3203361	17	LOC644070		0.20	3.44E-06	0.000292
ILMN_1808500	2	CEP68		-0.10	3.45E-06	0.000292
ILMN_2162819	4	UGT2B11		0.33	3.51E-06	0.000297
ILMN_1801460	4	LOC651647		-0.06	3.58E-06	0.000301
ILMN_1751393	1	ZNF684		-0.11	3.66E-06	0.000307
ILMN_1672057	9	AQP7P2		-0.14	3.68E-06	0.000308
ILMN_1764629	8	SLC39A14		0.35	3.74E-06	0.000311
ILMN_1713995	12	SCNN1A		0.50	3.79E-06	0.000313
ILMN_1815938	9	TRPM3		-0.05	3.79E-06	0.000313
ILMN_1772627	4	D4S234E		0.22	3.85E-06	0.000317
ILMN_1797277	2	KIF3C		-0.06	3.91E-06	0.000321
ILMN_3245062	4	SHROOM3		0.19	3.94E-06	0.000322
ILMN_3211132	17	LOC645173		0.22	3.96E-06	0.000322
ILMN_1737663	7	C7orf13		0.08	4.18E-06	0.000339
ILMN_1683844	4	HSD17B13		-0.06	4.3E-06	0.000346
ILMN_1655521	9	DMRTA1		-0.13	4.37E-06	0.000351
ILMN_1677680	3	FGF12		-0.10	4.49E-06	0.000359
ILMN_1677487	2	ANKZF1		0.13	4.66E-06	0.000371
ILMN_1666269	20	CTSZ		0.28	4.86E-06	0.000386
ILMN_1722820	22	KDELR3		0.27	4.9E-06	0.000387
ILMN_1740269	1	WNT2B		0.17	4.97E-06	0.000391
ILMN_1815885	19	ZFP112		-0.08	4.97E-06	0.000391
ILMN_1671969	2	UGP2		0.17	5.05E-06	0.000395
ILMN_1672022	2	EPHA4		-0.16	5.18E-06	0.000404
ILMN_1749009	11	REXO2		-0.17	5.32E-06	0.000411
ILMN_2400322	1	DYRK3		-0.08	5.31E-06	0.000411
ILMN_1724052	19	LOC730051		-0.10	5.38E-06	0.000414
ILMN_2072357	1	IRF6		-0.09	5.57E-06	0.000427
ILMN_1808238	15	RBPMS2		-0.30	5.73E-06	0.000436
ILMN_1660723	19	RDH13		0.30	5.73E-06	0.000436
ILMN_1789599	1	NBL1		0.36	5.76E-06	0.000436
ILMN_1733094	7	STEAP1		-0.14	5.78E-06	0.000437
ILMN_2331231	20	TNFRSF6B		0.34	6.08E-06	0.000458
ILMN_1778444	6	FKBP5		0.36	6.17E-06	0.000462
ILMN_2232177	14	ACTN1		0.27	6.17E-06	0.000462
ILMN_1779882	5	ANKRD43		0.14	6.42E-06	0.000477
ILMN_2085362	6	FUCA2		-0.05	6.81E-06	0.000503
ILMN_1697211	3	UBE1DC1		-0.06	7.21E-06	0.000529
ILMN_1785113	6	MUT		-0.15	7.41E-06	0.000541
ILMN_1809191	1	RAP1A		-0.05	7.53E-06	0.000545
ILMN_1742846	9	DIRAS2		-0.19	7.65E-06	0.00055
ILMN_1815718	10	BTRC		-0.06	7.66E-06	0.00055
ILMN_1764885	20	C20orf75		0.17	7.9E-06	0.000566
ILMN_1783497	10	PANK1		-0.16	7.98E-06	0.00057
ILMN_2049766	7	NFE2L3		0.23	8.13E-06	0.000577
ILMN_2047430	10	5-Mar		-0.08	8.14E-06	0.000577
ILMN_1747598	6	PPP1R11		-0.13	8.21E-06	0.000578
ILMN_1726368	19	ZNF135		-0.12	8.2E-06	0.000578
ILMN_1751464	19	TNFSF9		0.12	8.24E-06	0.000579
ILMN_1758457	17	TBC1D16		0.16	8.32E-06	0.000583
ILMN_3283772	1	LOC644237		0.22	8.41E-06	0.000587
ILMN_1741960	3	DGKG		0.05	8.63E-06	0.000599
ILMN_1800311	6	HSF2		-0.07	8.73E-06	0.000603
ILMN_3261938	2	LOC100130154		0.15	9.01E-06	0.000619
ILMN_2203891	18	SMAD7		-0.15	9.16E-06	0.000625
ILMN_3199916	9	LOC100130980		0.09	9.14E-06	0.000625
ILMN_1702973	2	TMEM166		0.27	9.65E-06	0.000655
ILMN_1708798	3	EAF2		-0.14	9.9E-06	0.000668
ILMN_1656192	8	ZNF704		-0.04	9.89E-06	0.000668
ILMN_1791890	11	SPON1		-0.49	1.01E-05	0.000679
ILMN_3249477	1	CHIT1		0.13	1.02E-05	0.000682
ILMN_1808584	1	FAM36A		-0.18	1.04E-05	0.000691
ILMN_2332105	6	WRNIP1		-0.12	1.04E-05	0.000691
ILMN_3246465	1	SNORA16A		0.06	1.09E-05	0.000725
ILMN_3306852	2	LOC730908		-0.06	1.11E-05	0.000734
ILMN_2225548	18	ZNF521		0.12	1.18E-05	0.00078
ILMN_1757388	19	OCEL1		0.13	0.000012	0.000791
ILMN_1671583	7	MKRN1		0.11	1.23E-05	0.000807
ILMN_2336094	4	ODZ3		0.25	1.25E-05	0.000816
ILMN_1752502	10	HKDC1		0.28	1.26E-05	0.000818
ILMN_1702177	6	GLO1		-0.17	1.28E-05	0.000831
ILMN_3208014	1	LOC100131866		0.14	1.32E-05	0.000852
ILMN_1680251	10	SORCS1		-0.09	1.33E-05	0.000854
ILMN_1682699	6	PBX2		-0.11	0.000014	0.000892
ILMN_1697916	11	GYLTL1B		-0.05	1.43E-05	0.000909
ILMN_2274199	6	SUPT3H		-0.06	1.49E-05	0.000944
ILMN_1738604	4	ADH5		-0.13	1.49E-05	0.000946
ILMN_1652521	8	MTMR9		-0.10	1.52E-05	0.000962
ILMN_1654920	10	HNRPH3		-0.14	1.54E-05	0.000971
ILMN_1679837	14	SGPP1		-0.06	1.54E-05	0.000971
ILMN_1780756	14	RBM23		-0.12	1.58E-05	0.00099
ILMN_1758907	2	UGT1A6		-0.25	1.59E-05	0.000994
ILMN_1674024	10	IKZF5		-0.07	1.61E-05	0.001001
ILMN_1675100	13	EDNRB		-0.07	1.61E-05	0.001004
ILMN_3211935	11	LOC100132715		-0.12	1.65E-05	0.001022
ILMN_1756469	19	GAMT		0.32	1.76E-05	0.001082
ILMN_1714737	6	ASF1A		-0.10	1.83E-05	0.001117
ILMN_1754643	22	DGCR14		-0.04	1.85E-05	0.001125
ILMN_1768812	11	FXYD6		-0.22	0.000019	0.001152
ILMN_1699249	19	ZNF649		-0.08	0.000019	0.001152
ILMN_1657149	8	C8orf46		0.09	1.93E-05	0.001166
ILMN_1700232	6	FBXO30		-0.09	1.93E-05	0.001166
ILMN_1738989	8	GOLSYN		0.33	1.97E-05	0.00118
ILMN_1762766	20	PTPRA		-0.08	1.96E-05	0.00118
ILMN_1695157	17	CA4		-0.36	1.98E-05	0.001184
ILMN_1776998	15	DNAJA4		0.22	0.00002	0.001189
ILMN_2110422	7	PKD1L1		-0.03	1.99E-05	0.001189
ILMN_1690325	6	NFYA		-0.06	2.04E-05	0.001211
ILMN_1688642	9	LAMC3		-0.26	2.07E-05	0.001221
ILMN_1700695	9	SLC44A1		-0.14	2.11E-05	0.001244
ILMN_1687768	6	NCOA7		-0.21	2.15E-05	0.001261
ILMN_2371911	1	MUC1		0.38	2.25E-05	0.001312
ILMN_2041222	17	FLJ40504		0.29	2.25E-05	0.001312
ILMN_1758128	17	CYGB		-0.26	2.25E-05	0.001312
ILMN_1679134	10	NSMCE4A		-0.11	2.32E-05	0.001346
ILMN_2103107	8	ADAMDEC1		0.30	2.36E-05	0.001363
ILMN_1784113	19	NAT14		0.19	2.38E-05	0.001367
ILMN_1664466	9	KLHL9		-0.16	2.44E-05	0.0014
ILMN_1699814	4	TLL1		-0.12	2.45E-05	0.001402
ILMN_1720053	6	ZFAND3		-0.09	2.47E-05	0.001406
ILMN_1695285	2	CAPN14		0.07	2.49E-05	0.001418
ILMN_1712312	15	RAB11A		-0.11	2.51E-05	0.001426
ILMN_1663866	5	TGFBI		0.51	2.55E-05	0.001438
ILMN_1745887	12	FBXO21		0.16	2.55E-05	0.001438
ILMN_1772329	3	LRRFIP2		0.09	2.56E-05	0.001439
ILMN_1678862	10	FUT11		-0.08	2.58E-05	0.00145
ILMN_1784447	10	PLCE1		0.16	2.63E-05	0.001468
ILMN_1787514	19	CAPN12		0.27	2.66E-05	0.001481
ILMN_1734608	19	ZNF77		-0.05	2.73E-05	0.001515
ILMN_1792283	10	RPP30		-0.03	2.73E-05	0.001516
ILMN_1660973	2	GAD1		0.22	2.82E-05	0.00156
ILMN_1738494	9	AQP7		-0.06	2.83E-05	0.001562
ILMN_1764166	6	BCKDHB		-0.13	2.89E-05	0.001585
ILMN_1772131	2	IL1R2		0.38	0.000029	0.001588
ILMN_1669142	4	NARG1		-0.09	2.91E-05	0.001588
ILMN_3248703	1	SNORD99		0.15	2.99E-05	0.001621
ILMN_2100287	10	CCDC147		-0.04	3.04E-05	0.001643
ILMN_1707342	3	LRIG1		-0.12	3.06E-05	0.001644
ILMN_1720034	6	PKHD1		-0.09	3.06E-05	0.001644
ILMN_1694569	12	LOC645100		-0.06	3.06E-05	0.001644
ILMN_1739840	9	LRRC8A		-0.11	3.11E-05	0.001667
ILMN_2130180	12	RPL13L		0.15	3.13E-05	0.001669
ILMN_1813746	9	CORO2A		0.12	3.13E-05	0.001669
ILMN_1772036	7	STEAP4		-0.07	3.19E-05	0.001697
ILMN_1668426	15	SPESP1		0.12	0.000032	0.001698

ILMN_1670193	11	ACY3		0.21	3.34E-05	0.001744
ILMN_1695959	21	C21orf63		0.18	3.34E-05	0.001744
ILMN_1713756	10	GLUD1		-0.17	3.34E-05	0.001744
ILMN_1753547	17	STAT5A		0.14	3.33E-05	0.001744
ILMN_3276016	3	LOC339843		-0.10	3.33E-05	0.001744
ILMN_2382942	15	CA12		0.30	0.000034	0.001759
ILMN_1712298	8	ANKRD46		-0.14	0.000034	0.001759
ILMN_1671621	6	PCMT1		-0.13	0.000034	0.001759
ILMN_1757408	19	ZNF256		-0.11	3.38E-05	0.001759
ILMN_1686360	1	ZNF124		-0.06	3.42E-05	0.001761
ILMN_2089902	6	NUS1		-0.05	0.000035	0.001802
ILMN_1696537	4	DDIT4L		-0.38	3.56E-05	0.001823
ILMN_1707232	10	EBF3		-0.17	3.64E-05	0.001861
ILMN_1694711	6	MAD2L1BP		-0.12	3.67E-05	0.001869
ILMN_1701464	19	ZNF776		-0.05	3.69E-05	0.001872
ILMN_2221046	5	GM2A		0.12	3.78E-05	0.001909
ILMN_1786024	22	POLR3H		-0.10	3.78E-05	0.001909
ILMN_3209399	11	LOC399965		0.14	3.84E-05	0.001936
ILMN_3229424	6	LOC730101		-0.07	3.89E-05	0.001957
ILMN_1812031	19	PALM		-0.21	4.02E-05	0.002007
ILMN_1757026	6	MANEA		-0.13	4.01E-05	0.002007
ILMN_1761921	1	IL12RB2		0.09	0.00004	0.002007
ILMN_1688152	19	IL27RA		0.15	4.06E-05	0.002015
ILMN_3235171	16	CCDC64B		0.14	4.05E-05	0.002015
ILMN_1806651	5	PARP8		0.12	4.09E-05	0.002015
ILMN_1660817	11	DDB2		0.09	4.09E-05	0.002015
ILMN_1740678	6	LOC652404		0.08	4.09E-05	0.002015
ILMN_1750967	6	ZKSCAN3		-0.05	4.08E-05	0.002015
ILMN_2392261	5	FABP6		0.37	0.000041	0.002015
ILMN_1664833	9	MRPL50		-0.16	4.12E-05	0.002018
ILMN_2331658	3	C3orf17		-0.06	4.16E-05	0.002036
ILMN_2359907	17	CD68		0.24	4.24E-05	0.002058
ILMN_1665291	7	NUB1		0.11	4.23E-05	0.002058
ILMN_1764769	11	VWA5A		0.15	0.000043	0.002075
ILMN_3228294	19	LOC729279		0.17	4.31E-05	0.002076
ILMN_1661653	15	DET1		-0.07	4.39E-05	0.00211
ILMN_1741698	11	OPCML		-0.19	4.47E-05	0.002143
ILMN_1685496	1	RGS7		-0.17	4.56E-05	0.002176
ILMN_1736112	4	ARHGAP10		0.13	0.000046	0.002191
ILMN_1660871	9	NEK6		0.21	4.62E-05	0.002195
ILMN_1767068	15	SMAD6		-0.17	4.63E-05	0.002197
ILMN_1716957	17	C1QL1		0.30	4.65E-05	0.0022
ILMN_1696654	10	IFIT5		-0.09	4.76E-05	0.002247
ILMN_1735779	12	KCNJ8		-0.22	4.78E-05	0.002255
ILMN_1739847	22	EIF3D		0.10	0.000048	0.002256
ILMN_2396875	7	IGFBP3		0.36	4.81E-05	0.002258
ILMN_3287068	13	LOC728031		0.15	4.84E-05	0.002264
ILMN_2362122	10	AP3M1		-0.11	4.84E-05	0.002264
ILMN_2065783	6	EXOC2		-0.08	0.000049	0.002285
ILMN_1808677	4	UGT2B17		0.29	4.92E-05	0.00229
ILMN_1706434	16	LOC440359		0.18	5.06E-05	0.002347
ILMN_1665877	2	RNF149		0.15	5.06E-05	0.002347
ILMN_3294335	6	LOC646294		0.15	5.11E-05	0.002365
ILMN_2054392	6	PPIL1		-0.11	5.21E-05	0.002404
ILMN_2391400	4	PITX2		0.10	5.27E-05	0.002421
ILMN_3263974	9	KRT18P13		0.23	5.36E-05	0.002455
ILMN_1728799	9	FBP1		-0.30	5.52E-05	0.002523
ILMN_1656977	2	HIBCH		-0.18	5.55E-05	0.002528
ILMN_1781626	12	C1S		0.39	5.59E-05	0.002542
ILMN_1653042	16	HSD3B7		0.27	5.64E-05	0.002559
ILMN_1670064	1	C1orf210		-0.08	0.000057	0.00258
ILMN_1669881	7	TSPAN13		-0.23	5.72E-05	0.002588
ILMN_2220403	6	C6orf72		-0.14	5.74E-05	0.002588
ILMN_1724437	22	GCAT		0.17	5.88E-05	0.002645
ILMN_2044927	6	RNF5		-0.12	5.89E-05	0.002645
ILMN_1755588	19	ANKRD47		-0.11	0.000059	0.002646
ILMN_1678095	1	SMPDL3B		0.11	5.91E-05	0.002646
ILMN_1785202	2	STAT4		0.15	5.95E-05	0.002656
ILMN_1705907	6	NUP153		-0.10	5.96E-05	0.002657
ILMN_1775853	3	MAGI1		-0.06	6.02E-05	0.002678
ILMN_1716461	19	LGI4		0.35	6.06E-05	0.002691
ILMN_1719316	15	TMED3		0.16	0.000061	0.002704
ILMN_1759948	8	RNF5P1		-0.21	6.12E-05	0.002707
ILMN_1701877	19	AXL		0.21	6.15E-05	0.002707
ILMN_1672161	3	ARPP-21		-0.07	6.16E-05	0.002707
ILMN_1750029	21	GABPA		-0.11	6.17E-05	0.002707
ILMN_1667748	12	ANKRD33		0.23	6.24E-05	0.002724
ILMN_1658800	6	BRPF3		-0.10	6.25E-05	0.002724
ILMN_2392143	11	TRAF6		-0.06	6.38E-05	0.002774
ILMN_1675947	16	MT3		0.48	6.49E-05	0.002818
ILMN_1681754	8	GGH		-0.19	6.52E-05	0.002827
ILMN_1775111	7	SND1		0.13	6.55E-05	0.002832
ILMN_2413517	6	TRIM39		-0.04	6.58E-05	0.002835
ILMN_1749244	16	LYRM1		0.19	6.62E-05	0.002845
ILMN_3238326	2	RNF144A		-0.14	6.68E-05	0.002868
ILMN_1751375	4	ENAM		-0.13	6.75E-05	0.002881
ILMN_1798288	1	MOBKL2C		-0.10	6.73E-05	0.002881
ILMN_2123665	11	SBF2		-0.08	6.74E-05	0.002881
ILMN_1796245	19	DNASE2		0.14	6.78E-05	0.002888
ILMN_1705546	6	POU5F1		0.09	6.79E-05	0.002888
ILMN_1674620	7	SGCE		-0.28	6.85E-05	0.002903
ILMN_2405031	7	TRIM24		-0.06	6.86E-05	0.002903
ILMN_1744381	7	SERPINE1		0.40	6.87E-05	0.002904
ILMN_2330787	14	FRMD6		-0.10	6.89E-05	0.002905
ILMN_1782788	12	CSDA		0.15	6.92E-05	0.002913
ILMN_1773586	6	SLC17A2		-0.25	7.02E-05	0.002946
ILMN_1768809	19	ZNF134		-0.06	7.02E-05	0.002946
ILMN_1737360	20	TSPYL3		-0.16	7.17E-05	0.002997
ILMN_1709683	20	RASSF2		0.20	7.24E-05	0.003018
ILMN_1789639	1	FMOD		0.18	7.26E-05	0.003022
ILMN_3236220	19	LPAR2		0.10	7.31E-05	0.003037
ILMN_2064725	12	METTL7B		0.22	7.35E-05	0.003047
ILMN_3239500	8	FAM135B		0.08	7.49E-05	0.003096
ILMN_1695317	13	RCBTB1		-0.04	7.49E-05	0.003096
ILMN_1716524	3	RAB7A		-0.11	0.000078	0.003197
ILMN_1747546	1	TSPAN1		0.22	7.94E-05	0.00325
ILMN_1709257	21	DSCR6		-0.07	8.02E-05	0.003271
ILMN_1757262	9	ZBTB5		-0.06	8.01E-05	0.003271
ILMN_1714850	10	HSGT1		-0.07	8.09E-05	0.003294
ILMN_1704550	8	AZIN1		-0.05	8.13E-05	0.003304
ILMN_1767747	6	HDAC2		-0.13	8.23E-05	0.003339
ILMN_1776327	1	LOC727820		0.11	8.27E-05	0.003343
ILMN_1712916	4	MGC45800		0.04	8.27E-05	0.003343
ILMN_2099277	8	HTRA4		0.15	8.38E-05	0.003362
ILMN_1797950	1	EXTL2		-0.11	8.38E-05	0.003362
ILMN_1699226	1	UBR4		0.27	8.44E-05	0.003372
ILMN_1672124	4	C4orf18		-0.26	8.43E-05	0.003372
ILMN_1677756	5	UBE2D2		0.10	0.000085	0.003385
ILMN_1750225	6	MYCT1		-0.10	8.56E-05	0.0034
ILMN_1813796	2	TMEM169		0.09	8.58E-05	0.0034
ILMN_2409706	14	WDR20		-0.07	8.58E-05	0.0034
ILMN_1688953	10	ARHGAP19		-0.07	8.62E-05	0.003408
ILMN_1732127	2	RBKS		0.21	8.76E-05	0.003459
ILMN_2306630	7	SP8		0.05	8.78E-05	0.003459
ILMN_1651405	5	BRD9		0.11	8.89E-05	0.003498
ILMN_1653200	14	SLC22A17		0.20	8.95E-05	0.003515
ILMN_1806809	9	ZNF189		-0.21	8.98E-05	0.003517
ILMN_1802252	12	GAPDH		0.18	8.99E-05	0.003517
ILMN_1704014	1	LOC730256		0.13	9.02E-05	0.003522
ILMN_1660021	19	M6PRBP1		0.17	9.08E-05	0.003538
ILMN_3199974	1	LOC100131787		0.17	9.28E-05	0.003612
ILMN_1715173	1	RPS6KA1		0.14	9.33E-05	0.003612
ILMN_2204876	14	FLVCR2		0.12	9.34E-05	0.003612
ILMN_1797005	19	PGLS		0.11	9.33E-05	0.003612
ILMN_1715669	11	TP53I11		-0.09	9.36E-05	0.003612
ILMN_2174437	3	CIDEC		0.17	9.45E-05	0.003639
ILMN_3225784	14	LOC730029		0.13	0.000095	0.003652
ILMN_1708728	12	H2AFJ		0.16	9.53E-05	0.003657
ILMN_2336609	11	SYTL2		0.36	9.59E-05	0.003674
ILMN_1780268	1	C1orf56		-0.04	9.68E-05	0.003702
ILMN_1682428	1	C1orf59		0.14	9.84E-05	0.003742
ILMN_1775432	1	CSMD2		-0.06	9.83E-05	0.003742
ILMN_1798992	3	MYL3		-0.15	0.0001	0.003798
ILMN_1694041	9	PDCL		-0.08	0.0001	0.003798
ILMN_1688848	3	TMEM44		0.23	0.0001	0.0038
ILMN_2171384	4	CXCL5		0.18	0.000102	0.003857
ILMN_1715789	10	DOCK1		-0.11	0.000102	0.003857
ILMN_1703110	2	KCNE4		-0.06	0.000103	0.00387
ILMN_2181469	19	ZNF611		0.06	0.000103	0.00387
ILMN_1802131	7	SLC13A1		-0.26	0.000104	0.003893
ILMN_2106227	9	KIAA2026		-0.08	0.000104	0.003903
ILMN_1802441	2	REG1A		0.61	0.000105	0.003908
ILMN_1675684	22	APOBEC3C		0.08	0.000106	0.003942
ILMN_1707493	1	SNHG3-RCC1		0.12	0.000107	0.003986
ILMN_1704383	17	TRIM37		-0.08	0.000107	0.003986
ILMN_2113470	6	GSTA1		-0.57	0.000108	0.00399
ILMN_1712798	5	ZNF608		0.10	0.000108	0.00399
ILMN_1709740	14	TXNDC16		-0.06	0.000108	0.00399
ILMN_1747281	19	EVI5L		-0.13	0.000108	0.004002
ILMN_1725707	2	ATG16L1		0.22	0.000109	0.00401
ILMN_2225608	6	FAM135A		-0.10	0.000109	0.004019
ILMN_3208973	9	LOC653162		0.10	0.00011	0.00402
ILMN_1697773	8	ZNF517		-0.04	0.00011	0.004023
ILMN_1676197	6	LRP11		-0.12	0.000111	0.004047
ILMN_1751431	12	WIBG		0.08	0.000111	0.004047
ILMN_1657288	6	GUSBL2		0.07	0.000111	0.004051
ILMN_1787461	1	RUNX3		0.19	0.000112	0.004072
ILMN_2189027	18	LIPG		-0.22	0.000113	0.004083
ILMN_3263694	9	LOC100128771		0.14	0.000113	0.004083
ILMN_1713174	11	TCP11L1		0.08	0.000113	0.004083
ILMN_1684520	9	TSTD2		-0.03	0.000114	0.004115
ILMN_1707872	10	RUFY2		-0.03	0.000116	0.004173
ILMN_1810274	17	HOXB2		-0.19	0.000119	0.004272
ILMN_1696839	1	RPS27		0.15	0.00012	0.004285
ILMN_1789007	19	APOC1		0.39	0.000121	0.004338
ILMN_1684368	5	C5orf62		0.20	0.000122	0.004352
ILMN_1654109	5	EGFLAM		0.25	0.000123	0.004383
ILMN_3200384	4	LOC441013		0.13	0.000124	0.004416
ILMN_2413259	14	SOCS4		-0.11	0.000124	0.004416
ILMN_1747577	9	ALAD		-0.11	0.000127	0.004513
ILMN_2136133	8	PABPC1		0.14	0.000128	0.004543
ILMN_1730229	15	CGNL1		0.30	0.000129	0.004563
ILMN_1785284	14	ALDH6A1		-0.23	0.00013	0.004563
ILMN_1781472	1	CDC42BPA		-0.09	0.000129	0.004563
ILMN_2128741	14	RDH11		-0.11	0.000131	0.004597
ILMN_3235188	1	LOC100131187		-0.13	0.000132	0.00461
ILMN_1777453	17	LOC653419		-0.09	0.000132	0.00461
ILMN_1805228	19	LRG1		0.18	0.000133	0.00463
ILMN_2191720	19	ZNF471		-0.13	0.000133	0.004645
ILMN_1806754	9	GLDC		0.26	0.000135	0.004695
ILMN_1753249	11	DDX10		-0.15	0.000136	0.004713
ILMN_1663227	19	LOC646750		-0.10	0.000136	0.004713
ILMN_1711909	20	EDEM2		0.10	0.000137	0.004722
ILMN_1685625	11	UCP2		0.22	0.000138	0.004754
ILMN_2140207	15	ATPBD4		-0.10	0.000139	0.004774
ILMN_1685834	7	AMPH		-0.05	0.000139	0.004774
ILMN_1796130	6	LOC221710		-0.05	0.000141	0.004841
ILMN_1778836	2	SFRS7		-0.12	0.000144	0.004928
ILMN_1777397	4	MSX1		-0.17	0.000147	0.005021
ILMN_1729368	10	FZD8		-0.13	0.000148	0.005027

ILMN_1699011	2	LOC648682		0.09	0.000149	0.005055
ILMN_1736242	16	PLEKHG4		0.16	0.000149	0.005058
ILMN_1716658	19	ZNF543		-0.05	0.00015	0.00509
ILMN_2381841	16	DDX19B		0.09	0.000152	0.005151
ILMN_1786426	19	TMEM149		0.14	0.000154	0.005193
ILMN_1748291	1	C1orf55		-0.10	0.000154	0.005201
ILMN_2391264	19	DMKN		0.20	0.000156	0.005231
ILMN_1677466	12	DUSP6		-0.12	0.000156	0.005231
ILMN_1774387	20	ZHX3		0.12	0.000156	0.005231
ILMN_1769382	11	KBTBD3		-0.08	0.000155	0.005231
ILMN_1660357	7	LOC641825		0.08	0.000156	0.005231
ILMN_1789361	10	PKD2L1		0.13	0.000158	0.005259
ILMN_1694634	7	MOGAT3		0.08	0.00016	0.005301
ILMN_1655206	9	ZBTB34		-0.06	0.000159	0.005301
ILMN_1673953	3	HRH1		0.05	0.000164	0.005426
ILMN_1753482	11	FAM86C		-0.06	0.000164	0.005435
ILMN_2374244	12	DYRK2		0.19	0.000167	0.005472
ILMN_1718907	18	TSHZ1		-0.15	0.000166	0.005472
ILMN_2093674	14	GMFB		-0.12	0.000167	0.005472
ILMN_2093864	19	ZNF571		-0.05	0.000167	0.005472
ILMN_1806030	16	PPL		0.10	0.00017	0.005542
ILMN_3245829	9	AQP7P1		-0.09	0.000169	0.005542
ILMN_1733157	13	THSD1		-0.06	0.000169	0.005542
ILMN_1698441	10	MYST4		-0.05	0.00017	0.005542
ILMN_1777477	9	C9orf106		0.05	0.00017	0.005547
ILMN_1678919	1	YOD1		-0.10	0.000172	0.005604
ILMN_1671600	19	EPS8L1		0.11	0.000175	0.005672
ILMN_2197030	14	ZFYVE21		-0.12	0.000175	0.005676
ILMN_2098616	5	C5orf39		0.17	0.000176	0.005678
ILMN_2158242	10	SHOC2		-0.10	0.000177	0.005714
ILMN_1805636	17	PGAP3		-0.10	0.000177	0.005714
ILMN_2376859	11	PDGFD		-0.22	0.000181	0.005809
ILMN_3251132	15	TMOD2		-0.10	0.000181	0.005809
ILMN_3268403	19	LOC100128252		-0.17	0.000182	0.005824
ILMN_1797793	19	BLVRB		0.14	0.000182	0.005824
ILMN_1763265	18	CHMP1B		-0.13	0.000182	0.005824
ILMN_1660837	4	CLCN3		-0.11	0.000184	0.005852
ILMN_1700257	4	C4orf32		-0.13	0.000185	0.005882
ILMN_1758090	10	BCCIP		-0.06	0.000185	0.005882
ILMN_1714882	2	LOC375190		0.03	0.000186	0.005885
ILMN_1737164	14	TM9SF1		-0.09	0.000187	0.005928
ILMN_1680814	1	EDN2		0.19	0.000188	0.005943
ILMN_1800008	11	ACAT1		-0.26	0.00019	0.005985
ILMN_1768393	18	SNRPD1		-0.06	0.000191	0.006027
ILMN_1652787	10	PIK3AP1		0.12	0.000194	0.006087
ILMN_1739054	2	CCDC121		-0.08	0.000194	0.006087
ILMN_2184109	4	SLC34A2		0.16	0.000196	0.00613
ILMN_1697448	1	TXNIP		-0.16	0.000198	0.006162
ILMN_1662964	11	PRMT3		-0.10	0.000198	0.006162
ILMN_3253857	12	LOC100127971		0.04	0.0002	0.006204
ILMN_1769032	11	GLYATL1		-0.16	0.000201	0.00624
ILMN_1700159	9	NIPSNAP3A		-0.09	0.000201	0.00624
ILMN_1768004	10	PDCD4		-0.08	0.000202	0.006256
ILMN_1684694	8	ANK1		0.07	0.000204	0.006313
ILMN_1675273	22	PPIL2		-0.03	0.000206	0.006351
ILMN_1667224	1	SLAMF8		0.18	0.000206	0.006352
ILMN_1767503	15	GABRB3		-0.12	0.000208	0.006383
ILMN_3251145	11	LDHA		0.11	0.000207	0.006383
ILMN_1810785	1	RNF11		-0.10	0.000209	0.006389
ILMN_1673360	4	BDH2		-0.08	0.000208	0.006389
ILMN_2230117	20	AVP		0.09	0.000209	0.006403
ILMN_1788394	17	TTLL6		0.14	0.00021	0.006429
ILMN_1718766	16	MT1F		0.36	0.000211	0.006437
ILMN_1803825	10	CXCL12		-0.21	0.000211	0.006437
ILMN_1801091	2	CERKL		-0.07	0.000215	0.006552
ILMN_1791679	2	DNER		0.15	0.000216	0.006558
ILMN_3187771	14	C14orf167		-0.14	0.000217	0.006574
ILMN_1800091	3	RARRES1		0.32	0.000219	0.006648
ILMN_2228180	8	MSRA		-0.23	0.00022	0.006657
ILMN_1711516	3	ATP6V1A		-0.19	0.000221	0.006671
ILMN_1697597	1	KIAA0494		-0.12	0.000221	0.006671
ILMN_1673320	18	C18orf34		-0.11	0.000225	0.006749
ILMN_1783621	2	CMPK2		-0.04	0.000224	0.006749
ILMN_3239861	10	C10orf140		-0.11	0.000225	0.006763
ILMN_1727589	19	SULT2B1		0.07	0.000227	0.006795
ILMN_3251379	19	SLC35E1		0.08	0.000228	0.006803
ILMN_1671404	10	SVIL		-0.17	0.000229	0.006837
ILMN_1795286	6	C6orf47		-0.08	0.000229	0.006837
ILMN_1790951	19	C19orf50		0.07	0.000234	0.006972
ILMN_1692199	2	RNF103		-0.09	0.000235	0.006981
ILMN_1786823	17	ICAM2		-0.19	0.000235	0.006982
ILMN_1763878	13	CDADC1		-0.03	0.000237	0.007013
ILMN_1679971	19	CCDC123		0.06	0.000243	0.007177
ILMN_1779448	2	EFHD1		-0.30	0.000245	0.007209
ILMN_1724495	2	SESTD1		-0.12	0.000245	0.007209
ILMN_3249501	1	ZNF697		-0.08	0.000247	0.00727
ILMN_3205656	1	LOC391075		0.17	0.000249	0.007301
ILMN_2191428	17	UBB		-0.11	0.000249	0.007301
ILMN_1805996	15	SIN3A		-0.09	0.000251	0.007349
ILMN_1755215	6	SAMD5		-0.10	0.000252	0.007375
ILMN_1683854	9	ZNF484		-0.06	0.000253	0.00739
ILMN_1771800	17	PRKCA		0.16	0.000257	0.007477
ILMN_1671046	11	HSPB2		0.17	0.000257	0.00748
ILMN_1786015	16	CTCF		-0.07	0.000263	0.007648
ILMN_3284584	12	LOC390345		0.14	0.000264	0.007666
ILMN_1743538	10	MLLT10		-0.08	0.000264	0.007667
ILMN_1711627	16	SIAH1		-0.10	0.000265	0.007677
ILMN_1703174	13	FREM2		-0.12	0.000266	0.007683
ILMN_1692956	11	LOC643949		0.11	0.00027	0.007775
ILMN_1676759	20	DDX27		0.08	0.00027	0.007779
ILMN_1666385	19	CALM3		-0.12	0.000272	0.007833
ILMN_1800164	11	PPFIA1		0.09	0.000273	0.007833
ILMN_1779014	6	TSPYL1		-0.16	0.000274	0.007861
ILMN_1671374	19	C19orf53		0.11	0.000274	0.007861
ILMN_2052686	6	LCA5		-0.04	0.000275	0.007861
ILMN_1659517	14	WDR89		-0.03	0.000278	0.00794
ILMN_1814797	1	SLC35F3		0.12	0.000282	0.008045
ILMN_1671693	18	C18orf10		-0.10	0.000285	0.008102
ILMN_1738173	18	METTL4		-0.07	0.000289	0.008185
ILMN_1670912	2	ACVR2A		-0.05	0.00029	0.008221
ILMN_1780601	19	EMR1		0.07	0.000295	0.008343
ILMN_1695606	17	EFNB3		-0.09	0.000297	0.008364
ILMN_1665832	20	ID1		-0.08	0.000298	0.008364
ILMN_1730731	10	ERLIN1		-0.06	0.000298	0.008364
ILMN_1701655	12	SLC24A6		0.11	0.000299	0.008386
ILMN_2043306	2	EPB41L5		-0.08	0.0003	0.008396
ILMN_2205350	6	C6orf66		-0.06	0.0003	0.008396
ILMN_1710284	3	HES1		-0.16	0.000302	0.008433
ILMN_3226727	5	LOC729989		0.03	0.000303	0.00844
ILMN_1692261	3	EPHB1		-0.14	0.000305	0.008478
ILMN_1700034	4	C4orf6		0.07	0.000305	0.008478
ILMN_3242977	5	FLJ33630		-0.06	0.000306	0.008478
ILMN_2144088	8	FDFT1		-0.13	0.000307	0.0085
ILMN_1679587	18	LOC651202		0.16	0.000314	0.008682
ILMN_1802167	3	ALDH1L1		-0.33	0.000315	0.008691
ILMN_3251423	3	CHDH		0.20	0.000315	0.008691
ILMN_1710221	2	GPR35		0.11	0.000315	0.008691
ILMN_1710410	1	LOC730413		-0.10	0.000316	0.008708
ILMN_1763694	16	RSPRY1		0.14	0.00032	0.00878
ILMN_1655740	8	SNAI2		-0.10	0.000322	0.008831
ILMN_1654946	19	ZSCAN18		-0.17	0.000323	0.00884
ILMN_1690907	6	CCR6		0.19	0.000325	0.008872
ILMN_1777853	2	MBOAT2		-0.07	0.000324	0.008872
ILMN_1674782	2	LOC375295		0.29	0.000326	0.008886
ILMN_1800082	19	B3GNT3		0.08	0.000328	0.008936
ILMN_1681675	6	RBM16		-0.07	0.000328	0.008936
ILMN_1659544	11	STX3		-0.14	0.00033	0.008958
ILMN_1716465	1	RBP7		-0.23	0.000337	0.009111
ILMN_1668863	2	LYPD1		0.11	0.000337	0.009111
ILMN_1766675	5	CDH6		0.30	0.000338	0.009122
ILMN_1651568	3	MUC13		0.14	0.00034	0.009178
ILMN_1688818	10	LOC652864		-0.14	0.000341	0.009197
ILMN_1659029	22	FAM116B		0.12	0.000343	0.009218
ILMN_1706118	16	HN1L		0.11	0.000344	0.009254
ILMN_1713873	2	THSD7B		-0.10	0.000346	0.009263
ILMN_1723353	9	FAM122A		-0.08	0.000346	0.009263
ILMN_2293992	4	RAB28		-0.05	0.000346	0.009263
ILMN_2041190	5	F2RL1		0.22	0.000348	0.009264
ILMN_1659086	8	NEFL		0.22	0.000347	0.009264
ILMN_1658806	17	SLC5A10		-0.13	0.000348	0.009264
ILMN_1742473	10	C10orf72		-0.06	0.000348	0.009264
ILMN_1711289	19	GYS1		0.05	0.000348	0.009264
ILMN_2089329	13	SPRY2		-0.15	0.000351	0.009335
ILMN_1746831	19	ZNF114		0.12	0.000352	0.009348
ILMN_2229610	19	ZNF568		-0.03	0.000353	0.00936
ILMN_1807825	6	LY86		0.17	0.000357	0.009419
ILMN_1752269	20	ACSS1		-0.16	0.000356	0.009419
ILMN_1654441	10	OAT		-0.15	0.000357	0.009419
ILMN_1732468	4	HSPA4L		-0.11	0.000356	0.009419
ILMN_1804945	19	ZNF667		-0.07	0.000357	0.009419
ILMN_1705743	14	FLJ10357		-0.05	0.000358	0.009419
ILMN_1677043	1	AKR7A2		-0.17	0.000361	0.00949
ILMN_1728071	12	KRAS		-0.08	0.000364	0.009548
ILMN_1713449	12	TBX3		-0.08	0.000366	0.00958
ILMN_1730940	6	KLHDC3		-0.10	0.000371	0.009687
ILMN_3251501	15	SERF2		-0.10	0.000372	0.0097
ILMN_2413141	6	C6orf162		-0.05	0.000374	0.009749
ILMN_2121272	6	PDE10A		0.04	0.000377	0.009796
ILMN_1706957	10	BMPR1A		-0.08	0.000379	0.009847
ILMN_1652735	13	RFXAP		-0.04	0.000379	0.009847
ILMN_1681591	20	PTPN1		0.14	0.000381	0.009868
ILMN_1781416	10	FRAT1		-0.03	0.000382	0.009882
ILMN_1774432	20	DTD1		0.10	0.000383	0.009918
ILMN_1678957	5	WDR55		0.10	0.000387	0.009994
ILMN_1786353	4	CNGA1		-0.07	0.00039	0.010052
ILMN_2362974	10	CASP7		-0.05	0.000391	0.010075
ILMN_1672899	2	POMC		0.05	0.000393	0.010083
ILMN_1728662	11	ALDH3B1		0.18	0.000394	0.010101
ILMN_1676980	8	MTSS1		-0.12	0.000394	0.010101
ILMN_1736577	16	ZNF688		0.08	0.000396	0.010128
ILMN_1780444	10	ARL3		-0.08	0.000397	0.010141
ILMN_1778333	20	MMP24		0.12	0.000398	0.010146
ILMN_1705114	14	NUMB		-0.10	0.000398	0.010146
ILMN_2049063	13	KBTBD6		-0.04	0.0004	0.010185
ILMN_1660317	1	LEFTY1		0.12	0.000401	0.010203
ILMN_1651705	11	CAT		-0.18	0.000408	0.010366
ILMN_1717877	1	IVNS1ABP		-0.17	0.000409	0.010366
ILMN_1655414	19	TNFSF14		0.09	0.000409	0.010366
ILMN_1785795	4	METAP1		-0.09	0.000413	0.010454
ILMN_2235602	2	CCDC150		-0.07	0.000416	0.010489
ILMN_1805652	10	PPP2R2D		-0.07	0.000417	0.010508
ILMN_1764398	6	TUBE1		-0.06	0.000418	0.010536
ILMN_1729868	12	SPRYD4		-0.08	0.000421	0.010583
ILMN_1807596	9	UBAP1		-0.08	0.000422	0.010603
ILMN_3188110	19	C19orf60		0.10	0.000424	0.010631
ILMN_1788738	22	ZNRF3		-0.11	0.000426	0.010636
ILMN_1660709	1	LOC645682		0.07	0.000425	0.010636
ILMN_1813581	6	CNR1		0.05	0.000426	0.010636
ILMN_1654313	2	LOC730995		0.10	0.000429	0.010702
ILMN_1803652	9	C9orf91		0.08	0.000431	0.010729

ILMN_1714170	1	SPSB1		0.19	0.000435	0.010795
ILMN_1684802	10	TAF5		-0.06	0.000438	0.010847
ILMN_1786326	19	KCTD15		-0.08	0.000438	0.010855
ILMN_2086077	19	JUNB		-0.19	0.000442	0.010922
ILMN_1666175	1	IL22RA1		0.06	0.000442	0.010922
ILMN_1711331	1	MAP1LC3C		-0.03	0.000449	0.011061
ILMN_1654445	9	LOC347292		0.13	0.00045	0.011066
ILMN_2240597	20	TCEA2		-0.05	0.000449	0.011066
ILMN_1678745	8	NUDCD1		-0.04	0.000451	0.011071
ILMN_2407564	5	GPR98		-0.07	0.000454	0.011132
ILMN_1689473	8	ATP6V1H		-0.03	0.000459	0.011246
ILMN_1737972	8	TSPYL5		0.11	0.000462	0.01128
ILMN_1712894	19	FUT1		-0.04	0.000462	0.01128
ILMN_1709067	1	SAMD11		-0.11	0.000463	0.011286
ILMN_1776109	5	ZNF622		0.10	0.000465	0.011286
ILMN_1730504	6	AGPAT4		-0.09	0.000464	0.011286
ILMN_1693431	13	C13orf31		0.08	0.000465	0.011286
ILMN_1685856	8	FAM92A1		-0.07	0.000464	0.011286
ILMN_1722423	6	SHPRH		-0.06	0.000465	0.011286
ILMN_1800634	16	NME4		0.15	0.000466	0.011295
ILMN_1766171	17	SNF8		0.12	0.000468	0.01131
ILMN_1789059	1	GDAP2		-0.04	0.000467	0.01131
ILMN_2156982	2	IMP4		0.10	0.000469	0.011321
ILMN_1770850	14	PNMA1		-0.08	0.000471	0.011354
ILMN_3298400	10	LOC731954		-0.10	0.000473	0.011383
ILMN_1659914	9	C9orf98		0.04	0.000474	0.011401
ILMN_1670305	11	SERPING1		0.27	0.000477	0.011454
ILMN_1701613	11	RARRES3		0.26	0.000478	0.011454
ILMN_1677041	10	CHUK		-0.08	0.000478	0.011454
ILMN_1778136	17	ZMYND15		0.11	0.000479	0.011463
ILMN_1765044	10	CUTC		-0.09	0.00048	0.01148
ILMN_1795166	3	PTH1R		-0.28	0.000483	0.011522
ILMN_1685709	1	TMEM125		-0.19	0.000489	0.011657
ILMN_2334210	17	ITGB4		0.16	0.000493	0.011709
ILMN_1711878	4	ENOPH1		-0.10	0.000494	0.011733
ILMN_2141259	17	SPAG5		-0.06	0.000503	0.011924
ILMN_1813019	1	DNAJB4		-0.05	0.000503	0.011924
ILMN_1785891	14	PRKD1		-0.12	0.000507	0.011996
ILMN_1711383	20	STK4		0.13	0.000508	0.012012
ILMN_1812461	20	WISP2		0.11	0.00051	0.012049
ILMN_1685679	6	RNF146		-0.10	0.000522	0.012306
ILMN_1812273	9	EHMT1		-0.03	0.000525	0.012351
ILMN_1772703	8	OTUD6B		-0.06	0.000526	0.012364
ILMN_1758806	21	C21orf2		-0.10	0.00053	0.012422
ILMN_1751963	7	ZCWPW1		0.09	0.00053	0.012422
ILMN_1766245	17	SUPT4H1		-0.07	0.000529	0.012422
ILMN_2099594	1	SRP9		-0.11	0.000531	0.012445
ILMN_1661622	6	TBC1D7		0.12	0.000533	0.012479
ILMN_3181328	6	LOC100130179		0.39	0.000536	0.012521
ILMN_1697095	17	SUZ12P		-0.03	0.000536	0.012521
ILMN_1730032	2	BOK		0.12	0.000537	0.012531
ILMN_1700147	22	VPREB3		0.09	0.000539	0.012556
ILMN_1671337	1	SLC2A5		0.36	0.000541	0.01259
ILMN_2149766	17	APPBP2		-0.09	0.000555	0.012856
ILMN_1795128	13	C13orf23		-0.08	0.000556	0.012856
ILMN_2385373	14	DHRS4L1		-0.04	0.000556	0.012856
ILMN_1712475	16	HS3ST2		0.19	0.000557	0.012872
ILMN_1655485	1	C1orf124		-0.05	0.000559	0.012907
ILMN_1809537	3	MASP1		-0.15	0.000561	0.012921
ILMN_1709094	5	LIFR		-0.11	0.000564	0.012967
ILMN_1691143	10	RAB18		-0.08	0.000566	0.01301
ILMN_1793616	9	RNF38		-0.11	0.000569	0.013052
ILMN_1682264	17	DCAF7		-0.10	0.000569	0.013052
ILMN_1701195	6	PLA2G7		0.22	0.000574	0.01314
ILMN_3294222	6	LOC100132673		0.18	0.000576	0.01314
ILMN_2151056	10	C10orf32		-0.17	0.000576	0.01314
ILMN_1741300	18	ZNF407		-0.07	0.000575	0.01314
ILMN_1753500	10	ARHGAP12		-0.10	0.000577	0.013142
ILMN_1780504	19	C19orf26		0.04	0.000578	0.013142
ILMN_2151048	3	STAG1		-0.08	0.000582	0.013191
ILMN_1680643	14	KIAA1333		-0.05	0.000581	0.013191
ILMN_1681526	22	SLC5A1		-0.30	0.000586	0.013257
ILMN_1719449	4	DCLK2		-0.07	0.000586	0.013257
ILMN_1795704	4	KIAA0232		-0.07	0.000587	0.013276
ILMN_1813669	6	ANKS1A		-0.11	0.000588	0.013283
ILMN_1789830	2	CFLAR		0.16	0.000594	0.013389
ILMN_1809708	11	KCTD21		-0.03	0.000594	0.013389
ILMN_1687035	1	ADAMTSL4		0.06	0.000596	0.013407
ILMN_1683678	2	SPATS2L		-0.18	0.000598	0.013454
ILMN_1693471	11	KLHL35		0.10	0.000602	0.013508
ILMN_3237507	12	LOC552889		-0.08	0.000601	0.013508
ILMN_2340877	11	MEN1		-0.05	0.000608	0.013625
ILMN_1715508	11	NNMT		0.33	0.00061	0.01367
ILMN_1658526	3	RYK		-0.09	0.000614	0.013742
ILMN_1775956	2	SP5		0.07	0.000619	0.013833
ILMN_1703132	6	LYRM2		-0.13	0.000622	0.013887
ILMN_1653498	16	IGSF6		0.14	0.000623	0.013898
ILMN_2330243	7	NUDT1		0.12	0.000627	0.013965
ILMN_1673729	10	RHOBTB1		0.05	0.000633	0.014082
ILMN_1668194	19	LMTK3		0.19	0.000637	0.014153
ILMN_2367239	21	RCAN1		-0.25	0.00064	0.014197
ILMN_1705750	20	TGM2		0.26	0.000642	0.014216
ILMN_1892403	8	SNORD13		0.24	0.000641	0.014216
ILMN_1765668	3	IL20RB		0.37	0.000644	0.014227
ILMN_1716237	14	ACOT2		-0.11	0.000644	0.014227
ILMN_2133360	16	LOC91561		0.04	0.000644	0.014227
ILMN_1791232	2	SPRED2		-0.11	0.000645	0.014227
ILMN_1761594	10	FAM35A		-0.09	0.000651	0.014324
ILMN_1694752	10	LOC727726		-0.06	0.00065	0.014324
ILMN_2356890	12	MRPL42		-0.09	0.000653	0.014355
ILMN_1724941	3	CDCP1		0.19	0.000657	0.01443
ILMN_3248627	1	CHRM3		-0.08	0.000657	0.01443
ILMN_1700349	19	ADCK4		0.08	0.00066	0.014474
ILMN_1785005	22	NCF4		0.10	0.000661	0.014491
ILMN_1676003	8	PNOC		0.07	0.000662	0.014493
ILMN_1751607	19	FOSB		-0.44	0.000664	0.01451
ILMN_1652128	6	LMBRD1		-0.17	0.000665	0.01451
ILMN_1713162	1	GSTM2		-0.07	0.000668	0.014557
ILMN_2361400	2	ABCA12		0.06	0.00067	0.01457
ILMN_1765796	12	ENO2		0.26	0.000672	0.014605
ILMN_1685062	2	REG3G		0.23	0.000677	0.014699
ILMN_1721762	2	IL18RAP		0.09	0.000679	0.01472
ILMN_1787919	22	PARVB		0.14	0.000683	0.014804
ILMN_3291053	6	LOC346085		0.07	0.000687	0.014864
ILMN_1714228	2	SPTBN1		-0.03	0.000688	0.014871
ILMN_2231021	2	TMEM185B		-0.06	0.000689	0.014882
ILMN_3242105	2	LOC100134073		0.13	0.00069	0.014887
ILMN_1789596	12	ETV6		0.13	0.00069	0.014887
ILMN_1760688	17	SAMD14		-0.08	0.000692	0.014901
ILMN_1655341	5	PLAC8L1		0.04	0.000704	0.015127
ILMN_2173611	16	MT1E		0.38	0.000712	0.015241
ILMN_1705685	2	MEIS1		-0.09	0.000711	0.015241
ILMN_2320850	4	UBE2D3		-0.08	0.00071	0.015241
ILMN_1738420	1	TMEM201		-0.05	0.000711	0.015241
ILMN_3274671	13	LOC283481		0.10	0.000712	0.015243
ILMN_2161556	12	C12orf49		-0.10	0.000714	0.015249
ILMN_1804448	17	MSI2		-0.07	0.000714	0.015249
ILMN_2185665	2	PGAP1		-0.04	0.000715	0.015249
ILMN_1672094	2	DLX1		-0.05	0.000716	0.015253
ILMN_1729450	16	C16orf62		-0.06	0.000717	0.015263
ILMN_1765146	21	IFNAR2		0.10	0.000729	0.015499
ILMN_1736729	12	OAS2		-0.07	0.000729	0.015499
ILMN_1676423	6	CCNC		-0.10	0.000734	0.015566
ILMN_1722698	4	RCHY1		-0.06	0.000734	0.015566
ILMN_2212763	19	ICAM3		0.11	0.000737	0.015597
ILMN_1774334	5	HIGD2A		0.13	0.00074	0.015637
ILMN_1804929	3	OXTR		0.13	0.000743	0.015694
ILMN_1727740	6	SYNCRIP		-0.10	0.000745	0.015712
ILMN_1805344	17	DDX5		-0.11	0.000747	0.015745
ILMN_2129388	10	FAM190B		-0.10	0.00075	0.015785
ILMN_1752476	6	C6orf134		-0.03	0.000754	0.015851
ILMN_1690454	3	C3orf54		-0.13	0.000758	0.015919
ILMN_1728984	12	PA2G4		0.11	0.000764	0.016037
ILMN_2075927	1	STK40		-0.10	0.000766	0.016073
ILMN_1730082	11	RPUSD4		0.09	0.00077	0.016114
ILMN_1744647	12	CAND1		-0.08	0.00077	0.016114
ILMN_1775943	14	BRMS1L		-0.09	0.00078	0.016261
ILMN_1717799	2	PRKCE		-0.08	0.00078	0.016261
ILMN_3244240	7	LOC646476		0.07	0.000779	0.016261
ILMN_3246107	21	C21orf71		-0.04	0.000778	0.016261
ILMN_1698427	17	TMUB2		-0.03	0.000784	0.016323
ILMN_1755897	4	UGT2B7		0.33	0.000789	0.016413
ILMN_3245693	6	ADAT2		-0.03	0.000791	0.016437
ILMN_1720373	16	SLC7A5		0.28	0.000793	0.016457
ILMN_1703005	13	IFP38		0.08	0.000796	0.016484
ILMN_1751453	12	ABCC9		-0.04	0.000795	0.016484
ILMN_1775814	5	GHR		-0.20	0.000797	0.016492
ILMN_1815287	3	B3GALT3		-0.04	0.000805	0.016631
ILMN_2413527	10	VCL		-0.09	0.000814	0.016797
ILMN_3247261	4	RAPGEF2		-0.12	0.000826	0.016979
ILMN_1793241	5	SRD5A1		0.08	0.000825	0.016979
ILMN_1674580	5	TRIM36		0.07	0.000827	0.016987
ILMN_1723212	6	SFRS3		-0.10	0.00083	0.017018
ILMN_2203987	6	LACE1		-0.04	0.00083	0.017018
ILMN_2065273	5	NPY6R		-0.11	0.000839	0.017164
ILMN_1691112	18	PIGN		-0.09	0.000841	0.017189
ILMN_2352295	11	PRDM10		-0.03	0.000848	0.017308
ILMN_1781700	2	IL18R1		0.11	0.000851	0.017357
ILMN_3240765	11	ANO3		-0.06	0.000854	0.017388
ILMN_1677113	6	RNF8		-0.06	0.000859	0.017479
ILMN_1659923	9	GNAQ		-0.10	0.000862	0.017528
ILMN_1724907	6	NUDT3		-0.08	0.000865	0.017565
ILMN_2326712	21	BACE2		0.25	0.00087	0.017649
ILMN_1718924	15	ETFA		-0.15	0.000878	0.01776
ILMN_1679268	2	PELI1		-0.11	0.000878	0.01776
ILMN_3178406	2	KLHL29		0.10	0.00088	0.017786
ILMN_1793572	21	C21orf81		0.10	0.000881	0.017788
ILMN_2374687	4	PTPN13		-0.11	0.000885	0.017827
ILMN_2078975	7	GRM3		-0.06	0.000894	0.017971
ILMN_1748812	7	TMEM130		0.21	0.000897	0.017992
ILMN_2342579	5	IL7R		0.18	0.000897	0.017992
ILMN_1815556	10	PRAP1		0.14	0.00091	0.018169
ILMN_1760792	7	KLHL7		-0.09	0.00091	0.018169
ILMN_3201643	16	LOC100133019		0.15	0.000911	0.018181
ILMN_2192072	11	MMP7		0.48	0.000918	0.018277
ILMN_1779347	9	GARNL3		-0.06	0.000919	0.018277
ILMN_2358074	7	MAD1L1		0.05	0.000919	0.018277
ILMN_1689831	5	FLJ11235		-0.11	0.00092	0.018288
ILMN_3236551	10	WDFY4		0.04	0.000921	0.018298
ILMN_1797693	12	BRI3BP		0.09	0.000923	0.018309
ILMN_2415421	1	SLC30A2		0.16	0.000929	0.018419
ILMN_1728256	17	SPAG9		-0.07	0.000931	0.01843
ILMN_1760617	3	IFT80		-0.05	0.000934	0.018482
ILMN_2162253	4	NMU		0.09	0.000939	0.01856
ILMN_1814917	19	TLE2		-0.13	0.000946	0.018667
ILMN_1698258	1	DNAJC8		-0.07	0.000947	0.018667
ILMN_1681301	1	AIM2		0.16	0.000948	0.018683
ILMN_1708081	2	LCLAT1		-0.10	0.00095	0.018687
ILMN_3252936	3	KRT18P17		0.07	0.00095	0.018687
ILMN_1654497	10	ATAD1		-0.12	0.000953	0.018729

ILMN_3242676	12	TMEM233		-0.05	0.000956	0.018755
ILMN_1769720	8	STAU2		-0.08	0.000957	0.018764
ILMN_1728710	19	ZNF816A		0.09	0.000974	0.019054
ILMN_1714401	1	SNX27		-0.09	0.000974	0.019054
ILMN_1721758	6	ID4		-0.06	0.000974	0.019054
ILMN_1662640	20	C20orf127		0.22	0.000977	0.019072
ILMN_3224952	19	LOC729903		0.07	0.000978	0.019072
ILMN_1677226	22	ARVCF		-0.03	0.000977	0.019072
ILMN_2346727	8	MTUS1		-0.12	0.000979	0.019076
ILMN_2380237	17	C1QTNF1		0.27	0.000981	0.019101
ILMN_1720501	17	ABCA9		-0.04	0.000984	0.019128
ILMN_2376204	6	LTB		0.11	0.000986	0.019157
ILMN_1694778	5	LOC646723		0.22	0.00099	0.019207
ILMN_3245591	14	DIO3OS		0.09	0.000991	0.019218
ILMN_1786444	8	LPL		-0.15	0.001005	0.019447
ILMN_3179891	17	LOC100128007		0.04	0.001007	0.019479
ILMN_2318568	16	HCFC1R1		0.14	0.001009	0.019489
ILMN_1773337	10	DKK1		-0.08	0.001012	0.019511
ILMN_1748616	19	GTF2F1		0.07	0.001012	0.019511
ILMN_1671092	8	SOX17		-0.08	0.001022	0.019678
ILMN_1805726	12	LOC654069		0.07	0.001022	0.019678
ILMN_1801313	3	SIAH2		-0.07	0.001024	0.019687
ILMN_1699085	8	C8orf16		0.06	0.001024	0.019687
ILMN_1772798	15	ARPP19		-0.07	0.001031	0.019788
ILMN_1728512	22	YWHAH		-0.09	0.001034	0.019815
ILMN_2336781	6	SOD2		0.27	0.001041	0.019842
ILMN_1747771	4	LOC730744		-0.10	0.001039	0.019842
ILMN_1799604	4	OCIAD1		-0.10	0.001038	0.019842
ILMN_1766793	9	C9orf23		-0.05	0.001039	0.019842
ILMN_1781672	4	GAB1		-0.04	0.001041	0.019842
ILMN_1711799	9	C9orf40		-0.05	0.001044	0.019878
ILMN_2089616	9	FBXO10		-0.04	0.001046	0.019898
ILMN_1749868	10	FAM171A1		-0.15	0.001053	0.019986
ILMN_1669523	14	FOS		-0.36	0.001054	0.019995
ILMN_1658499	11	SYT13		0.37	0.001061	0.020081
ILMN_1765912	16	ACSM2B		-0.27	0.001062	0.020081
ILMN_1678680	3	DBR1		-0.05	0.001062	0.020081
ILMN_1728663	3	IL17RD		-0.03	0.00106	0.020081
ILMN_1689329	10	SCD		0.22	0.001065	0.020116
ILMN_1752631	3	CGGBP1		-0.11	0.001066	0.020123
ILMN_1767811	19	ZNF416		-0.05	0.001067	0.020128
ILMN_3245038	9	LOC100133972		-0.05	0.001071	0.020159
ILMN_1756928	14	RTN1		0.12	0.001076	0.02019
ILMN_3201221	12	LOC341315		0.10	0.001076	0.02019
ILMN_3240981	7	LOC100134587		0.09	0.001075	0.02019
ILMN_3277072	5	LOC728178		0.08	0.001074	0.02019
ILMN_3251269	15	SRP14		-0.12	0.001078	0.020199
ILMN_1792168	1	GALE		0.12	0.001085	0.020302
ILMN_1711023	20	CDK5RAP1		0.07	0.001087	0.020327
ILMN_1772359	1	LAPTM5		0.20	0.001099	0.020507
ILMN_1723625	11	MAP4K2		-0.11	0.001098	0.020507
ILMN_2093720	5	THG1L		0.08	0.0011	0.020507
ILMN_1803772	11	POLD4		0.08	0.001103	0.020555
ILMN_1769601	4	MGC16169		-0.07	0.001106	0.020599
ILMN_1733259	1	ADORA3		0.16	0.001114	0.0206
ILMN_1771120	11	TMEM45B		0.12	0.001114	0.0206
ILMN_1778964	6	CLIC5		-0.10	0.001109	0.0206
ILMN_1686405	4	KDR		-0.09	0.001115	0.0206
ILMN_1797031	3	HSPBAP1		0.08	0.00111	0.0206
ILMN_2401878	1	DUSP10		0.07	0.001114	0.0206
ILMN_2142752	12	MANSC1		-0.06	0.001108	0.0206
ILMN_1692107	11	SUV420H1		-0.04	0.001113	0.0206
ILMN_1760047	2	VAX2		-0.03	0.001111	0.0206
ILMN_1672947	5	CAST		0.09	0.001125	0.02077
ILMN_1685636	5	KCNN2		-0.05	0.001132	0.020858
ILMN_1800512	22	HMOX1		0.23	0.001138	0.020874
ILMN_1679133	6	SERPINB1		0.12	0.001137	0.020874
ILMN_1688630	9	RECK		-0.06	0.001138	0.020874
ILMN_2365484	15	SNX1		-0.06	0.001138	0.020874
ILMN_1758642	17	FLJ90757		-0.05	0.001137	0.020874
ILMN_1651950	7	TPST1		-0.12	0.001139	0.020875
ILMN_1783908	16	B3GNT9		-0.03	0.001142	0.020907
ILMN_1811489	3	OXSR1		-0.07	0.001144	0.020937
ILMN_1668507	1	DDAH1		-0.19	0.00115	0.021012
ILMN_1704208	1	LOC646981		0.05	0.001154	0.02107
ILMN_1813350	16	HSD11B2		-0.27	0.00116	0.021149
ILMN_1677384	1	PTGER3		-0.24	0.001159	0.021149
ILMN_2210934	4	NR3C2		-0.16	0.001173	0.021375
ILMN_1806533	6	PDE7B		-0.14	0.001182	0.021507
ILMN_1703305	3	TWF2		0.08	0.001185	0.021546
ILMN_1815634	20	COX4I2		-0.14	0.001187	0.021575
ILMN_2400663	7	AKAP9		0.04	0.001188	0.021575
ILMN_1796537	5	FYB		0.19	0.001202	0.021817
ILMN_3225406	10	LOC728640		0.13	0.001208	0.021865
ILMN_1807994	3	PCNP		-0.13	0.001207	0.021865
ILMN_2359601	10	CAMK2G		-0.04	0.001208	0.021865
ILMN_1734076	8	GPIHBP1		-0.04	0.001209	0.021865
ILMN_1754126	1	SH2D5		0.05	0.001211	0.021887
ILMN_1680018	21	RIPK4		0.12	0.001213	0.021899
ILMN_1657612	7	LOC285900		0.11	0.001214	0.0219
ILMN_1732176	9	AGPAT2		0.10	0.001215	0.021902
ILMN_2369682	7	HNRPA2B1		-0.09	0.001216	0.021908
ILMN_1804629	7	TPK1		0.14	0.001227	0.021909
ILMN_2188204	5	ATG12		0.13	0.001227	0.021909
ILMN_1763404	10	LOC653226		-0.11	0.001225	0.021909
ILMN_1695276	18	MAPRE2		0.10	0.001226	0.021909
ILMN_1793433	2	RAB10		-0.08	0.001227	0.021909
ILMN_2176768	10	SEPHS1		-0.08	0.001218	0.021909
ILMN_1804884	22	C22orf39		-0.06	0.001219	0.021909
ILMN_1792736	6	COL21A1		-0.04	0.001227	0.021909
ILMN_1699331	10	IFIT1		-0.04	0.001225	0.021909
ILMN_1712456	15	DKFZp434I1020		-0.03	0.001223	0.021909
ILMN_1681938	6	ALDH8A1		-0.12	0.001229	0.021918
ILMN_1787567	13	TSC22D1		-0.18	0.001234	0.021968
ILMN_1671818	1	UTS2		0.05	0.001236	0.021983
ILMN_1691175	4	ADH6		-0.05	0.001239	0.022019
ILMN_1771148	3	CNTN4		-0.04	0.001242	0.022037
ILMN_1734878	19	CD79A		0.19	0.001244	0.022055
ILMN_1746696	13	PDS5B		-0.07	0.001253	0.022204
ILMN_1713752	20	SERINC3		0.06	0.001257	0.022262
ILMN_2153547	11	OR4C15		-0.02	0.001268	0.02242
ILMN_1810275	14	SLC7A7		0.26	0.001273	0.022491
ILMN_1812327	8	RNF19A		0.11	0.001275	0.022504
ILMN_2408683	1	PPAP2B		-0.20	0.001279	0.022556
ILMN_1746664	12	WSB2		0.10	0.001282	0.022596
ILMN_1671925	5	SDHAP3		0.07	0.001284	0.02261
ILMN_1711087	8	LOC648526		0.14	0.001291	0.022722
ILMN_1779182	17	TMEM98		0.25	0.001293	0.022722
ILMN_1763837	15	ANPEP		0.28	0.001301	0.02286
ILMN_2409318	15	RCCD1		-0.05	0.001309	0.022971
ILMN_1774330	17	WSCD1		0.18	0.00131	0.022982
ILMN_1691860	4	SPRY1		-0.25	0.001316	0.023043
ILMN_1752658	17	NGFR		-0.06	0.001316	0.023043
ILMN_3307729	5	CXXC5		-0.13	0.001319	0.023078
ILMN_3235168	1	MUL1		-0.08	0.001321	0.023095
ILMN_1718182	17	THRA		-0.07	0.001326	0.023143
ILMN_1701636	16	LOC647786		-0.26	0.00133	0.023193
ILMN_2405305	11	ARNTL		-0.08	0.001331	0.023193
ILMN_1687074	9	CDC14B		-0.08	0.001333	0.023199
ILMN_1662910	10	SFXN3		-0.04	0.001333	0.023199
ILMN_3223126	22	TYMP		0.14	0.001343	0.023344
ILMN_3309016	19	SRRM5		-0.02	0.001347	0.02339
ILMN_1675377	15	ZSCAN2		-0.03	0.001353	0.023451
ILMN_1778595	11	SLN		0.21	0.001357	0.02347
ILMN_1757129	17	TMEM88		-0.16	0.001357	0.02347
ILMN_1761912	5	MGAT1		0.12	0.001357	0.02347
ILMN_2079655	12	KLRB1		0.19	0.00136	0.023497
ILMN_1801302	19	SCN1B		-0.08	0.001364	0.023538
ILMN_1760089	4	LOC645018		0.07	0.001365	0.023542
ILMN_1723287	19	SLC6A16		0.12	0.001373	0.023673
ILMN_2038776	9	TXN		-0.17	0.001376	0.023705
ILMN_2178226	12	KRT86		0.11	0.001383	0.023778
ILMN_3246538	1	LOC100133866		0.09	0.001387	0.023823
ILMN_1729188	19	HAMP		0.18	0.00139	0.023843
ILMN_3225761	1	MGC87895		0.14	0.00139	0.023843
ILMN_1673030	12	CMKLR1		0.05	0.001395	0.02389
ILMN_1681882	16	CETP		-0.05	0.001394	0.02389
ILMN_1781795	14	ERH		-0.11	0.0014	0.023943
ILMN_1760798	1	RYR2		0.06	0.001406	0.024024
ILMN_2120210	6	RCAN2		-0.25	0.001415	0.024138
ILMN_1667715	7	LOC645176		0.06	0.001415	0.024138
ILMN_1723048	1	GJC2		-0.15	0.001421	0.0242
ILMN_1767816	15	APH1B		0.09	0.001423	0.024208
ILMN_1676792	16	LOC645138		0.12	0.001426	0.024239
ILMN_3236061	7	ZNF783		0.09	0.00143	0.024282
ILMN_1795228	9	ZFAND5		-0.13	0.001435	0.024347
ILMN_1717078	1	DNAJC16		-0.03	0.001437	0.024367
ILMN_1715625	20	C20orf94		0.12	0.00144	0.024391
ILMN_1735075	1	PGBD2		-0.05	0.001441	0.024391
ILMN_3237956	11	ZC3H12C		-0.08	0.001443	0.024407
ILMN_1811735	8	C8orf22		0.08	0.001444	0.024408
ILMN_1792314	10	ACTR1A		-0.08	0.001445	0.02441
ILMN_1689552	1	FAM63A		0.15	0.001448	0.024435
ILMN_1761903	20	KCNS1		0.23	0.001457	0.024551
ILMN_2324998	14	NGDN		-0.11	0.00146	0.024594
ILMN_1689306	16	TMC7		-0.03	0.001462	0.024607
ILMN_2304404	10	PBLD		-0.14	0.001464	0.024618
ILMN_1700001	3	TCTA		-0.10	0.001467	0.02464
ILMN_2211739	3	SLC15A2		-0.04	0.001468	0.02464
ILMN_1749641	11	FBXO3		-0.12	0.001478	0.0248
ILMN_1787410	20	EIF6		0.09	0.001484	0.024857
ILMN_2290118	9	MEGF9		-0.07	0.001486	0.024874
ILMN_2376553	5	SLC23A1		0.06	0.001487	0.024878
ILMN_3289352	4	LOC642828		0.14	0.001492	0.024933
ILMN_2380946	11	EIF4G2		-0.08	0.001493	0.024933
ILMN_1689585	20	C20orf194		-0.06	0.001495	0.024951
ILMN_2397954	3	PARP3		0.13	0.001499	0.024992
ILMN_1738736	3	SNX4		-0.11	0.001503	0.025043
ILMN_2380740	6	MTO1		-0.09	0.001504	0.025043
ILMN_1672496	9	DNAJA1		-0.12	0.001511	0.025137
ILMN_1720996	5	SLC12A2		-0.12	0.00152	0.025268
ILMN_1669114	17	WNK4		0.11	0.001525	0.025341
ILMN_2413615	17	LLGL2		-0.04	0.001539	0.025542
ILMN_1675656	11	PPFIBP2		0.18	0.001541	0.025558
ILMN_3299478	1	LOC728428		0.11	0.001542	0.025567
ILMN_3233179	1	LOC728969		-0.10	0.001551	0.025692
ILMN_1732575	17	SEC14L1		-0.08	0.001555	0.025715
ILMN_1777745	7	FAM133B		-0.06	0.001555	0.025715
ILMN_1763539	18	IER3IP1		-0.10	0.001558	0.025747
ILMN_2353033	9	FUBP3		-0.12	0.001566	0.025819
ILMN_1715214	1	PTPN7		0.09	0.001565	0.025819
ILMN_1717262	20	PROCR		0.14	0.001568	0.025838
ILMN_1811779	9	MGC24103		0.18	0.00157	0.025844
ILMN_1704404	11	PSMD13		0.04	0.001571	0.025844
ILMN_3235853	1	S1PR1		-0.13	0.001572	0.025847
ILMN_1748983	2	RTN4		-0.20	0.001575	0.025852
ILMN_3246634	3	LOC100134108		0.12	0.00158	0.025852
ILMN_2297626	7	PEG10		-0.10	0.001577	0.025852
ILMN_1732921	7	ITGB8		0.07	0.001582	0.025852
ILMN_1740500	6	TSPYL4		-0.06	0.001578	0.025852

ILMN_3235803	9	LOC653458		-0.05	0.001582	0.025852
ILMN_1738407	6	ULBP1		-0.04	0.001581	0.025852
ILMN_2370588	22	TRIOBP		-0.03	0.001576	0.025852
ILMN_2392189	3	CTDSPL		-0.14	0.001584	0.025868
ILMN_1780699	16	THAP11		-0.08	0.001588	0.025898
ILMN_1691849	11	LIN7C		-0.04	0.001588	0.025898
ILMN_1801553	15	LEO1		-0.07	0.001594	0.025971
ILMN_3240843	1	LOC339535		0.28	0.001602	0.026012
ILMN_1777740	8	C8orf55		-0.12	0.001603	0.026012
ILMN_1783846	2	RAPH1		-0.11	0.001602	0.026012
ILMN_1723536	1	USP33		-0.06	0.001599	0.026012
ILMN_1656274	1	PRPF38A		-0.06	0.001601	0.026012
ILMN_2167011	6	ECHDC1		-0.08	0.001609	0.026028
ILMN_2117508	8	CTHRC1		0.18	0.001614	0.02608
ILMN_1669982	2	CCDC85A		-0.14	0.001615	0.026083
ILMN_1726769	18	CNDP2		0.15	0.001621	0.026092
ILMN_2196097	5	PPP2CA		-0.09	0.001621	0.026092
ILMN_1781098	10	DNAJB12		-0.04	0.001617	0.026092
ILMN_2350266	19	FBXO46		-0.04	0.001619	0.026092
ILMN_1674506	6	MED23		-0.05	0.001628	0.026182
ILMN_1705066	12	BTBD11		0.19	0.001633	0.026239
ILMN_1777318	9	C9orf64		0.10	0.001641	0.026343
ILMN_1692762	4	LOC649527		0.05	0.001645	0.026377
ILMN_1740505	10	SGMS1		-0.04	0.001644	0.026377
ILMN_1790625	7	CBX3		0.11	0.00165	0.026431
ILMN_3269324	17	FLJ37644		0.15	0.001661	0.026596
ILMN_1778890	14	PPIL5		-0.04	0.001668	0.026682
ILMN_1667476	12	LTBR		0.12	0.001685	0.026886
ILMN_1723141	10	OTUD1		-0.11	0.001685	0.026886
ILMN_1788347	14	KIAA1737		-0.10	0.001684	0.026886
ILMN_2067408	10	CLRN3		0.32	0.001693	0.026957
ILMN_1655549	19	SIGLEC10		0.15	0.001694	0.026957
ILMN_1728467	10	GHITM		-0.12	0.001691	0.026957
ILMN_1691156	16	MT1A		0.28	0.001703	0.027096
ILMN_1661189	1	ZBTB41		-0.04	0.001711	0.027197
ILMN_1791647	20	ASIP		0.14	0.001714	0.027203
ILMN_1786310	12	MVK		0.15	0.001716	0.02722
ILMN_2394305	22	PDGFB		-0.07	0.001717	0.02722
ILMN_2046003	10	ZDHHC6		-0.08	0.00172	0.027236
ILMN_3307651	22	APOBEC3D		0.10	0.001724	0.027289
ILMN_1737945	9	ALG2		-0.03	0.001733	0.0274
ILMN_1780798	7	FAM139A		0.03	0.001736	0.027442
ILMN_1790807	3	XPC		0.08	0.001742	0.027512
ILMN_1789387	11	KCNE3		-0.04	0.001746	0.027548
ILMN_3240092	11	MED17		-0.03	0.001748	0.027567
ILMN_1814022	11	NR1H3		0.13	0.001754	0.027636
ILMN_1795431	12	KLRA1		0.03	0.001756	0.027653
ILMN_1665923	1	THEM5		-0.02	0.001758	0.027661
ILMN_1887174	8	KIAA0146		-0.07	0.001765	0.027742
ILMN_1807067	20	C20orf118		0.06	0.001765	0.027742
ILMN_1804652	17	PLEKHH3		-0.10	0.00177	0.0278
ILMN_1796180	11	CRY2		-0.13	0.001778	0.027869
ILMN_1782129	22	ZBED4		-0.06	0.001778	0.027869
ILMN_1672504	21	PDXK		0.14	0.001806	0.028248
ILMN_1731619	14	DAD1		-0.13	0.001805	0.028248
ILMN_1717094	9	ZNF618		-0.07	0.001808	0.028248
ILMN_1738124	19	ZNF772		-0.03	0.001808	0.028248
ILMN_1793651	12	UBE2N		-0.09	0.00181	0.028253
ILMN_2198413	11	MYEOV		0.18	0.001817	0.028317
ILMN_1701461	22	TIMP3		-0.26	0.001819	0.028322
ILMN_1731914	17	SCN4A		-0.05	0.001823	0.028344
ILMN_1797375	13	KLF12		0.06	0.001825	0.02835
ILMN_1660462	1	MCOLN2		0.15	0.001829	0.028389
ILMN_1669516	6	ZNF193		-0.06	0.00183	0.028396
ILMN_3269484	2	LOC100128899		0.12	0.001832	0.028407
ILMN_1721978	7	CARD11		0.11	0.001839	0.028494
ILMN_1704795	14	MARK3		-0.04	0.001842	0.028514
ILMN_1673069	19	DPP9		0.15	0.001843	0.028517
ILMN_1893633	10	LOC439949		0.10	0.00185	0.028597
ILMN_1705570	10	H2AFY2		0.15	0.001853	0.028635
ILMN_3228595	1	LOC729768		0.13	0.001858	0.028681
ILMN_1795503	5	MCC		-0.03	0.001859	0.028681
ILMN_2188966	16	IGFALS		-0.03	0.001867	0.028765
ILMN_1811636	3	IFT57		-0.09	0.001876	0.028864
ILMN_1763941	15	LRRC49		-0.09	0.001879	0.028893
ILMN_1678541	20	ATRN		-0.03	0.001882	0.028914
ILMN_1763364	15	WHAMM		0.08	0.001893	0.029044
ILMN_1657679	1	VAV3		-0.15	0.001896	0.029064
ILMN_1744968	3	KCNAB1		-0.19	0.001897	0.029066
ILMN_1669631	4	GLRB		0.11	0.001899	0.029068
ILMN_1784287	1	TGFBR3		-0.15	0.001919	0.029283
ILMN_1761804	1	ALDH9A1		-0.11	0.001921	0.029283
ILMN_1790603	1	ATPAF1		-0.11	0.00192	0.029283
ILMN_1741477	18	SMAD4		-0.10	0.001919	0.029283
ILMN_1813374	19	C19orf28		0.09	0.001919	0.029283
ILMN_3243899	8	C8orf83		-0.09	0.001927	0.029303
ILMN_1663113	22	TTLL12		0.09	0.001928	0.029303
ILMN_1748968	5	ATG10		0.11	0.00195	0.029547
ILMN_1789383	6	LOC650976		-0.03	0.001952	0.029564
ILMN_2136068	6	SEC63		-0.04	0.00196	0.029664
ILMN_1752914	3	CDGAP		-0.07	0.001969	0.029768
ILMN_3251303	3	NR2C2		-0.04	0.00197	0.029768
ILMN_1792270	10	C10orf46		-0.03	0.001979	0.029886
ILMN_1687626	11	ZDHHC24		0.08	0.001994	0.030077
ILMN_1779032	17	LOC642502		-0.05	0.001995	0.030077
ILMN_2312275	14	SRP54		-0.09	0.002001	0.030147
ILMN_1803956	3	BOC		-0.04	0.002009	0.030249
ILMN_1813846	12	P2RX4		0.10	0.002011	0.030251
ILMN_1759801	15	DPP8		-0.06	0.002015	0.030251
ILMN_3239946	3	LOC729375		-0.05	0.002013	0.030251
ILMN_1786357	11	CENTD2		0.05	0.002016	0.030251
ILMN_1812552	11	PHCA		0.12	0.002017	0.030257
ILMN_1766054	9	ABCA1		0.15	0.002033	0.03042
ILMN_1750338	10	C10orf47		0.06	0.002034	0.03042
ILMN_2053546	5	LOC653314		0.09	0.002037	0.030421
ILMN_2351309	10	TIAL1		-0.09	0.002038	0.030421
ILMN_1745813	10	KIAA1279		-0.08	0.002037	0.030421
ILMN_1733248	8	NRBP2		0.16	0.002062	0.030719
ILMN_1763972	9	TJP2		-0.05	0.002061	0.030719
ILMN_3246273	14	RNU1-3		0.21	0.002065	0.030736
ILMN_1853824	22	MGAT3		0.21	0.002068	0.030771
ILMN_2305225	16	NDRG4		-0.12	0.002076	0.030857
ILMN_1678710	10	PHYHIPL		-0.14	0.002086	0.030934
ILMN_1696870	2	TGFBRAP1		0.09	0.002085	0.030934
ILMN_1682495	3	FOXP1		-0.08	0.002085	0.030934
ILMN_2186597	6	RPP21		-0.08	0.002088	0.030934
ILMN_1717973	21	TMEM1		0.04	0.002087	0.030934
ILMN_1712865	1	SHE		-0.05	0.00209	0.030951
ILMN_1778187	1	TARS2		0.06	0.002096	0.03101
ILMN_1666827	4	C4orf41		-0.06	0.002099	0.031028
ILMN_2234016	13	FTHL7		-0.15	0.002102	0.031044
ILMN_1779015	7	ZNF467		-0.13	0.002105	0.031046
ILMN_1758548	1	NEK7		-0.04	0.002104	0.031046
ILMN_1803788	14	LGALS3		0.18	0.00211	0.031102
ILMN_1662129	15	RCN2		-0.14	0.002132	0.031395
ILMN_1690252	12	ALKBH2		0.10	0.002139	0.031478
ILMN_1683859	13	SLC7A1		0.16	0.002141	0.031488
ILMN_1765729	11	MAML2		-0.04	0.002146	0.03152
ILMN_1794597	3	ZNF167		0.02	0.002146	0.03152
ILMN_1712684	7	FAM20C		0.16	0.002152	0.031586
ILMN_1769783	8	ZDHHC2		-0.06	0.002155	0.031606
ILMN_1758038	14	ALKBH1		-0.05	0.002158	0.03163
ILMN_1727390	17	ATPAF2		-0.04	0.002164	0.031696
ILMN_2049727	20	C20orf111		0.09	0.002166	0.031711
ILMN_1805930	1	CSF1		0.03	0.002181	0.031883
ILMN_1673604	6	YIPF3		-0.08	0.002188	0.031962
ILMN_1706990	18	ZNF271		-0.07	0.002196	0.032067
ILMN_1653687	12	GALNT9		-0.26	0.002216	0.032288
ILMN_1676946	8	AP3M2		-0.08	0.002218	0.032294
ILMN_2210519	5	HAPLN1		0.05	0.002219	0.032294
ILMN_1809259	11	HRASLS2		0.05	0.002229	0.032417
ILMN_1655654	17	MPDU1		0.09	0.002234	0.032446
ILMN_1703379	7	VPS41		0.08	0.002235	0.032446
ILMN_1768449	17	PRPSAP1		-0.07	0.002234	0.032446
ILMN_1763603	14	FAM177A1		-0.10	0.002246	0.03254
ILMN_2087629	18	NOL4		-0.07	0.002246	0.03254
ILMN_1690690	10	SEC23IP		-0.07	0.002251	0.032592
ILMN_3244176	11	LOC399959		0.15	0.002276	0.032901
ILMN_1676296	5	PPAP2A		-0.08	0.002277	0.032901
ILMN_1779480	2	KCMF1		-0.03	0.002283	0.032965
ILMN_2102752	4	LOC285453		0.02	0.002296	0.033112
ILMN_1700432	1	ITPKB		-0.10	0.002299	0.033127
ILMN_2380801	6	FYN		-0.09	0.0023	0.033127
ILMN_2407308	8	HEY1		-0.05	0.002307	0.033196
ILMN_1656427	17	C17orf39		-0.04	0.002309	0.033211
ILMN_1788377	9	COL27A1		-0.07	0.002311	0.033221
ILMN_1777765	12	C12orf10		0.09	0.002315	0.033244
ILMN_2168520	11	C11orf70		0.12	0.002318	0.03325
ILMN_1887403	1	DBT		-0.04	0.002318	0.03325
ILMN_1736863	7	TMEM140		0.14	0.002332	0.033414
ILMN_2082314	22	TOM1		0.11	0.002339	0.033465
ILMN_1803813	3	ASTE1		-0.05	0.002375	0.033931
ILMN_1736002	8	COPS5		-0.09	0.002379	0.033974
ILMN_1674983	12	LOC387841		0.12	0.00239	0.034024
ILMN_2360730	7	CAV2		0.11	0.002388	0.034024
ILMN_1758597	17	NAGS		0.04	0.002387	0.034024
ILMN_3197549	17	FLJ77644		-0.03	0.00239	0.034024
ILMN_2179726	16	C16orf93		0.10	0.002397	0.034093
ILMN_1747058	16	TRAPPC2L		0.11	0.002411	0.034275
ILMN_1733288	12	C1RL		0.11	0.002415	0.034307
ILMN_1705141	1	CACYBP		-0.03	0.002422	0.034379
ILMN_2366388	1	PRDX1		0.08	0.002424	0.034397
ILMN_1785010	13	TPTE2		0.03	0.002426	0.034404
ILMN_2145518	11	TMEM126B		-0.10	0.002431	0.034447
ILMN_3211079	1	LOC148430		0.08	0.002434	0.034469
ILMN_1690566	10	RASSF4		0.14	0.002442	0.034546
ILMN_1698885	20	PTPRT		-0.03	0.002443	0.034546
ILMN_1695299	4	PDLIM3		0.16	0.002451	0.034619
ILMN_1655702	3	ABHD5		-0.07	0.002455	0.034649
ILMN_3307733	17	C17orf42		-0.08	0.00246	0.034707
ILMN_1663257	8	ATP6V1C1		-0.03	0.002462	0.034708
ILMN_1797698	20	RBM12		-0.07	0.002464	0.034713
ILMN_1753207	19	FZR1		-0.03	0.002469	0.034756
ILMN_1696767	13	SERP2		-0.09	0.002481	0.034892
ILMN_1708147	6	TBPL1		-0.08	0.002484	0.034903
ILMN_1697000	9	KIAA0258		-0.05	0.002498	0.035084
ILMN_2364852	6	BTN2A1		-0.08	0.002509	0.035213
ILMN_1672605	7	C7orf41		-0.16	0.002515	0.035278
ILMN_1741997	6	SNRPC		-0.08	0.002527	0.035417
ILMN_2112357	17	CD300LF		0.10	0.002544	0.03563
ILMN_2322498	15	RORA		0.10	0.00256	0.035808
ILMN_1718852	2	PLCL1		-0.08	0.00257	0.035924
ILMN_2072140	1	BTF3L4		-0.09	0.002573	0.035952
ILMN_1701603	1	ALPL		-0.34	0.002579	0.036008
ILMN_2323526	10	WAC		-0.09	0.002581	0.036023
ILMN_1655312	11	HPS5		0.07	0.002598	0.036201
ILMN_1810855	15	C15orf58		0.06	0.002597	0.036201
ILMN_1728049	1	S100A16		-0.12	0.002605	0.036287
ILMN_1719209	17	FLJ31222		-0.13	0.002618	0.036399
ILMN_1743208	12	NEDD1		-0.04	0.002625	0.03645

ILMN_1714965	4	NFKB1		-0.08	0.002642	0.03664
ILMN_3244065	9	C9orf69		-0.08	0.002652	0.036727
ILMN_2365711	2	2-Sep		-0.09	0.002656	0.036741
ILMN_2330267	4	ABCE1		-0.08	0.002656	0.036741
ILMN_1789641	4	ABCG2		-0.09	0.002669	0.036874
ILMN_1716006	18	C18orf54		-0.04	0.00269	0.037143
ILMN_1658152	19	MAP3K10		-0.03	0.002697	0.037189
ILMN_1664006	1	AGL		-0.06	0.002701	0.037222
ILMN_1692824	17	ASPA		-0.17	0.002711	0.037328
ILMN_1758938	9	SLC31A2		0.14	0.002712	0.037328
ILMN_1760849	16	NETO2		0.21	0.002715	0.03734
ILMN_2197225	5	EIF4EBP3		0.09	0.002723	0.037431
ILMN_1694034	19	LGALS4		0.33	0.002726	0.037443
ILMN_1659761	16	SNX29		-0.10	0.00274	0.037594
ILMN_1678814	19	UBL5		-0.06	0.002739	0.037594
ILMN_1814686	16	BCMO1		0.16	0.002752	0.037693
ILMN_1667330	19	LOC113386		-0.07	0.002752	0.037693
ILMN_1661627	1	LZIC		-0.06	0.002757	0.037733
ILMN_1803194	17	GALK1		0.12	0.00277	0.037882
ILMN_1794825	17	ALDH3A2		-0.20	0.002774	0.037884
ILMN_1663437	16	NECAB2		-0.09	0.002772	0.037884
ILMN_1676215	11	DLG2		-0.05	0.002775	0.037884
ILMN_3251491	8	UQCRB		-0.04	0.002778	0.037907
ILMN_1812070	7	ABCB1		0.19	0.002795	0.038014
ILMN_1703593	7	BAIAP2L1		0.17	0.002796	0.038014
ILMN_1769757	14	C14orf104		-0.07	0.002798	0.038014
ILMN_1788832	18	MIB1		-0.05	0.002798	0.038014
ILMN_1724233	15	LOC145783		-0.04	0.002793	0.038014
ILMN_2371055	1	EFNA1		0.18	0.002808	0.03804
ILMN_1720303	6	OSTM1		-0.09	0.002809	0.03804
ILMN_2166524	2	CCNYL1		-0.06	0.002803	0.03804
ILMN_1666007	14	TRAPPC6B		-0.05	0.002808	0.03804
ILMN_1800713	21	B3GALT5		0.03	0.002805	0.03804
ILMN_1732612	9	SHB		-0.08	0.002817	0.038127
ILMN_1678805	14	POMT2		-0.07	0.00282	0.038127
ILMN_3180855	9	LOC100128077		0.05	0.00282	0.038127
ILMN_1659913	15	ISG20		0.16	0.002827	0.038157
ILMN_1754538	10	C10orf58		-0.15	0.002828	0.038157
ILMN_1731891	8	UBXN8		-0.07	0.002827	0.038157
ILMN_1741264	7	MRPS33		0.12	0.002836	0.038243
ILMN_1695290	14	FERMT2		-0.13	0.002846	0.038309
ILMN_1669878	7	GUSB		0.08	0.002845	0.038309
ILMN_1799026	4	PCDH18		-0.09	0.002862	0.038495
ILMN_3245678	14	RNU1A3		0.16	0.002867	0.038526
ILMN_1692295	7	MYO1G		0.12	0.002885	0.038691
ILMN_1674795	12	FLJ33996		0.04	0.002884	0.038691
ILMN_1714700	2	TRIB2		-0.16	0.002918	0.039041
ILMN_2057573	7	FAM62B		0.09	0.002918	0.039041
ILMN_3226641	11	PLA2G16		0.08	0.002916	0.039041
ILMN_1677877	22	UBE2L3		-0.09	0.002923	0.039048
ILMN_1811363	14	NOVA1		-0.06	0.002922	0.039048
ILMN_1652777	11	CDC42EP2		-0.05	0.002924	0.039048
ILMN_1735275	2	WDSUB1		-0.09	0.002929	0.039095
ILMN_3243156	14	AHNAK2		0.27	0.002937	0.039175
ILMN_1760303	5	PIK3R1		0.14	0.002956	0.039383
ILMN_1724533	8	LY96		0.21	0.002965	0.039476
ILMN_1810229	14	ARID4A		-0.05	0.002971	0.039527
ILMN_1653828	12	CHFR		0.09	0.002973	0.039536
ILMN_1715991	2	SDPR		-0.21	0.00298	0.039612
ILMN_1730977	6	SLC44A4		0.15	0.00299	0.039661
ILMN_1701512	14	KIAA0391		-0.08	0.002988	0.039661
ILMN_1712560	11	SF1		-0.04	0.002991	0.039661
ILMN_2172174	14	NP		-0.11	0.002993	0.039661
ILMN_1682494	3	RSRC1		-0.08	0.003012	0.039841
ILMN_1763460	22	NHP2L1		-0.07	0.00301	0.039841
ILMN_2356632	2	NCK2		-0.05	0.003012	0.039841
ILMN_1674038	11	CTSD		0.19	0.003021	0.039897
ILMN_2166093	11	PIK3C2A		-0.06	0.003025	0.039897
ILMN_1703006	6	MTCH1		-0.07	0.003033	0.039972
ILMN_1798081	1	PTPRF		0.13	0.003041	0.04005
ILMN_3261226	6	C6orf186		-0.03	0.003048	0.040081
ILMN_1723843	16	CSNK2A2		0.08	0.003058	0.04018
ILMN_1800414	1	NBPF20		0.04	0.003064	0.040239
ILMN_1740728	17	SMTNL2		-0.10	0.003079	0.040415
ILMN_1711102	2	B3GNT2		-0.09	0.00309	0.040507
ILMN_1746618	1	PAQR7		0.12	0.003105	0.040683
ILMN_2066849	6	FAM26F		0.08	0.003111	0.040737
ILMN_2361603	14	NDRG2		-0.18	0.003125	0.040891
ILMN_2382354	3	SENP7		-0.09	0.003128	0.040903
ILMN_1778755	7	TRIL		-0.13	0.003135	0.040972
ILMN_1808508	12	KITLG		-0.13	0.00314	0.041009
ILMN_2083066	22	IGLL3		0.21	0.003152	0.041129
ILMN_1722642	10	REEP3		-0.04	0.003152	0.041129
ILMN_1799598	6	SIRT5		-0.06	0.003157	0.04114
ILMN_2195957	8	RHOBTB2		-0.05	0.003158	0.04114
ILMN_2340131	4	MAPK10		-0.04	0.003159	0.04114
ILMN_1727309	15	FAM82A2		-0.08	0.003162	0.041151
ILMN_3238676	6	ULBP2		-0.04	0.003196	0.04152
ILMN_1750394	18	SLC39A6		-0.08	0.003202	0.04158
ILMN_1728478	17	CXCL16		0.15	0.003207	0.041585
ILMN_1673129	5	PCDH12		-0.14	0.003208	0.041585
ILMN_1765825	14	WDR23		-0.07	0.003205	0.041585
ILMN_1655924	3	TRNT1		-0.07	0.003211	0.041593
ILMN_2172969	14	STXBP6		0.21	0.003219	0.041673
ILMN_2079004	7	MDH2		0.08	0.003235	0.04183
ILMN_1787345	12	FKBP11		0.18	0.003239	0.041857
ILMN_1713454	19	ZNF671		-0.08	0.003249	0.04194
ILMN_3265365	9	CEP78		-0.05	0.003259	0.042041
ILMN_1751887	6	PREP		-0.06	0.003266	0.042063
ILMN_1695947	11	SCN4B		-0.13	0.00327	0.042085
ILMN_1811820	12	SRRM4		-0.06	0.003274	0.042123
ILMN_1710280	20	TUBB1		-0.03	0.003277	0.04213
ILMN_3178307	1	KRT18P28		0.05	0.003281	0.042158
ILMN_2393994	8	CSPP1		-0.07	0.003289	0.042216
ILMN_1669584	19	ILF3		-0.07	0.003288	0.042216
ILMN_1767219	6	POLR1C		-0.07	0.003295	0.042259
ILMN_1797822	4	SEL1L3		0.18	0.003304	0.042318
ILMN_1730201	18	DTNA		0.10	0.003305	0.042318
ILMN_1783266	7	OGDH		-0.04	0.003304	0.042318
ILMN_1738632	17	PRKAR1A		-0.16	0.003307	0.042319
ILMN_1770244	17	CBX1		-0.08	0.003311	0.042332
ILMN_1772692	14	DICER1		-0.07	0.00332	0.04236
ILMN_1672356	11	ANKRD13D		0.07	0.00332	0.04236
ILMN_1719468	6	EPM2A		-0.04	0.003317	0.04236
ILMN_1754839	4	DHX15		-0.09	0.003324	0.042369
ILMN_3239789	14	YLPM1		-0.03	0.003344	0.042602
ILMN_2399363	12	CLEC4A		0.11	0.003347	0.042602
ILMN_1710092	20	ZBTB46		-0.10	0.003348	0.042602
ILMN_2117904	10	ZNF22		-0.10	0.003357	0.042686
ILMN_3229324	6	SGK1		-0.20	0.003366	0.042778
ILMN_1741440	6	SLC35A1		-0.09	0.003374	0.042804
ILMN_2374778	15	DUT		-0.06	0.003372	0.042804
ILMN_1678831	2	GCC2		0.06	0.003373	0.042804
ILMN_1781761	6	ENPP4		-0.12	0.003377	0.042817
ILMN_1811754	16	NDUFB10		0.09	0.00339	0.042953
ILMN_3202109	15	LOC400446		-0.03	0.003392	0.042957
ILMN_2239754	10	IFIT3		-0.11	0.0034	0.043011
ILMN_1741176	3	CHMP2B		-0.10	0.003399	0.043011
ILMN_1662839	10	PLEKHA1		-0.13	0.003404	0.043036
ILMN_2342835	3	P2RY14		-0.04	0.003407	0.043045
ILMN_1758487	7	CNPY4		-0.04	0.003414	0.043088
ILMN_2061043	1	CD48		0.16	0.00342	0.043132
ILMN_2246882	2	SP140		0.07	0.003421	0.043132
ILMN_1763523	5	HARS		0.09	0.003426	0.043137
ILMN_1807515	10	CSTF2T		-0.05	0.003427	0.043137
ILMN_1674608	15	FAM169B		0.08	0.00343	0.043145
ILMN_1698246	1	MTMR11		0.07	0.003436	0.04317
ILMN_1687544	19	ZNF567		-0.03	0.003436	0.04317
ILMN_1696962	10	PDZD8		-0.06	0.003442	0.043174
ILMN_2284706	13	PHF11		-0.03	0.003442	0.043174
ILMN_2405470	1	KLHDC9		-0.08	0.003446	0.043194
ILMN_1813530	1	AGT		0.22	0.003455	0.043288
ILMN_1667460	20	SULF2		0.17	0.00347	0.043367
ILMN_3269395	22	GGT2		-0.08	0.003471	0.043367
ILMN_3251572	2	FAM134A		-0.08	0.003478	0.043401
ILMN_1758213	10	GPAM		-0.07	0.003492	0.04351
ILMN_1694966	17	ASGR2		0.04	0.003491	0.04351
ILMN_1774990	16	C16orf13		0.12	0.003504	0.043557
ILMN_1664231	10	TIMM23		-0.10	0.003501	0.043557
ILMN_1701248	6	TREM2		0.09	0.003504	0.043557
ILMN_1744244	11	DPAGT1		-0.04	0.003507	0.043565
ILMN_1749667	1	OBSCN		0.06	0.003512	0.043597
ILMN_1779501	18	MC5R		-0.07	0.003515	0.043613
ILMN_2044293	13	KBTBD7		-0.05	0.003539	0.043883
ILMN_1762231	10	ZEB1		-0.04	0.003553	0.044012
ILMN_1677404	13	RAP2A		-0.11	0.003573	0.044236
ILMN_1753627	9	DOLK		-0.06	0.003583	0.04433
ILMN_2411127	21	C21orf57		0.09	0.003585	0.044338
ILMN_1701403	7	HIP1		-0.07	0.003595	0.044438
ILMN_1680239	4	NUDT9		-0.08	0.003599	0.044461
ILMN_1727080	6	MYO6		-0.08	0.003607	0.044528
ILMN_1656837	3	RBP1		-0.14	0.003631	0.044756
ILMN_1733535	5	ZNF366		-0.07	0.003631	0.044756
ILMN_1767349	7	ABCB4		0.08	0.003634	0.044767
ILMN_1793435	1	KIAA1107		-0.04	0.003639	0.044779
ILMN_1688071	8	NAT1		-0.06	0.003643	0.044784
ILMN_3249406	1	URB2		-0.05	0.003644	0.044784
ILMN_1775448	3	PFN2		-0.19	0.003665	0.04483
ILMN_1752793	13	SAP18		-0.10	0.00366	0.04483
ILMN_2289924	3	TRAK1		-0.09	0.003656	0.04483
ILMN_2414268	2	COLEC11		-0.08	0.003656	0.04483
ILMN_1700248	7	WDR86		-0.08	0.003661	0.04483
ILMN_3247438	19	C19orf69		-0.06	0.003666	0.04483
ILMN_1728228	19	ZNF548		-0.05	0.003662	0.04483
ILMN_3236289	3	TIGIT		0.02	0.003661	0.04483
ILMN_1787199	12	C12orf28		-0.09	0.003697	0.045146
ILMN_1774161	5	ARL15		-0.06	0.003698	0.045146
ILMN_3236169	7	MACC1		0.03	0.003702	0.045177
ILMN_1792182	14	RDH12		-0.07	0.003708	0.045223
ILMN_1762071	17	C17orf80		-0.05	0.003712	0.045241
ILMN_2404917	10	AFAP1L2		-0.08	0.003714	0.045243
ILMN_3248786	1	LOC647588		0.03	0.003716	0.04525
ILMN_3230337	7	LOC100132707		0.09	0.003724	0.045315
ILMN_1674719	1	NID1		-0.05	0.003726	0.045318
ILMN_1654357	18	ZNF24		-0.04	0.003731	0.045357
ILMN_3202863	8	LOC389662		-0.06	0.003739	0.045419
ILMN_2127624	6	UBE2CBP		-0.03	0.003742	0.045439
ILMN_1657871	2	RSAD2		-0.08	0.003746	0.045439
ILMN_2106449	8	PLEKHF2		-0.06	0.003746	0.045439
ILMN_2044617	8	MTERFD1		-0.08	0.003751	0.04547
ILMN_1667577	15	LCMT2		-0.07	0.003757	0.045486
ILMN_2350823	2	KCNIP3		0.06	0.003754	0.045486
ILMN_1751378	5	RARS		0.08	0.003771	0.045639
ILMN_1703650	5	TNIP1		0.13	0.00379	0.045811
ILMN_1652797	15	FAM174B		-0.12	0.003795	0.045834
ILMN_1713147	12	MCRS1		-0.05	0.003796	0.045834
ILMN_1657087	2	ZNF533		0.21	0.003806	0.045937
ILMN_1724540	12	CART1		-0.05	0.003814	0.045977
ILMN_2318643	18	TGIF1		-0.09	0.003824	0.045983
ILMN_1693766	4	CEP135		-0.06	0.003817	0.045983

ILMN_2412822	11	SCN3B	0.04	0.003827	0.045983
ILMN_2274531	19	ZNF586	-0.03	0.003821	0.045983
ILMN_2375386	16	RNPS1	-0.03	0.00383	0.045994
ILMN_3249456	9	LOC728034	-0.02	0.003837	0.046052
ILMN_2307266	3	ARL6	-0.03	0.003846	0.046137
ILMN_3236653	1	RNU1-5	0.20	0.003851	0.046175
ILMN_1713031	8	PGCP	-0.10	0.003859	0.046192
ILMN_1696021	1	KPNA6	-0.08	0.003856	0.046192
ILMN_1651628	10	EXOC6	-0.08	0.003858	0.046192
ILMN_1746561	14	BCL2L2	-0.12	0.003873	0.046302
ILMN_1773307	4	NAP1L5	-0.08	0.003874	0.046302
ILMN_2150402	8	TMEM64	-0.08	0.003878	0.04632
ILMN_1723912	1	IFI44L	-0.21	0.003887	0.046337
ILMN_1719303	17	P4HB	0.14	0.003885	0.046337
ILMN_1743352	6	TBCC	-0.06	0.003888	0.046337
ILMN_3247844	4	C4orf10	-0.02	0.003886	0.046337
ILMN_1723522	12	APOLD1	-0.27	0.003908	0.04646
ILMN_1670145	7	DFNA5	0.16	0.003903	0.04646
ILMN_3240997	5	ARAP3	-0.13	0.003904	0.04646
ILMN_1698367	1	CD84	0.10	0.003907	0.04646
ILMN_1755850	19	ZNF350	-0.07	0.003909	0.04646
ILMN_1688629	19	ZNF274	-0.10	0.003911	0.046467
ILMN_1772976	5	BTNL9	-0.10	0.003921	0.046538
ILMN_1781135	2	STAM2	-0.06	0.003926	0.046569
ILMN_1796165	14	GLRX5	-0.10	0.003931	0.0466
ILMN_1798975	7	EGFR	0.23	0.003938	0.046621
ILMN_2384513	21	C2CD2	-0.06	0.003939	0.046621
ILMN_1685803	10	AKR1CL1	0.03	0.003938	0.046621
ILMN_2102670	3	GATA2	-0.09	0.003942	0.046626
ILMN_1671603	8	MED30	-0.09	0.003953	0.046728
ILMN_1812479	10	ATE1	-0.03	0.003954	0.046728
ILMN_1757272	8	THAP1	-0.07	0.003959	0.046763
ILMN_3176146	6	LOC100128918	0.05	0.003963	0.046772
ILMN_1679782	10	BLOC1S2	-0.04	0.003964	0.046772
ILMN_1677221	5	PTCD2	0.04	0.00397	0.046807
ILMN_1807540	10	CBARA1	-0.10	0.003981	0.046887
ILMN_2222651	9	MAK10	-0.04	0.003987	0.046934
ILMN_2395974	22	PRDX3	-0.19	0.003996	0.047023
ILMN_1789138	8	PLEKHA2	-0.04	0.003999	0.047033
ILMN_1652825	11	IL10RA	0.13	0.004004	0.047067
ILMN_1798886	16	NUDT21	-0.10	0.004021	0.047202
ILMN_2415572	14	GALC	-0.04	0.004022	0.047202
ILMN_3234547	1	LOC100133803	-0.03	0.004019	0.047202
ILMN_2316386	2	GPBAR1	-0.06	0.004032	0.047239
ILMN_1746986	19	SLC39A3	-0.03	0.004028	0.047239
ILMN_2352921	7	BPGM	-0.10	0.004046	0.047361
ILMN_1782273	4	N4BP2	0.05	0.004047	0.047361
ILMN_1720829	19	ZFP36	-0.24	0.004058	0.047421
ILMN_2226628	6	ZNF184	-0.05	0.004062	0.047438
ILMN_1715636	7	EIF3B	0.09	0.00407	0.047491
ILMN_2290007	9	COBRA1	-0.05	0.004071	0.047491
ILMN_1732187	19	TMEM143	0.08	0.004076	0.04753
ILMN_1786211	15	HERC1	-0.08	0.004102	0.047761
ILMN_3263601	8	LOC100129093	0.03	0.004101	0.047761
ILMN_1702265	18	HDHD2	-0.09	0.004124	0.047986
ILMN_2325168	11	ARRB1	-0.07	0.004129	0.048016
ILMN_1752343	10	FAM13C1	-0.05	0.004131	0.048016
ILMN_2378100	4	FBXL5	-0.16	0.004133	0.048021
ILMN_1805696	1	DFFA	-0.02	0.004153	0.048224
ILMN_1809245	22	PITPNB	-0.04	0.004173	0.048427
ILMN_1812278	1	LY9	0.05	0.004176	0.048444
ILMN_1682176	3	CLEC3B	-0.20	0.004214	0.048474
ILMN_2066858	13	TNFSF13B	0.16	0.004211	0.048474
ILMN_1661945	14	C14orf156	-0.11	0.004203	0.048474
ILMN_2193591	11	UNC93B1	0.09	0.004204	0.048474
ILMN_1807925	14	GNG2	-0.09	0.004202	0.048474
ILMN_1695711	5	FAM105A	0.09	0.004211	0.048474
ILMN_1668369	19	CDC37	-0.08	0.004194	0.048474
ILMN_3307772	22	PACSIN2	-0.07	0.0042	0.048474
ILMN_1753440	4	DCAF16	-0.07	0.004188	0.048474
ILMN_1726437	17	EFCAB3	0.06	0.004199	0.048474
ILMN_1660856	13	ALG11	-0.05	0.004206	0.048474
ILMN_1765954	9	HSD17B3	0.05	0.004196	0.048474
ILMN_1679256	15	AP3B2	0.04	0.004207	0.048474
ILMN_1771962	7	GLI3	-0.03	0.004214	0.048474
ILMN_1670764	21	USP25	-0.03	0.004196	0.048474
ILMN_2314244	12	LRRC23	0.08	0.004222	0.048536
ILMN_1682781	19	TEAD2	-0.15	0.004229	0.048562
ILMN_1686957	18	SS18	-0.02	0.004231	0.048562
ILMN_2402629	16	METTL9	-0.06	0.004246	0.048697
ILMN_1659352	14	KCNK13	0.05	0.004247	0.048697
ILMN_1661802	2	RAPGEF4	-0.10	0.004256	0.048756
ILMN_1801403	4	DCUN1D4	-0.09	0.004256	0.048756
ILMN_3259146	19	LOC100129681	0.17	0.004263	0.048805
ILMN_3267017	2	LOC100129028	0.14	0.004276	0.048847
ILMN_1684205	15	CIB1	0.09	0.004272	0.048847
ILMN_1734194	9	EXOSC3	-0.07	0.004278	0.048847
ILMN_3185429	19	LOC100129842	-0.02	0.004276	0.048847
ILMN_1757439	11	RBM4	-0.08	0.004283	0.048889
ILMN_2387553	14	PSMA3	-0.11	0.004288	0.04892
ILMN_2357272	6	BCLAF1	-0.08	0.004304	0.049071
ILMN_3216125	3	LOC728126	0.12	0.004307	0.049084
ILMN_1734010	10	C10orf118	-0.04	0.004323	0.049242
ILMN_1777591	10	C10orf65	0.21	0.004326	0.049251
ILMN_1682831	2	NEB	0.15	0.004348	0.049391
ILMN_1701696	17	LOC644863	-0.12	0.004343	0.049391
ILMN_2385278	14	WDR21A	-0.05	0.004347	0.049391
ILMN_1654331	17	HOXB4	-0.04	0.004345	0.049391
ILMN_1712786	7	AHCYL2	0.13	0.004356	0.049432
ILMN_1787518	9	GSN	-0.07	0.004356	0.049432
ILMN_1758315	3	SLC9A9	0.14	0.004371	0.049577
ILMN_1685378	1	LOC728973	0.13	0.004385	0.049718
ILMN_1736654	9	PIP5K1B	-0.03	0.004392	0.04977
ILMN_1729281	19	SPHK2	-0.09	0.004408	0.049897
ILMN_2313851	1	OSBPL9	-0.08	0.004406	0.049897
ILMN_1782050	8	CEBPD	-0.15	0.004418	0.049918

Supplementary Table 8

List of top genes used for replication in TCGA-KIRC tumour samples

ID	Gene	Log Fold Change	Q value
ENSG00000154620	TMSB4Y	5.44	2.99E-91
ENSG00000165246	NLGN4Y	6.36	1.29E-83
ENSG00000233864	TTTY15	6.08	6.56E-83
ENSG00000067646	ZFY	6.17	3.73E-75
ENSG00000099725	PRKY	5.61	5.60E-69
ENSG00000198692	EIF1AY	5.89	2.93E-67
ENSG00000183878	UTY	5.80	1.29E-65
ENSG00000131002	CYorf15A	5.56	1.62E-62
ENSG00000229807	XIST	-5.74	1.05E-58
ENSG00000129824	RPS4Y1	6.04	3.14E-57
ENSG00000114374	USP9Y	5.34	1.14E-54
ENSG00000012817	JARID1D	5.19	2.38E-54
ENSG00000176728	TTTY14	4.73	1.38E-48
ENSG00000126012	JARID1C	-0.65	2.53E-47
ENSG00000225470	LOC554203	-0.66	1.17E-45
ENSG00000147050	UTX	-0.72	3.28E-38
ENSG00000005889	ZFX	-0.56	1.14E-37
ENSG00000183479	TREX2	2.36	2.58E-33
ENSG00000213397	UCHL5IP	1.78	2.32E-29
ENSG00000215301	DDX3X	-0.55	3.66E-27
ENSG00000130021	HDHD1A	-0.56	6.17E-27
ENSG00000135205	CCDC146	2.26	3.80E-24
ENSG00000006756	ARSD	-0.53	6.89E-24
ENSG00000188060	RAB42	1.76	1.28E-20
ENSG00000173674	EIF1AX	-0.47	4.84E-19
ENSG00000118156	ZNF541	1.51	3.90E-18
ENSG00000072501	SMC1A	-0.44	5.86E-18
ENSG00000196188	CTSE	2.36	1.60E-17
ENSG00000187726	DNAJB13	1.78	5.44E-17
ENSG00000026297	RNASET2	1.13	5.40E-17
ENSG00000081985	IL12RB2	2.18	1.04E-16
ENSG00000234685	LOC646208	1.80	1.56E-16
ENSG00000214978	LOC644070	4.04	4.01E-16
ENSG00000163735	CXCL5	2.90	5.35E-16
ENSG00000134533	RERG	0.83	5.31E-15
ENSG00000103034	NDRG4	-1.11	1.03E-14
ENSG00000186312	CA5BP	-0.34	2.56E-14
ENSG00000198648	STK39	0.90	4.03E-14
ENSG00000162383	SLC1A7	1.80	1.90E-13
ENSG00000148600	PCDH21	1.89	2.00E-13
ENSG00000169249	U2AF1L2	-0.36	2.44E-13
ENSG00000132128	LRRC41	0.66	5.17E-13
ENSG00000178776	C5orf46	2.08	5.71E-13
ENSG00000115590	IL1R2	2.09	1.61E-12
ENSG00000144395	CCDC150	-0.85	1.93E-12
ENSG00000138100	TRIM54	2.01	2.38E-12
ENSG00000156398	SFXN2	-0.55	7.02E-12
ENSG00000243709	LEFTY1	2.24	1.21E-11
ENSG00000167992	VWCE	1.23	1.54E-11
ENSG00000158169	FANCC	-0.46	2.03E-11
ENSG00000145088	EAF2	-0.57	6.76E-11
ENSG00000084674	APOB	2.17	7.11E-11
ENSG00000151062	CACNA2D4	0.82	1.21E-10
ENSG00000140403	DNAJA4	0.67	1.46E-10
ENSG00000101040	ZMYND8	0.42	1.74E-10
ENSG00000165140	FBP1	-0.77	1.81E-10
ENSG00000149596	JPH2	0.84	1.85E-10
ENSG00000156265	C21orf7	0.73	2.82E-10
ENSG00000157502	MUM1L1	1.55	3.82E-10
ENSG00000136854	STXBP1	-0.63	4.39E-10
ENSG00000188373	C10orf99	2.33	4.75E-10
ENSG00000256713	PGA5	1.39	5.28E-10
ENSG00000076382	SPAG5	-0.70	5.60E-10
ENSG00000149531	MGC72104	0.46	9.17E-10
ENSG00000131981	LGALS3	0.80	9.96E-10
ENSG00000012171	SEMA3B	0.89	1.34E-09
ENSG00000162639	C1orf59	0.49	1.37E-09
ENSG00000109667	SLC2A9	0.68	1.50E-09
ENSG00000130985	UBE1	-0.26	1.72E-09
ENSG00000149256	ODZ4	0.87	1.97E-09
ENSG00000100353	EIF3D	0.23	2.07E-09
ENSG00000165269	AQP7	-0.86	2.21E-09
ENSG00000168528	SERINC2	0.67	2.28E-09
ENSG00000161944	ASGR2	1.02	2.82E-09
ENSG00000161653	NAGS	0.85	3.11E-09
ENSG00000118515	SGK1	-0.45	3.21E-09
ENSG00000100997	ABHD12	0.33	3.39E-09
ENSG00000198130	HIBCH	-0.42	3.80E-09
ENSG00000106366	SERPINE1	1.10	4.78E-09
ENSG00000147255	IGSF1	-0.95	4.86E-09
ENSG00000170545	SMAGP	0.40	5.77E-09
ENSG00000136870	ZNF189	-0.54	6.20E-09
ENSG00000242779	ZNF702P	0.63	7.10E-09
ENSG00000142197	DOPEY2	0.47	7.62E-09
ENSG00000187957	DNER	1.87	8.62E-09
ENSG00000134240	HMGCS2	-1.49	1.13E-08
ENSG00000198034	RPS4X	-0.32	1.15E-08
ENSG00000133083	DCLK1	0.77	1.57E-08
ENSG00000188677	PARVB	0.54	1.73E-08
ENSG00000180861	C12orf36	2.04	2.00E-08
ENSG00000088002	SULT2B1	1.09	2.35E-08
ENSG00000161649	CD300LG	-1.08	2.74E-08
ENSG00000135525	MAP7	-0.63	3.35E-08
ENSG00000166840	GLYATL1	-0.84	3.61E-08
ENSG00000179314	WSCD1	0.66	3.61E-08
ENSG00000147642	GOLSYN	1.09	3.65E-08
ENSG00000198944	ANKRD43	1.34	4.67E-08
ENSG00000006757	PNPLA4	-0.37	4.81E-08
ENSG00000126562	WNK4	0.86	4.99E-08
ENSG00000121440	PDZRN3	-0.69	5.05E-08
ENSG00000048471	SNX29	-0.38	6.12E-08
ENSG00000145685	LHFPL2	0.50	6.21E-08
ENSG00000101940	WDR13	0.36	6.40E-08
ENSG00000174837	EMR1	0.73	7.17E-08
ENSG00000132823	C20orf111	0.30	7.58E-08
ENSG00000171365	CLCN5	-0.54	7.88E-08
ENSG00000116882	HAO2	-1.06	8.49E-08
ENSG00000116138	DNAJC16	-0.25	8.98E-08
ENSG00000138356	AOX1	0.96	9.17E-08
ENSG00000133475	GGT2	-1.04	9.38E-08
ENSG00000152782	PANK1	-0.61	9.60E-08
ENSG00000172346	CSDC2	-1.26	9.77E-08
ENSG00000104998	IL27RA	0.43	1.02E-07
ENSG00000262655	SPON1	-1.15	1.05E-07
ENSG00000182240	BACE2	0.76	1.08E-07
ENSG00000112541	PDE10A	0.96	1.11E-07
ENSG00000105612	DNASE2	0.32	1.30E-07
ENSG00000196739	COL27A1	-0.55	1.35E-07
ENSG00000064205	WISP2	1.46	1.55E-07
ENSG00000198829	SUCNR1	-0.75	1.66E-07
ENSG00000179921	GPBAR1	-0.51	1.66E-07
ENSG00000115884	SDC1	0.66	1.67E-07
ENSG00000197891	SLC22A12	-1.36	1.79E-07
ENSG00000236882	FIS	1.75	1.91E-07
ENSG00000157693	C9orf91	0.30	2.04E-07
ENSG00000104635	SLC39A14	0.61	2.13E-07
ENSG00000172955	ADH6	-0.84	2.28E-07
ENSG00000007314	SCN4A	-0.77	2.35E-07
ENSG00000160801	PTH1R	-0.74	2.42E-07
ENSG00000152086	TUBA3E	1.97	2.59E-07
ENSG00000106268	NUDT1	0.37	2.65E-07
ENSG00000111319	SCNN1A	1.29	2.84E-07
ENSG00000214960	LOC729920	0.39	3.05E-07
ENSG00000081307	UBE1DC1	-0.34	3.18E-07
ENSG00000175806	MSRA	-0.46	3.25E-07
ENSG00000101255	TRIB3	0.71	3.86E-07
ENSG00000165983	PTER	0.74	3.97E-07
ENSG00000122254	HS3ST2	1.00	4.47E-07
ENSG00000166900	STX3	-0.29	5.10E-07
ENSG00000112964	GHR	-0.62	5.39E-07
ENSG00000182986	ZNF320	0.41	5.67E-07
ENSG00000160808	MYL3	-0.86	5.85E-07
ENSG00000189046	ALKBH2	0.31	5.87E-07
ENSG00000101160	CTSZ	0.37	5.97E-07
ENSG00000090263	MRPS33	0.31	6.36E-07
ENSG00000165810	BTNL9	-0.65	6.55E-07
ENSG00000102466	FGF14	0.88	6.80E-07
ENSG00000144730	IL17RD	-0.52	7.63E-07
ENSG00000180432	CYP8B1	1.09	7.87E-07
ENSG00000169085	C8orf46	0.77	7.91E-07
ENSG00000171425	ZNF581	0.34	8.72E-07
ENSG00000083067	TRPM3	-0.78	1.07E-06
ENSG00000155792	DEPDC6	0.36	1.08E-06
ENSG00000100116	GCAT	0.55	1.08E-06
ENSG00000091129	NRCAM	0.69	1.14E-06
ENSG00000143479	DYRK3	-0.29	1.20E-06
ENSG00000143067	ZNF697	-0.34	1.22E-06
ENSG00000167601	AXL	0.42	1.22E-06
ENSG00000118514	ALDH8A1	-0.87	1.27E-06
ENSG00000169919	GUSB	0.21	1.28E-06
ENSG00000100342	APOL1	0.79	1.34E-06
ENSG00000258484	SPESP1	0.82	1.38E-06
ENSG00000046647	GEMIN8	-0.20	1.39E-06
ENSG00000148218	ALAD	-0.23	1.58E-06
ENSG00000135218	CD36	-0.58	1.61E-06
ENSG00000188706	ZDHHC9	-0.27	1.80E-06
ENSG00000071575	TRIB2	-0.37	1.93E-06
ENSG00000138193	PLCE1	0.36	1.95E-06
ENSG00000213373	FLJ31222	-1.11	2.14E-06
ENSG00000196141	SPATS2L	-0.30	2.19E-06
ENSG00000172023	REG1B	1.81	2.25E-06
ENSG00000119383	PPP2R4	0.28	2.29E-06
ENSG00000107745	CBARA1	-0.23	2.49E-06
ENSG00000138771	SHROOM3	0.66	2.55E-06
ENSG00000114520	SNX4	-0.25	2.65E-06
ENSG00000185499	MUC1	0.73	2.67E-06
ENSG00000108381	ASPA	-0.56	2.71E-06
ENSG00000173918	C1QTNF1	0.59	3.10E-06
ENSG00000102007	PLP2	0.48	3.37E-06
ENSG00000118432	CNR1	0.77	3.51E-06
ENSG00000112337	SLC17A2	-0.85	3.55E-06
ENSG00000095303	PTGS1	0.82	3.68E-06
ENSG00000103154	NECAB2	-0.78	3.84E-06
ENSG00000147180	ZNF711	-0.61	3.86E-06
ENSG00000101000	PROCR	0.38	4.17E-06
ENSG00000237943	LOC439949	0.44	4.23E-06
ENSG00000187288	CIDEC	1.64	4.28E-06
ENSG00000178184	PARD6G	-0.31	4.29E-06
ENSG00000108654	DDX5	-0.21	4.33E-06
ENSG00000189221	MAOA	0.61	5.12E-06
ENSG00000119711	ALDH6A1	-0.49	5.19E-06
ENSG00000108018	SORCS1	-1.07	5.24E-06
ENSG00000100170	SLC5A1	-1.02	5.32E-06
ENSG00000255690	TRIL	-0.41	5.34E-06
ENSG00000177614	PGBD5	-0.59	5.59E-06
ENSG00000122034	GTF3A	0.30	5.73E-06
ENSG00000075886	TUBA3D	1.46	5.84E-06

ENSG00000107679	PLEKHA1	-0.29	6.46E-06
ENSG00000081800	SLC13A1	-1.11	6.61E-06
ENSG00000189292	FAM150B	0.81	6.73E-06
ENSG00000166206	GABRB3	-0.78	7.27E-06
ENSG00000182048	TRPC2	1.16	7.31E-06
ENSG00000153904	DDAH1	-0.37	7.65E-06
ENSG00000113272	THG1L	0.25	7.80E-06
ENSG00000133138	TBC1D8B	0.37	7.90E-06
ENSG00000198795	ZNF521	0.50	8.09E-06
ENSG00000073417	PDE8A	-0.19	8.27E-06
ENSG00000101265	RASSF2	0.39	8.38E-06
ENSG00000090971	NAT14	0.49	8.79E-06
ENSG00000134028	ADAMDEC1	0.89	8.83E-06
ENSG00000196639	HRH1	0.40	8.84E-06
ENSG00000116679	IVNS1ABP	-0.30	9.25E-06
ENSG00000255248	LOC399959	0.57	9.71E-06
ENSG00000021355	SERPINB1	0.20	1.04E-05
ENSG00000174799	CEP135	-0.22	1.10E-05
ENSG00000119523	ALG2	-0.23	1.12E-05
ENSG00000161249	DMKN	0.77	1.21E-05
ENSG00000232368	LOC392437	0.50	1.24E-05
ENSG00000186897	C1QL4	-1.00	1.37E-05
ENSG00000170276	HSPB2	0.48	1.39E-05
ENSG00000243955	GSTA1	-0.76	1.49E-05
ENSG00000171236	LRG1	0.83	1.51E-05
ENSG00000109255	NMU	1.10	1.57E-05
ENSG00000162069	CCDC64B	0.71	1.58E-05
ENSG00000234498	LOC387841	0.44	1.63E-05
ENSG00000166167	BTRC	-0.15	1.66E-05
ENSG00000165300	SLITRK5	-0.92	1.84E-05
ENSG00000101443	WFDC2	0.91	1.88E-05
ENSG00000158467	AHCYL2	0.52	1.88E-05
ENSG00000196459	TRAPPC2	-0.17	1.99E-05
ENSG00000072110	ACTN1	0.32	2.00E-05
ENSG00000062096	ARSF	-0.86	2.00E-05
ENSG00000144452	ABCA12	0.68	2.04E-05
ENSG00000104979	C19orf53	0.33	2.07E-05
ENSG00000183780	SLC35F3	0.86	2.07E-05
ENSG00000170231	FABP6	0.75	2.15E-05
ENSG00000135108	FBXO21	0.29	2.17E-05
ENSG00000204335	SP5	1.10	2.46E-05
ENSG00000178105	DDX10	-0.21	2.49E-05
ENSG00000213366	GSTM2	-0.36	2.58E-05
ENSG00000168952	STXBP6	0.87	2.59E-05
ENSG00000167165	UGT1A6	-0.80	2.65E-05
ENSG00000185043	CIB1	0.31	2.77E-05
ENSG00000103723	AP3B2	0.82	2.89E-05
ENSG00000204439	C6orf47	-0.16	2.94E-05
ENSG00000074410	CA12	0.43	2.99E-05
ENSG00000272143	LOC283481	0.35	3.17E-05
ENSG00000106266	SNX8	0.22	3.31E-05
ENSG00000165801	FLJ10357	-0.24	3.48E-05
ENSG00000145358	DDIT4L	-0.66	3.74E-05
ENSG00000117984	CTSD	0.38	3.78E-05
ENSG00000146066	HIGD2A	0.32	3.79E-05
ENSG00000170323	FABP4	-0.93	3.88E-05
ENSG00000118849	RARRES1	0.85	3.90E-05
ENSG00000171132	PRKCE	-0.28	3.93E-05
ENSG00000197769	MAP1LC3C	-0.56	4.37E-05
ENSG00000150672	DLG2	0.62	4.57E-05
ENSG00000188176	SMTNL2	-0.53	4.62E-05
ENSG00000246705	H2AFJ	0.36	4.68E-05
ENSG00000090013	BLVRB	0.29	4.69E-05
ENSG00000139767	SRRM4	-0.82	4.79E-05
ENSG00000066813	ACSM2B	-0.69	4.93E-05
ENSG00000107362	FAM108B1	-0.22	5.09E-05
ENSG00000196743	GM2A	0.26	5.10E-05
ENSG00000146701	MDH2	0.25	5.59E-05
ENSG00000133321	RARRES3	0.46	5.87E-05
ENSG00000086991	NOX4	-0.46	5.94E-05
ENSG00000058866	DGKG	0.46	6.35E-05
ENSG00000013374	NUB1	0.16	6.43E-05
ENSG00000198576	ARC	-0.56	6.45E-05
ENSG00000204304	PBX2	-0.17	6.48E-05
ENSG00000168795	ZBTB5	-0.18	6.57E-05
ENSG00000143776	CDC42BPA	-0.23	6.62E-05
ENSG00000164093	PITX2	1.33	6.69E-05
ENSG00000184368	MAP7D2	0.90	6.97E-05
ENSG00000102543	CDADC1	-0.20	7.42E-05
ENSG00000164134	NARG1	-0.19	7.92E-05
ENSG00000167291	TBC1D16	0.29	8.06E-05
ENSG00000133606	MKRN1	0.12	8.45E-05
ENSG00000141293	SKAP1	0.44	8.47E-05
ENSG00000154025	SLC5A10	-0.65	8.53E-05
ENSG00000111796	KLRB1	0.40	8.58E-05
ENSG00000118777	ABCG2	-0.47	8.69E-05
ENSG00000173660	UQCRH	-0.30	8.90E-05
ENSG00000148468	FAM171A1	-0.31	8.93E-05
ENSG00000096433	ITPR3	0.38	9.20E-05
ENSG00000247596	TWF2	0.22	9.38E-05
ENSG00000180257	ZNF816A	0.22	9.45E-05
ENSG00000112782	CLIC5	-0.57	9.98E-05
ENSG00000170703	TTLL6	0.72	9.99E-05
ENSG00000172794	RAB37	0.31	0.000101
ENSG00000186377	CYP4X1	-0.34	0.000102
ENSG00000125821	DTD1	0.20	0.000102
ENSG00000096092	TMEM14A	-0.22	0.000106
ENSG00000144891	AGTR1	-0.67	0.000108
ENSG00000177954	RPS27	0.24	0.000109
ENSG00000165633	C10orf72	-0.32	0.00011
ENSG00000128268	MGAT3	0.55	0.000112
ENSG00000213020	ZNF611	0.27	0.000112
ENSG00000183778	B3GALT5	1.03	0.000116
ENSG00000164647	STEAP1	-0.54	0.000117
ENSG00000089060	SLC24A6	0.15	0.000118
ENSG00000003402	CFLAR	-0.20	0.000119
ENSG00000006453	BAIAP2L1	0.52	0.00012
ENSG00000169891	REPS2	0.50	0.000125
ENSG00000115009	CCL20	0.88	0.000131
ENSG00000142235	LMTK3	0.66	0.000132
ENSG00000145014	TMEM44	0.36	0.000135
ENSG00000168234	TTC39C	0.35	0.000142
ENSG00000125968	ID1	-0.41	0.000145
ENSG00000151715	TMEM45B	0.63	0.000151
ENSG00000118564	FBXL5	-0.24	0.000153
ENSG00000099330	OCEL1	0.26	0.000158
ENSG00000050344	NFE2L3	0.36	0.000164
ENSG00000134748	PRPF38A	-0.14	0.000165
ENSG00000138613	APH1B	0.22	0.000166
ENSG00000025800	KPNA6	-0.13	0.000172
ENSG00000150893	FREM2	-0.59	0.000176
ENSG00000070087	PFN2	0.50	0.000178
ENSG00000147724	FAM135B	0.76	0.00018
ENSG00000137343	C6orf134	-0.24	0.000181
ENSG00000197249	SERPINA1	0.61	0.000185
ENSG00000038295	TLL1	-0.45	0.000187
ENSG00000214711	CAPN14	0.76	0.000189
ENSG00000175470	PPP2R2D	-0.13	0.000191
ENSG00000187634	SAMD11	-0.62	0.000193
ENSG00000183337	BCOR	-0.20	0.000196
ENSG00000057704	TMCC3	-0.31	0.000196
ENSG00000178623	GPR35	0.67	0.000199
ENSG00000184304	PRKD1	-0.27	0.000208
ENSG00000119139	TJP2	-0.22	0.000209
ENSG00000156639	ZFAND3	-0.14	0.000221
ENSG00000112799	LY86	0.34	0.000222
ENSG00000218336	ODZ3	0.82	0.000236
ENSG00000111412	C12orf49	-0.27	0.000242
ENSG00000155097	ATP6V1C1	-0.18	0.000245
ENSG00000162407	PPAP2B	-0.30	0.000246
ENSG00000026652	AGPAT4	-0.23	0.000254
ENSG00000008405	CRY1	-0.21	0.000255
ENSG00000143149	ALDH9A1	-0.19	0.000257
ENSG00000041880	PARP3	0.23	0.000264
ENSG00000105355	M6PRBP1	0.26	0.000266
ENSG00000253313	C1orf210	-0.59	0.000267
ENSG00000197275	RAD54B	0.38	0.000269
ENSG00000053328	C6orf186	-0.44	0.00027
ENSG00000071051	NCK2	-0.19	0.000273
ENSG00000173698	GPR64	0.87	0.000273
ENSG00000171345	KRT19	0.98	0.000274
ENSG00000176422	SPRYD4	-0.16	0.000285
ENSG00000197658	MGC34821	-0.78	0.000291
ENSG00000198642	KLHL9	-0.19	0.000299
ENSG00000158716	DUSP23	0.35	0.000313
ENSG00000170290	SLN	1.28	0.000315
ENSG00000069509	FUNDC1	-0.17	0.00033
ENSG00000241935	C10orf65	0.50	0.000335
ENSG00000096060	FKBP5	0.52	0.000337
ENSG00000182326	C1S	0.56	0.000356
ENSG00000176697	BDNF	-0.54	0.000366
ENSG00000107338	SHB	-0.26	0.000369
ENSG00000065675	PRKCQ	0.36	0.000378
ENSG00000166268	C12orf28	-0.60	0.000392
ENSG00000198417	MT1F	0.57	0.000393
ENSG00000165905	GYLTL1B	-0.54	0.000403
ENSG00000008853	RHOBTB2	-0.20	0.000411
ENSG00000169242	EFNA1	0.27	0.000421
ENSG00000104218	CSPP1	-0.18	0.000438
ENSG00000114446	IFT57	-0.17	0.000441
ENSG00000109339	MAPK10	-0.22	0.000479
ENSG00000116106	EPHA4	-0.36	0.000493
ENSG00000133794	ARNTL	-0.21	0.000505
ENSG00000196182	STK40	-0.15	0.000547
ENSG00000155629	PIK3AP1	0.38	0.000562
ENSG00000166448	TMEM130	0.85	0.000563
ENSG00000093167	LRRFIP2	0.12	0.000588
ENSG00000187231	SESTD1	-0.22	0.000588
ENSG00000125740	FOSB	-0.76	0.000597
ENSG00000102524	TNFSF13B	0.40	0.000604
ENSG00000159200	RCAN1	-0.41	0.000627
ENSG00000117450	PRDX1	0.16	0.000643
ENSG00000128016	ZFP36	-0.38	0.00065
ENSG00000113719	ERGIC1	0.21	0.000681
ENSG00000168066	SF1	-0.13	0.000697
ENSG00000131037	EPS8L1	0.62	0.000699
ENSG00000152503	TRIM36	0.35	0.000721
ENSG00000005893	LAMP2	0.20	0.000723
ENSG00000198258	UBL5	0.25	0.000727
ENSG00000188452	CERKL	-0.24	0.000728
ENSG00000100234	TIMP3	-0.41	0.000748
ENSG00000178878	APOLD1	-0.41	0.000749
ENSG00000078053	AMPH	-0.46	0.000759
ENSG00000078487	ZCWPW1	0.18	0.00076
ENSG00000186532	SMYD4	0.15	0.000785
ENSG00000130005	GAMT	0.41	0.000785
ENSG00000179981	TSHZ1	-0.23	0.000801
ENSG00000161395	PGAP3	-0.17	0.000803
ENSG00000154553	PDLIM3	0.38	0.000819
ENSG00000196586	MYO6	-0.21	0.000873

ENSG00000169692	AGPAT2	0.30	0.000881
ENSG00000139637	C12orf10	0.19	0.000881
ENSG00000145545	SRD5A1	0.23	0.000884
ENSG00000028839	TBPL1	0.16	0.000888
ENSG00000145348	MGC16169	-0.12	0.00089
ENSG00000124486	USP9X	-0.19	0.000916
ENSG00000170442	KRT86	0.44	0.000916
ENSG00000133961	NUMB	-0.16	0.00092
ENSG00000122376	FAM35A	-0.17	0.000933
ENSG00000142961	MOBKL2C	-0.12	0.000941
ENSG00000099889	ARVCF	-0.26	0.000941
ENSG00000167515	TRAPPC2L	0.25	0.000969
ENSG00000121671	CRY2	-0.24	0.00097
ENSG00000171606	ZNF274	-0.20	0.000971
ENSG00000163347	CLDN1	0.46	0.000979
ENSG00000123124	WWP1	-0.20	0.001015
ENSG00000198959	TGM2	0.35	0.001026
ENSG00000253719	LOC552889	-0.12	0.001068
ENSG00000128683	GAD1	0.82	0.001072
ENSG00000143409	FAM63A	0.25	0.001079
ENSG00000070756	PABPC1	0.16	0.001083
ENSG00000148672	GLUD1	-0.19	0.001088
ENSG00000101746	NOL4	-0.86	0.001123
ENSG00000233954	UQCRHL	-0.28	0.001159
ENSG00000153898	MCOLN2	0.46	0.001164
ENSG00000072210	ALDH3A2	-0.27	0.001169
ENSG00000256235	C5orf62	0.47	0.001182
ENSG00000167434	CA4	-0.62	0.001187
ENSG00000176399	DMRTA1	-0.35	0.001188
ENSG00000172954	LCLAT1	-0.16	0.001194
ENSG00000197157	SND1	0.16	0.001206
ENSG00000101842	VSIG1	0.49	0.001209
ENSG00000203727	SAMD5	-0.30	0.001251
ENSG00000109320	NFKB1	-0.15	0.001257
ENSG00000174600	CMKLR1	0.31	0.001298
ENSG00000067113	PPAP2A	-0.27	0.00132
ENSG00000118004	COLEC11	-0.41	0.001328
ENSG00000090432	MUL1	-0.16	0.001398
ENSG00000176871	WSB2	0.15	0.001404
ENSG00000167220	HDHD2	-0.16	0.001406
ENSG00000164070	HSPA4L	-0.26	0.001424
ENSG00000055211	C6orf72	-0.16	0.001488
ENSG00000196968	FUT11	-0.17	0.001501
ENSG00000115109	EPB41L5	-0.23	0.001502
ENSG00000142583	SLC2A5	0.48	0.001541
ENSG00000139318	DUSP6	-0.23	0.001559
ENSG00000121989	ACVR2A	-0.15	0.00156
ENSG00000164932	CTHRC1	0.59	0.001563
ENSG00000196345	ZNF167	0.19	0.001566
ENSG00000147113	CXorf36	-0.28	0.001595
ENSG00000146648	EGFR	0.30	0.001614
ENSG00000130208	APOC1	0.52	0.00162
ENSG00000162444	RBP7	-0.39	0.001632
ENSG00000145782	ATG12	0.13	0.001676
ENSG00000125657	TNFSF9	0.40	0.001703
ENSG00000142512	SIGLEC10	0.34	0.001715
ENSG00000162551	ALPL	-0.49	0.00172
ENSG00000115041	KCNIP3	0.54	0.001737
ENSG00000163249	CCNYL1	-0.17	0.001778
ENSG00000137563	GGH	-0.31	0.001873
ENSG00000186994	ANKRD47	-0.29	0.001878
ENSG00000108187	PBLD	-0.33	0.001898
ENSG00000186074	CD300LF	0.30	0.001909
ENSG00000103202	NME4	0.25	0.001913
ENSG00000154217	PITPNC1	-0.21	0.001989
ENSG00000242372	EIF6	0.16	0.001988
ENSG00000122565	CBX3	0.13	0.00201
ENSG00000100284	TOM1	0.18	0.002014
ENSG00000146674	IGFBP3	0.35	0.002039
ENSG00000001461	NPAL3	0.20	0.002058
ENSG00000146070	PLA2G7	0.44	0.002108
ENSG00000107779	BMPR1A	-0.13	0.002131
ENSG00000126561	STAT5A	0.19	0.002141
ENSG00000109572	CLCN3	-0.18	0.002163
ENSG00000165443	PHYHIPL	-0.46	0.002192
ENSG00000139988	RDH12	-0.56	0.002214
ENSG00000117676	RPS6KA1	0.18	0.002281
ENSG00000114115	RBP1	0.34	0.002281
ENSG00000060138	CSDA	0.19	0.002289
ENSG00000239305	RNF103	-0.11	0.002291
ENSG00000140264	SERF2	0.26	0.002314
ENSG00000145681	HAPLN1	0.46	0.002321
ENSG00000140990	NDUFB10	0.22	0.002356
ENSG00000197006	METTL9	-0.18	0.002395
ENSG00000181788	SIAH2	-0.16	0.002403
ENSG00000154229	PRKCA	0.26	0.00241
ENSG00000168591	TMUB2	-0.10	0.00242
ENSG00000140749	IGSF6	0.28	0.002438
ENSG00000034533	ASTE1	-0.10	0.002464
ENSG00000151136	BTBD11	0.61	0.002489
ENSG00000162511	LAPTM5	0.27	0.002493
ENSG00000169087	HSPBAP1	0.17	0.002523
ENSG00000170871	KIAA0232	-0.17	0.002561
ENSG00000174307	PHLDA3	0.29	0.002567
ENSG00000107164	FUBP3	-0.11	0.0026
ENSG00000149131	SERPING1	0.32	0.002703
ENSG00000183298	LOC730029	0.46	0.002721
ENSG00000177125	ZBTB34	-0.13	0.002743
ENSG00000175283	DOLK	-0.15	0.002758
ENSG00000136810	TXN	-0.22	0.002818
ENSG00000185627	PSMD13	0.12	0.002825
ENSG00000120708	TGFBI	0.69	0.002858
ENSG00000163364	LOC375295	0.30	0.002917
ENSG00000164684	ZNF704	-0.25	0.002921
ENSG00000105971	CAV2	0.21	0.002935
ENSG00000088298	EDEM2	0.15	0.002941
ENSG00000082074	FYB	0.32	0.00299
ENSG00000111640	GAPDH	0.20	0.003017
ENSG00000180543	TSPYL5	0.29	0.003065
ENSG00000132470	ITGB4	0.35	0.003106
ENSG00000172201	ID4	-0.34	0.003112
ENSG00000110756	HPS5	0.14	0.003121
ENSG00000100138	NHP2L1	0.14	0.00313
ENSG00000163995	ABLIM2	0.30	0.003167
ENSG00000198805	NP	-0.16	0.003223
ENSG00000136897	MRPL50	-0.16	0.003252
ENSG00000175567	UCP2	0.28	0.00334
ENSG00000101109	STK4	0.14	0.003345
ENSG00000108001	EBF3	-0.38	0.003407
ENSG00000198369	SPRED2	-0.16	0.003443
ENSG00000234618	LOC653162	0.21	0.003489
ENSG00000151883	PARP8	0.15	0.003506
ENSG00000144908	ALDH1L1	-0.45	0.003572
ENSG00000008294	SPAG9	-0.17	0.003588
ENSG00000172939	OXSR1	-0.10	0.003591
ENSG00000255642	LOC341315	0.44	0.003603
ENSG00000136485	DCAF7	-0.13	0.003632
ENSG00000128335	APOL2	0.28	0.003698
ENSG00000128052	KDR	-0.31	0.003776
ENSG00000138468	SENP7	-0.16	0.003806
ENSG00000006715	VPS41	0.14	0.00382
ENSG00000102974	CTCF	-0.09	0.003836
ENSG00000165023	DIRAS2	-0.40	0.003898
ENSG00000224546	LOC100128771	0.25	0.003923
ENSG00000134769	DTNA	0.34	0.003932
ENSG00000115306	SPTBN1	-0.18	0.003975
ENSG00000169495	HTRA4	0.50	0.004021
ENSG00000055813	CCDC85A	-0.35	0.004082
ENSG00000110429	FBXO3	-0.13	0.004186
ENSG00000253540	LOC729375	-0.27	0.004185
ENSG00000130741	EIF2S3	-0.13	0.004218
ENSG00000130313	PGLS	0.19	0.004277
ENSG00000169499	PLEKHA2	-0.18	0.004331
ENSG00000244509	APOBEC3C	0.25	0.004336
ENSG00000122481	RWDD3	-0.13	0.00435
ENSG00000115138	POMC	0.38	0.004351
ENSG00000096746	HNRPH3	-0.10	0.004369
ENSG00000204599	TRIM39	-0.10	0.004376
ENSG00000133019	CHRM3	-0.40	0.004467
ENSG00000028528	SNX1	-0.07	0.004476
ENSG00000158164	TMSB15A	-0.51	0.004527
ENSG00000138308	PLA2G12B	0.61	0.004578
ENSG00000204308	RNF5	-0.16	0.004603
ENSG00000183508	FAM46C	0.34	0.004621
ENSG00000136286	MYO1G	0.30	0.004621
ENSG00000215256	C14orf167	-0.17	0.004687
ENSG00000164056	SPRY1	-0.26	0.004694
ENSG00000100890	KIAA0391	-0.13	0.004766
ENSG00000110066	SUV420H1	-0.09	0.004781
ENSG00000150551	LYPD1	0.53	0.004788
ENSG00000115607	IL18RAP	0.32	0.004875
ENSG00000171444	MCC	-0.22	0.004897
ENSG00000102038	SMARCA1	-0.17	0.005017
ENSG00000175482	POLD4	0.18	0.005188
ENSG00000062725	APPBP2	-0.13	0.005198
ENSG00000172995	ARPP-21	-0.71	0.005306
ENSG00000182901	RGS7	-0.84	0.005318
ENSG00000032219	ARID4A	-0.15	0.005369
ENSG00000106384	MOGAT3	0.57	0.005424
ENSG00000082929	C4orf6	0.46	0.005488
ENSG00000073350	LLGL2	-0.22	0.00555
ENSG00000109606	DHX15	-0.10	0.00569
ENSG00000159212	CLIC6	0.74	0.005847
ENSG00000132824	SERINC3	0.11	0.005896
ENSG00000196263	ZNF471	-0.36	0.006031
ENSG00000109756	RAPGEF2	-0.18	0.006406
ENSG00000237172	B3GNT9	-0.23	0.006421
ENSG00000111729	CLEC4A	0.20	0.006468
ENSG00000135763	URB2	-0.18	0.006489
ENSG00000170515	PA2G4	0.10	0.006574
ENSG00000182512	GLRX5	0.19	0.006646
ENSG00000147912	FBXO10	-0.16	0.006748
ENSG00000163406	SLC15A2	-0.32	0.006863
ENSG00000141873	SLC39A3	0.18	0.0069
ENSG00000118007	STAG1	-0.18	0.006977
ENSG00000170345	FOS	-0.41	0.006998
ENSG00000143507	DUSP10	0.22	0.007157
ENSG00000164967	C9orf23	0.21	0.007205
ENSG00000177613	CSTF2T	-0.10	0.007372
ENSG00000181894	ZNF329	-0.15	0.007457
ENSG00000093144	ECHDC1	-0.13	0.007476
ENSG00000105711	SCN1B	-0.21	0.007481
ENSG00000099284	H2AFY2	0.24	0.007509
ENSG00000163814	CDCP1	0.47	0.00753
ENSG00000167874	TMEM88	-0.28	0.007657
ENSG00000157617	C2CD2	-0.12	0.007839
ENSG00000131269	ABCB7	0.13	0.007913
ENSG00000146085	MUT	-0.18	0.008014
ENSG00000123130	ACOT9	0.10	0.008028
ENSG00000142677	IL22RA1	0.39	0.008037

ENSG00000123472	ATPAF1	-0.12	0.00821
ENSG00000130584	ZBTB46	-0.16	0.008248
ENSG00000023041	ZDHHC6	-0.09	0.00827
ENSG00000047644	WWC3	0.11	0.008378
ENSG00000137501	SYTL2	0.29	0.008506
ENSG00000158747	NBL1	0.39	0.008664
ENSG00000100926	TM9SF1	-0.12	0.008865
ENSG00000237440	LOC100129842	-0.18	0.008934
ENSG00000165813	C10orf118	-0.16	0.009028
ENSG00000166557	TMED3	0.18	0.009083
ENSG00000113643	RARS	0.12	0.009153
ENSG00000121904	CSMD2	-0.30	0.009437
ENSG00000072756	TRNT1	0.13	0.009897
ENSG00000147251	DOCK11	0.23	0.009958
ENSG00000181090	EHMT1	-0.10	0.010061
ENSG00000131089	ARHGEF9	-0.12	0.010118
ENSG00000184384	MAML2	-0.18	0.010402
ENSG00000125726	CD70	0.47	0.010488
ENSG00000170927	PKHD1	-0.39	0.010688
ENSG00000172171	C17orf42	-0.10	0.010771
ENSG00000136802	LRRC8A	-0.14	0.010872
ENSG00000170509	HSD17B13	-0.51	0.010912
ENSG00000081377	CDC14B	-0.16	0.010973
ENSG00000100023	PPIL2	-0.10	0.011065
ENSG00000079689	SCGN	-0.59	0.011107
ENSG00000198908	BHLHB9	-0.15	0.011151
ENSG00000124535	WRNIP1	-0.09	0.011165
ENSG00000100304	TTLL12	0.13	0.011181
ENSG00000213079	RBM16	-0.10	0.011289
ENSG00000053371	AKR7A2	-0.19	0.011302
ENSG00000166831	RBPMS2	-0.25	0.011504
ENSG00000016391	CHDH	-0.26	0.011508
ENSG00000139970	RTN1	0.25	0.011513
ENSG00000106263	EIF3B	0.11	0.01167
ENSG00000172466	ZNF24	-0.12	0.011832
ENSG00000001167	NFYA	-0.09	0.011859
ENSG00000153902	LGI4	0.55	0.012076
ENSG00000139083	ETV6	0.13	0.012134
ENSG00000198538	ZNF28	0.13	0.0125
ENSG00000196511	TPK1	0.17	0.012504
ENSG00000168497	SDPR	-0.29	0.012538
ENSG00000158714	SLAMF8	0.32	0.012767
ENSG00000119705	C14orf156	0.17	0.012986
ENSG00000142556	ZNF614	-0.19	0.013065
ENSG00000136158	SPRY2	-0.18	0.013189
ENSG00000105700	C19orf50	0.09	0.01343
ENSG00000109184	DCUN1D4	-0.10	0.013521
ENSG00000101856	PGRMC1	-0.16	0.013619
ENSG00000015413	DPEP1	0.58	0.013628
ENSG00000184743	ATL3	0.11	0.013678
ENSG00000137673	MMP7	0.59	0.014021
ENSG00000100292	HMOX1	0.24	0.014434
ENSG00000179178	TMEM125	-0.35	0.014515
ENSG00000121691	CAT	-0.20	0.014665
ENSG00000172927	MYEOV	0.62	0.014745
ENSG00000102897	LYRM1	0.18	0.014834
ENSG00000171408	PDE7B	-0.21	0.014834
ENSG00000127081	ZNF484	-0.13	0.015188
ENSG00000196562	SULF2	0.27	0.015408
ENSG00000180667	YOD1	-0.13	0.01554
ENSG00000028310	BRD9	0.10	0.015618
ENSG00000127481	UBR4	-0.13	0.015645
ENSG00000121413	ZSCAN18	-0.17	0.015905
ENSG00000111981	ULBP1	-0.40	0.015924
ENSG00000149346	C20orf94	0.10	0.015983
ENSG00000135111	TBX3	-0.26	0.01619
ENSG00000163568	AIM2	0.39	0.016612
ENSG00000137834	SMAD6	-0.17	0.016746
ENSG00000074935	TUBE1	-0.15	0.016798
ENSG00000161544	CYGB	-0.23	0.016852
ENSG00000091428	RAPGEF4	-0.22	0.016852
ENSG00000112763	BTN2A1	-0.10	0.016947
ENSG00000130822	PNCK	0.54	0.017284
ENSG00000124789	NUP153	-0.12	0.017312
ENSG00000168806	LCMT2	-0.11	0.017413
ENSG00000169764	UGP2	0.12	0.017469
ENSG00000129351	ILF3	-0.07	0.017624
ENSG00000176407	KCMF1	0.08	0.017751
ENSG00000130768	SMPDL3B	0.35	0.017774
ENSG00000144567	FAM134A	-0.11	0.017779
ENSG00000198626	RYR2	0.51	0.017818
ENSG00000183354	KIAA2026	-0.12	0.017988
ENSG00000106789	CORO2A	0.27	0.018126
ENSG00000064547	LPAR2	0.21	0.018171
ENSG00000070770	CSNK2A2	0.09	0.018298
ENSG00000009830	POMT2	-0.12	0.018377
ENSG00000069702	TGFBR3	-0.18	0.019059
ENSG00000182362	C21orf57	0.14	0.019084
ENSG00000198093	ZNF649	-0.16	0.019187
ENSG00000165879	FRAT1	0.16	0.019321
ENSG00000188807	TMEM201	-0.12	0.019536
ENSG00000197329	PELI1	-0.12	0.019647
ENSG00000133328	HRASLS2	0.32	0.019695
ENSG00000100697	DICER1	-0.12	0.01999
ENSG00000087250	MT3	0.64	0.020081
ENSG00000176387	HSD11B2	0.46	0.020181
ENSG00000229809	ZNF688	0.16	0.020469
ENSG00000119927	GPAM	0.15	0.020501
ENSG00000144857	BOC	-0.19	0.020992
ENSG00000258498	DIO3OS	0.51	0.021062
ENSG00000095970	TREM2	0.26	0.021304
ENSG00000083828	ZNF586	-0.11	0.021855
ENSG00000135124	P2RX4	0.13	0.021964
ENSG00000133703	KRAS	-0.12	0.022041
ENSG00000182870	GALNT9	-0.44	0.022114
ENSG00000173917	HOXB2	-0.17	0.022189
ENSG00000163629	PTPN13	-0.17	0.022207
ENSG00000126821	SGPP1	-0.15	0.022337
ENSG00000118898	PPL	0.24	0.02325
ENSG00000187189	TSPYL4	-0.12	0.023369
ENSG00000174165	ZDHHC24	0.11	0.023528
ENSG00000002822	MAD1L1	0.13	0.023781
ENSG00000170473	WIBG	0.11	0.02403
ENSG00000122882	HSGT1	-0.08	0.024216
ENSG00000245937	FLJ33630	0.09	0.024212
ENSG00000107984	DKK1	0.46	0.024216
ENSG00000163162	RNF149	0.11	0.024311
ENSG00000196072	BLOC1S2	0.11	0.024901
ENSG00000102265	TIMP1	0.27	0.025044
ENSG00000145022	TCTA	-0.13	0.025349
ENSG00000169291	SHE	-0.20	0.025438
ENSG00000189241	TSPYL1	-0.14	0.02585
ENSG00000136867	SLC31A2	0.19	0.02592
ENSG00000215548	LOC642236	0.33	0.026066
ENSG00000102804	TSC22D1	-0.13	0.026505
ENSG00000187866	FAM122A	-0.11	0.026647
ENSG00000109738	GLRB	0.47	0.026664
ENSG00000117010	ZNF684	-0.16	0.026853
ENSG00000011523	CEP68	-0.13	0.027041
ENSG00000152778	IFIT5	-0.11	0.0273
ENSG00000107562	CXCL12	-0.25	0.027553
ENSG00000166845	C18orf54	0.15	0.027758
ENSG00000170962	PDGFD	-0.24	0.027926
ENSG00000124562	SNRPC	0.10	0.028357
ENSG00000073712	FERMT2	-0.13	0.028368
ENSG00000066294	CD84	0.26	0.028412
ENSG00000102048	ASB9	-0.18	0.028907
ENSG00000100196	KDELR3	0.30	0.029484
ENSG00000198286	CARD11	0.30	0.029687
ENSG00000185745	IFIT1	-0.17	0.029866
ENSG00000164758	MED30	0.10	0.029899
ENSG00000137185	ZNF193	-0.10	0.030084
ENSG00000197903	HIST1H2BK	0.25	0.030114
ENSG00000076043	REXO2	-0.10	0.030733
ENSG00000112425	EPM2A	-0.12	0.030733
ENSG00000074696	PTPLAD1	0.19	0.030938
ENSG00000108479	GALK1	0.18	0.031379
ENSG00000177721	C5orf39	0.21	0.031675
ENSG00000014914	MTMR11	-0.20	0.032094
ENSG00000119787	ATL2	-0.11	0.032277
ENSG00000203667	FAM36A	-0.11	0.032288
ENSG00000166974	MAPRE2	0.14	0.032887
ENSG00000114573	ATP6V1A	0.23	0.033097
ENSG00000025434	NR1H3	0.12	0.033634
ENSG00000189298	ZKSCAN3	-0.10	0.034474
ENSG00000213762	ZNF134	-0.11	0.034644
ENSG00000151923	TIAL1	-0.07	0.035157
ENSG00000111371	SLC38A1	0.16	0.03545
ENSG00000156467	UQCRB	0.12	0.037411
ENSG00000169715	MT1E	0.41	0.03752
ENSG00000077254	USP33	-0.07	0.038086
ENSG00000101665	SMAD7	-0.13	0.038152
ENSG00000170445	HARS	0.08	0.038267
ENSG00000196155	PLEKHG4	0.26	0.038343
ENSG00000264538	SUZ12P	-0.16	0.038596
ENSG00000129657	SEC14L1	-0.13	0.038641
ENSG00000111696	NT5DC3	0.24	0.038809
ENSG00000115641	FHL2	0.26	0.039112
ENSG00000133135	RNF128	0.25	0.039238
ENSG00000086061	DNAJA1	-0.10	0.039261
ENSG00000169282	KCNAB1	-0.21	0.040159
ENSG00000029363	BCLAF1	-0.08	0.040323
ENSG00000125966	MMP24	0.24	0.040361
ENSG00000107949	BCCIP	-0.08	0.040577
ENSG00000198889	WDR40B	0.55	0.040685
ENSG00000104643	MTMR9	-0.10	0.040706
ENSG00000050555	LAMC3	-0.33	0.040796
ENSG00000165188	RNF183	0.35	0.041082
ENSG00000114861	FOXP1	0.15	0.0415
ENSG00000143376	SNX27	-0.08	0.042017
ENSG00000130731	C16orf13	0.18	0.042727
ENSG00000177283	FZD8	-0.20	0.043116
ENSG00000144619	CNTN4	-0.22	0.043205
ENSG00000123815	ADCK4	0.09	0.043242
ENSG00000127241	MASP1	-0.28	0.043687
ENSG00000100056	DGCR14	0.09	0.043906
ENSG00000171208	NETO2	0.18	0.04405
ENSG00000128951	DUT	0.09	0.044745
ENSG00000185238	PRMT3	0.11	0.044955
ENSG00000107185	KIAA0258	-0.11	0.045399
ENSG00000118518	RNF146	-0.08	0.045424
ENSG00000177098	SCN4B	-0.24	0.04578
ENSG00000224892	LOC220433	-0.17	0.046468
ENSG00000174306	ZHX3	0.11	0.047561
ENSG00000113594	LIFR	-0.19	0.047602
ENSG00000144229	THSD7B	-0.28	0.047665
ENSG00000129226	CD68	0.23	0.047986
ENSG00000164808	KIAA0146	-0.09	0.047986
ENSG00000215421	ZNF407	-0.10	0.048149
ENSG00000131626	PPFIA1	0.06	0.048521

ENSG00000164318	EGFLAM	0.23	0.048552
ENSG00000167100	SAMD14	-0.16	0.049084
ENSG00000204514	LOC730051	-0.15	0.04955
ENSG00000125651	GTF2F1	0.07	0.049889
ENSG00000143797	MBOAT2	-0.16	0.050478
ENSG00000110057	UNC93B1	0.11	0.05101
ENSG00000183742	MACC1	0.26	0.051046
ENSG00000129473	BCL2L2	-0.12	0.051284
ENSG00000115875	SFRS7	-0.07	0.051576
ENSG00000204130	RUFY2	-0.09	0.051814
ENSG00000168685	IL7R	0.24	0.052029
ENSG00000197894	ADH5	-0.09	0.052727
ENSG00000124134	KCNS1	0.67	0.05306
ENSG00000170325	PRDM10	-0.07	0.053627
ENSG00000049883	PTCD2	0.09	0.053922
ENSG00000213759	UGT2B11	-0.33	0.054212
ENSG00000103257	SLC7A5	0.31	0.05425
ENSG00000128849	CGNL1	0.24	0.054962
ENSG00000128815	WDFY4	0.20	0.055078
ENSG00000103145	HCFC1R1	0.18	0.055851
ENSG00000242265	PEG10	-0.29	0.056129
ENSG00000127954	STEAP4	-0.25	0.056654
ENSG00000068137	PLEKHH3	-0.12	0.05713
ENSG00000104324	PGCP	0.11	0.057644
ENSG00000137824	FAM82A2	-0.07	0.057859
ENSG00000160439	RDH13	0.21	0.058105
ENSG00000159110	IFNAR2	0.10	0.05808
ENSG00000164199	GPR98	-0.24	0.05819
ENSG00000149289	ZC3H12C	-0.12	0.058236
ENSG00000154358	OBSCN	0.18	0.058412
ENSG00000170379	FAM139A	0.13	0.058653
ENSG00000136925	TSTD2	-0.12	0.058675
ENSG00000135968	GCC2	-0.11	0.058962
ENSG00000150760	DOCK1	-0.10	0.058988
ENSG00000161921	CXCL16	0.13	0.060182
ENSG00000019549	SNAI2	0.20	0.060364
ENSG00000151778	SERP2	-0.14	0.060844
ENSG00000172183	ISG20	0.17	0.061243
ENSG00000031081	CDGAP	-0.12	0.062026
ENSG00000131508	UBE2D2	0.07	0.062119
ENSG00000019505	SYT13	0.39	0.062666
ENSG00000152443	ZNF776	-0.08	0.062832
ENSG00000088854	C20orf194	-0.09	0.062935
ENSG00000226306	NPY6R	-0.29	0.062976
ENSG00000100632	ERH	0.09	0.062976
ENSG00000198894	KIAA1737	0.16	0.063003
ENSG00000107372	ZFAND5	-0.13	0.063195
ENSG00000120498	TEX11	0.39	0.063226
ENSG00000120318	ARAP3	-0.14	0.063591
ENSG00000185624	P4HB	0.13	0.065847
ENSG00000168067	MAP4K2	-0.09	0.066081
ENSG00000151414	NEK7	0.10	0.06706
ENSG00000176485	PLA2G16	0.11	0.067679
ENSG00000171223	JUNB	-0.19	0.068243
ENSG00000142459	EVI5L	0.13	0.068278
ENSG00000087237	CETP	-0.18	0.06885
ENSG00000113555	PCDH12	-0.17	0.068934
ENSG00000148426	C10orf47	0.19	0.069241
ENSG00000197321	SVIL	-0.12	0.071077
ENSG00000145675	PIK3R1	0.14	0.071794
ENSG00000170989	S1PR1	-0.17	0.073469
ENSG00000071205	ARHGAP10	0.10	0.073555
ENSG00000110002	VWA5A	0.13	0.074057
ENSG00000154930	ACSS1	-0.13	0.074189
ENSG00000029534	ANK1	0.30	0.07501
ENSG00000120279	MYCT1	-0.16	0.075263
ENSG00000117091	CD48	0.18	0.07586
ENSG00000083814	ZNF671	-0.12	0.076677
ENSG00000042429	MED17	-0.06	0.076743
ENSG00000065717	TLE2	0.17	0.076829
ENSG00000127990	SGCE	-0.16	0.076931
ENSG00000165650	PDZD8	-0.12	0.077807
ENSG00000088812	ATRN	0.10	0.077916
ENSG00000188997	KCTD21	-0.07	0.078045
ENSG00000138378	STAT4	0.14	0.078794
ENSG00000005471	ABCB4	0.26	0.079707
ENSG00000131446	MGAT1	0.08	0.080978
ENSG00000184992	BRI3BP	0.10	0.081666
ENSG00000049130	KITLG	0.19	0.081946
ENSG00000111850	C6orf162	0.08	0.083273
ENSG00000100365	NCF4	0.15	0.083355
ENSG00000169129	AFAP1L2	-0.13	0.08411
ENSG00000119596	YLPM1	-0.09	0.084712
ENSG00000166257	SCN3B	0.25	0.085055
ENSG00000180957	PITPNB	-0.06	0.085218
ENSG00000117472	TSPAN1	0.31	0.085318
ENSG00000163449	TMEM169	0.19	0.085657
ENSG00000096070	BRPF3	-0.09	0.086142
ENSG00000162755	KLHDC9	-0.15	0.086737
ENSG00000185567	AHNAK2	-0.24	0.08751
ENSG00000010810	FYN	-0.11	0.087998
ENSG00000166387	PPFIBP2	0.17	0.08803
ENSG00000183208	C15orf58	0.10	0.088141
ENSG00000151893	C10orf46	0.06	0.088234
ENSG00000151692	RNF144A	-0.14	0.088344
ENSG00000122566	HNRPA2B1	-0.06	0.088353
ENSG00000064300	NGFR	-0.22	0.088673
ENSG00000099194	SCD	0.17	0.089002
ENSG00000131094	C1QL1	0.40	0.089438
ENSG00000145293	ENOPH1	0.08	0.089424
ENSG00000198046	ZNF667	-0.25	0.089629
ENSG00000078403	MLLT10	-0.07	0.090131
ENSG00000126970	ZC4H2	-0.09	0.090504
ENSG00000170390	DCLK2	-0.12	0.09091
ENSG00000176371	ZSCAN2	-0.08	0.091115
ENSG00000244462	RBM12	-0.07	0.091426
ENSG00000082269	FAM135A	-0.14	0.091844
ENSG00000179348	GATA2	0.18	0.092476
ENSG00000197128	ZNF772	-0.13	0.095006
ENSG00000164039	BDH2	-0.10	0.095179
ENSG00000100426	ZBED4	-0.06	0.095799
ENSG00000177432	NAP1L5	-0.10	0.09626
ENSG00000074219	TEAD2	0.12	0.098183
ENSG00000135316	SYNCRIP	-0.07	0.099007
ENSG00000174944	P2RY14	-0.17	0.099506
ENSG00000162441	LZIC	-0.08	0.099698
ENSG00000165526	RPUSD4	0.06	0.100664
ENSG00000172469	MANEA	-0.13	0.100702
ENSG00000122550	KLHL7	0.08	0.102448
ENSG00000136160	EDNRB	-0.20	0.102496
ENSG00000108622	ICAM2	-0.12	0.102952
ENSG00000084731	KIF3C	-0.11	0.103078
ENSG00000224982	TMEM233	-0.17	0.103148
ENSG00000153989	NUS1	-0.08	0.103457
ENSG00000180914	OXTR	0.24	0.104388
ENSG00000158014	SLC30A2	-0.41	0.10501
ENSG00000107593	PKD2L1	0.29	0.105281
ENSG00000118420	UBE2CBP	0.10	0.106854
ENSG00000075239	ACAT1	-0.18	0.106964
ENSG00000226479	TMEM185B	-0.08	0.107377
ENSG00000157657	ZNF618	-0.09	0.107711
ENSG00000139910	NOVA1	0.20	0.107983
ENSG00000165695	C9orf98	0.13	0.108169
ENSG00000035403	VCL	-0.10	0.108911
ENSG00000135697	BCMO1	-0.19	0.110147
ENSG00000198736	SEPX1	0.15	0.110541
ENSG00000234545	FAM133B	-0.07	0.110779
ENSG00000171621	SPSB1	0.18	0.111155
ENSG00000076662	ICAM3	0.15	0.111362
ENSG00000182606	TRAK1	0.08	0.111859
ENSG00000165512	ZNF22	0.07	0.112076
ENSG00000111674	ENO2	0.16	0.112533
ENSG00000109458	GAB1	-0.09	0.112534
ENSG00000171234	UGT2B7	0.21	0.112574
ENSG00000197045	GMFB	-0.10	0.112814
ENSG00000099625	C19orf26	0.21	0.113354
ENSG00000165501	PPIL5	0.09	0.113858
ENSG00000107669	ATE1	-0.08	0.116395
ENSG00000143995	MEIS1	-0.11	0.116595
ENSG00000092096	SLC22A17	0.16	0.117195
ENSG00000215808	LOC339535	0.37	0.117953
ENSG00000232187	FTHL7	-0.11	0.118223
ENSG00000129255	MPDU1	0.09	0.119371
ENSG00000196151	WDSUB1	-0.09	0.12021
ENSG00000146414	SHPRH	-0.12	0.120634
ENSG00000134779	C18orf10	0.07	0.121029
ENSG00000005302	MSL3	0.05	0.121611
ENSG00000139926	FRMD6	-0.13	0.122341
ENSG00000137207	YIPF3	-0.06	0.122529
ENSG00000165006	UBAP1	-0.06	0.122832
ENSG00000101391	CDK5RAP1	0.07	0.12316
ENSG00000170537	TMC7	-0.11	0.123661
ENSG00000124228	DDX27	0.06	0.123927
ENSG00000152315	KCNK13	0.17	0.124317
ENSG00000136895	GARNL3	-0.10	0.12534
ENSG00000196118	C16orf93	-0.16	0.125281
ENSG00000010072	C1orf124	-0.06	0.125715
ENSG00000185009	AP3M1	-0.06	0.125984
ENSG00000120051	CCDC147	-0.13	0.126736
ENSG00000113966	ARL6	0.12	0.128291
ENSG00000152049	KCNE4	-0.15	0.128921
ENSG00000141380	SS18	-0.05	0.129044
ENSG00000068885	IFT80	-0.08	0.129955
ENSG00000159658	KIAA0494	-0.07	0.13094
ENSG00000115145	STAM2	-0.07	0.131088
ENSG00000106780	MEGF9	0.11	0.132363
ENSG00000140319	SRP14	0.06	0.132646
ENSG00000135338	LCA5	0.11	0.132887
ENSG00000112081	SFRS3	-0.05	0.133182
ENSG00000213246	SUPT4H1	0.06	0.133323
ENSG00000148516	ZEB1	-0.11	0.133307
ENSG00000154928	EPHB1	-0.19	0.13507
ENSG00000135040	MAK10	-0.07	0.135728
ENSG00000122224	LY9	0.17	0.136513
ENSG00000117906	RCN2	0.08	0.13677
ENSG00000144749	LRIG1	-0.09	0.136943
ENSG00000134049	IER3IP1	-0.09	0.137196
ENSG00000115468	EFHD1	-0.16	0.137416
ENSG00000075785	RAB7A	-0.06	0.137897
ENSG00000115363	TMEM166	0.13	0.138558
ENSG00000171649	ZIK1	-0.13	0.138717
ENSG00000234899	FLJ37644	0.16	0.139368
ENSG00000137959	IFI44L	-0.16	0.139837
ENSG00000256683	ZNF350	-0.09	0.139965
ENSG00000083642	PDS5B	-0.07	0.140369
ENSG00000124942	AHNAK	0.10	0.140724
ENSG00000144355	DLX1	-0.18	0.140829
ENSG00000165156	ZHX1	-0.08	0.141032
ENSG00000165322	ARHGAP12	0.07	0.142821
ENSG00000114279	FGF12	0.17	0.144777

ENSG00000169375	SIN3A		-0.05	0.145135
ENSG00000100461	RBM23		-0.05	0.145171
ENSG00000127946	HIP1		-0.09	0.14581
ENSG00000063127	SLC6A16		0.22	0.146956
ENSG00000158683	PKD1L1		-0.15	0.147137
ENSG00000079263	SP140		0.17	0.149536
ENSG00000100311	PDGFB		-0.11	0.150385
ENSG00000148688	RPP30		0.06	0.15087
ENSG00000122176	FMOD		0.25	0.151793
ENSG00000122707	RECK		-0.09	0.152637
ENSG00000175104	TRAF6		-0.07	0.152889
ENSG00000080709	KCNN2		0.13	0.153008
ENSG00000175538	KCNE3		-0.13	0.155202
ENSG00000165678	GHITM		0.09	0.15532
ENSG00000238227	C9orf69		-0.07	0.15566
ENSG00000129680	MAP7D3		0.08	0.156406
ENSG00000164736	SOX17		-0.13	0.156678
ENSG00000170315	UBB		0.07	0.157478
ENSG00000196505	GDAP2		-0.05	0.160198
ENSG00000176293	ZNF135		-0.13	0.160632
ENSG00000107371	EXOSC3		-0.06	0.161758
ENSG00000146859	TMEM140		0.09	0.165608
ENSG00000138175	ARL3		-0.07	0.166425
ENSG00000148180	GSN		-0.10	0.167491
ENSG00000136718	IMP4		0.05	0.168519
ENSG00000079459	FDFT1		0.08	0.169452
ENSG00000123545	C6orf66		0.09	0.171235
ENSG00000025796	SEC63		-0.06	0.172839
ENSG00000167996	FTH1		-0.09	0.173316
ENSG00000168916	ZNF608		0.12	0.17352
ENSG00000242259	C22orf39		0.05	0.175778
ENSG00000100106	TRIOBP		-0.06	0.176256
ENSG00000137936	BCAR3		-0.08	0.176321
ENSG00000175691	ZNF77		0.07	0.18047
ENSG00000085377	PREP		0.05	0.181557
ENSG00000108947	EFNB3		-0.17	0.182458
ENSG00000117868	FAM62B		0.07	0.184151
ENSG00000212993	POU5F1P1		-0.14	0.184297
ENSG00000085978	ATG16L1		0.11	0.18452
ENSG00000069431	ABCC9		-0.13	0.185047
ENSG00000120526	NUDCD1		0.07	0.185841
ENSG00000140374	ETFA		-0.07	0.185836
ENSG00000137992	DBT		-0.07	0.186151
ENSG00000178175	ZNF366		-0.15	0.186318
ENSG00000134321	RSAD2		-0.13	0.186401
ENSG00000154767	XPC		0.06	0.186535
ENSG00000006042	TMEM98		0.14	0.187859
ENSG00000183715	OPCML		-0.30	0.193593
ENSG00000184515	BEX5		-0.17	0.194179
ENSG00000178229	ZNF543		-0.06	0.195189
ENSG00000142949	PTPRF		0.10	0.196001
ENSG00000173933	RBM4		-0.04	0.197031
ENSG00000185483	ROR1		-0.11	0.197879
ENSG00000115386	REG1A		0.38	0.199057
ENSG00000196591	HDAC2		-0.06	0.200813
ENSG00000101850	GPR143		-0.31	0.202688
ENSG00000130193	C8orf55		-0.08	0.202679
ENSG00000107672	NSMCE4A		-0.05	0.203099
ENSG00000141497	ZMYND15		0.10	0.203205
ENSG00000173166	RAPH1		-0.12	0.203985
ENSG00000166979	C21orf63		0.10	0.204011
ENSG00000162616	DNAJB4		-0.08	0.204103
ENSG00000124749	COL21A1		-0.18	0.207073
ENSG00000182742	HOXB4		-0.09	0.207427
ENSG00000130821	SLC6A8		0.14	0.208715
ENSG00000105953	OGDH		-0.08	0.210126
ENSG00000105855	ITGB8		0.15	0.210242
ENSG00000118496	FBXO30		0.08	0.21065
ENSG00000083123	BCKDHB		0.10	0.210969
ENSG00000132780	NASP		-0.06	0.211588
ENSG00000165476	REEP3		0.08	0.212009
ENSG00000171428	NAT1		-0.08	0.212617
ENSG00000134343	ANO3		-0.17	0.213117
ENSG00000255112	CHMP1B		-0.05	0.215011
ENSG00000119917	IFIT3		-0.08	0.215384
ENSG00000047249	ATP6V1H		0.09	0.215796
ENSG00000143382	ADAMTSL4		0.15	0.215804
ENSG00000164163	ABCE1		-0.06	0.216079
ENSG00000171204	TMEM126B		0.07	0.21657
ENSG00000100567	PSMA3		0.05	0.217056
ENSG00000148660	CAMK2G		-0.04	0.217456
ENSG00000175445	LPL		-0.14	0.217769
ENSG00000227507	LTB		0.17	0.218959
ENSG00000087301	TXNDC16		-0.09	0.219285
ENSG00000137075	RNF38		-0.07	0.219326
ENSG00000133111	RFXAP		-0.06	0.22357
ENSG00000177426	TGIF1		-0.06	0.224204
ENSG00000104691	UBXN8		-0.07	0.225619
ENSG00000128245	YWHAH		-0.05	0.226778
ENSG00000160049	DFFA		-0.04	0.227064
ENSG00000130758	MAP3K10		-0.07	0.228034
ENSG00000020633	RUNX3		0.12	0.229532
ENSG00000111912	NCOA7		-0.11	0.229756
ENSG00000010626	LRRC23		0.07	0.230492
ENSG00000152348	ATG10		0.05	0.23175
ENSG00000171453	POLR1C		-0.05	0.232128
ENSG00000186106	ANKRD46		-0.07	0.232687
ENSG00000182397	DKFZp434I1020		-0.12	0.233034
ENSG00000180008	SOCS4		-0.07	0.234085
ENSG00000164024	METAP1		0.05	0.234108
ENSG00000139514	SLC7A1		0.12	0.234407
ENSG00000143772	ITPKB		-0.08	0.236404
ENSG00000168070	C11orf85		0.24	0.236465
ENSG00000137168	PPIL1		-0.06	0.236503
ENSG00000136114	THSD1		-0.10	0.239441
ENSG00000253710	ALG11		-0.07	0.240635
ENSG00000099377	HSD3B7		0.13	0.24081
ENSG00000111052	LIN7A		-0.17	0.243026
ENSG00000124688	MAD2L1BP		-0.05	0.245946
ENSG00000137726	FXYD6		-0.11	0.249244
ENSG00000125872	C20orf75		0.25	0.249561
ENSG00000117308	GALE		0.09	0.251589
ENSG00000081087	OSTM1		0.07	0.252665
ENSG00000119929	CUTC		-0.04	0.253263
ENSG00000116161	CACYBP		0.05	0.254112
ENSG00000151327	FAM177A1		-0.06	0.257493
ENSG00000120314	WDR55		0.05	0.258528
ENSG00000176720	BOK		0.10	0.259042
ENSG00000152454	ZNF256		-0.06	0.259588
ENSG00000074603	DPP8		-0.05	0.261368
ENSG00000126945	HNRNPH2		-0.05	0.262671
ENSG00000184216	IRAK1		0.07	0.263122
ENSG00000173559	OBFC2A		0.10	0.265632
ENSG00000034677	RNF19A		-0.06	0.265615
ENSG00000183421	RIPK4		0.09	0.268597
ENSG00000179082	C9orf106		0.15	0.270077
ENSG00000119686	FLVCR2		0.09	0.270837
ENSG00000204385	SLC44A4		0.20	0.271384
ENSG00000168081	PNOC		-0.22	0.271664
ENSG00000011405	PIK3C2A		-0.06	0.2734
ENSG00000162694	EXTL2		-0.05	0.274761
ENSG00000180479	ZNF571		0.05	0.276566
ENSG00000170439	METTL7B		-0.12	0.276814
ENSG00000100883	SRP54		-0.06	0.277596
ENSG00000168286	THAP11		0.04	0.277695
ENSG00000064999	ANKS1A		-0.08	0.277844
ENSG00000112685	EXOC2		-0.04	0.277955
ENSG00000078124	PHCA		0.06	0.279971
ENSG00000108061	SHOC2		-0.05	0.281937
ENSG00000213390	ARHGAP19		-0.05	0.2835
ENSG00000160209	PDXK		0.07	0.285826
ENSG00000040341	STAU2		-0.05	0.287317
ENSG00000050628	PTGER3		-0.20	0.287331
ENSG00000104812	GYS1		0.06	0.289034
ENSG00000171174	RBKS		0.08	0.289734
ENSG00000135297	MTO1		-0.04	0.289746
ENSG00000145901	TNIP1		0.06	0.28986
ENSG00000196284	SUPT3H		0.05	0.290047
ENSG00000070214	SLC44A1		-0.06	0.294761
ENSG00000070718	AP3M2		-0.04	0.295005
ENSG00000167088	SNRPD1		-0.04	0.295224
ENSG00000170340	B3GNT2		-0.05	0.295952
ENSG00000122779	TRIM24		0.04	0.298225
ENSG00000141219	C17orf80		-0.03	0.298731
ENSG00000153944	MSI2		0.08	0.300904
ENSG00000153113	CAST		0.05	0.300886
ENSG00000095787	WAC		-0.04	0.301586
ENSG00000269713	NBPF20		-0.07	0.30376
ENSG00000172269	DPAGT1		-0.04	0.30427
ENSG00000226763	SRRM5		-0.10	0.304793
ENSG00000163516	ANKZF1		-0.08	0.30506
ENSG00000166825	ANPEP		0.14	0.306294
ENSG00000134333	LDHA		0.06	0.306616
ENSG00000150593	PDCD4		-0.05	0.306754
ENSG00000163608	C3orf17		-0.04	0.308618
ENSG00000137463	C4orf49		-0.23	0.314686
ENSG00000215845	LOC100131187		-0.08	0.317425
ENSG00000141646	SMAD4		-0.04	0.324798
ENSG00000134146	ATPBD4		0.06	0.325222
ENSG00000128872	TMOD2		-0.08	0.325284
ENSG00000133812	SBF2		-0.06	0.325947
ENSG00000143374	TARS2		0.04	0.32595
ENSG00000179913	B3GNT3		0.18	0.326175
ENSG00000166189	HPS6		-0.04	0.328598
ENSG00000094631	HDAC6		0.06	0.328608
ENSG00000171604	CXXC5		-0.05	0.328949
ENSG00000108395	TRIM37		0.04	0.329222
ENSG00000185305	ARL15		0.07	0.33174
ENSG00000131055	COX4I2		0.13	0.332242
ENSG00000136147	PHF11		0.05	0.33572
ENSG00000243056	EIF4EBP3		0.08	0.337278
ENSG00000174564	IL20RB		0.27	0.338718
ENSG00000110921	MVK		0.10	0.339611
ENSG00000214106	LOC100132707		0.05	0.34009
ENSG00000133063	CHIT1		0.26	0.341369
ENSG00000126351	THRA		-0.05	0.341872
ENSG00000147378	FATE1		-0.11	0.343052
ENSG00000163815	CLEC3B		-0.12	0.344804
ENSG00000049769	PPP1R3F		0.05	0.344991
ENSG00000151623	NR3C2		0.12	0.346186
ENSG00000025708	TYMP		0.09	0.34657
ENSG00000175274	TP53I11		-0.07	0.347653
ENSG00000100266	PACSIN2		-0.05	0.347877
ENSG00000243811	APOBEC3D		0.08	0.348105
ENSG00000001561	ENPP4		-0.08	0.353595
ENSG00000132170	PPARG		-0.06	0.354326
ENSG00000141034	C17orf39		-0.04	0.355029
ENSG00000166960	C18orf34		-0.12	0.357038
ENSG00000126246	TMEM149		0.10	0.357951
ENSG00000101670	LIPG		0.16	0.361271

ENSG00000159210	SNF8	0.03	0.361824
ENSG00000166965	RCCD1	-0.05	0.362378
ENSG00000133895	MEN1	-0.03	0.363817
ENSG00000110324	IL10RA	0.09	0.364196
ENSG00000188785	ZNF548	-0.04	0.367806
ENSG00000140153	WDR20	-0.04	0.368513
ENSG00000025156	HSF2	0.04	0.371232
ENSG00000143751	C1orf55	-0.05	0.372245
ENSG00000103657	HERC1	-0.05	0.372629
ENSG00000196396	PTPN1	0.05	0.373246
ENSG00000165828	PRAP1	0.20	0.373546
ENSG00000144331	ZNF533	0.17	0.374536
ENSG00000141424	SLC39A6	0.06	0.379553
ENSG00000219626	LOC375190	-0.04	0.379681
ENSG00000142002	DPP9	0.05	0.382294
ENSG00000129562	DAD1	0.04	0.382373
ENSG00000112282	MED23	-0.04	0.384556
ENSG00000127526	SLC35E1	0.04	0.387918
ENSG00000174951	FUT1	-0.09	0.39248
ENSG00000108946	PRKAR1A	-0.04	0.393959
ENSG00000156232	WHAMM	-0.04	0.395037
ENSG00000156469	MTERFD1	0.05	0.395101
ENSG00000172348	RCAN2	-0.11	0.395408
ENSG00000137821	LRRC49	0.05	0.400378
ENSG00000113361	CDH6	0.10	0.403041
ENSG00000112237	CCNC	0.05	0.404587
ENSG00000213341	CHUK	-0.04	0.404905
ENSG00000178445	GLDC	0.13	0.408135
ENSG00000187260	WDR86	0.12	0.408172
ENSG00000138107	ACTR1A	-0.03	0.412314
ENSG00000277494	GPIHBP1	-0.11	0.414129
ENSG00000148019	CEP78	0.05	0.415432
ENSG00000133313	CNDP2	0.06	0.41607
ENSG00000075413	MARK3	-0.03	0.417711
ENSG00000168216	LMBRD1	-0.05	0.418172
ENSG00000107651	SEC23IP	-0.03	0.418394
ENSG00000165506	C14orf104	-0.04	0.420542
ENSG00000062370	ZFP112	-0.05	0.422955
ENSG00000186635	CENTD2	0.05	0.424123
ENSG00000081154	PCNP	-0.04	0.425634
ENSG00000100413	POLR3H	-0.03	0.426598
ENSG00000163320	CGGBP1	-0.04	0.431356
ENSG00000143851	PTPN7	0.09	0.431954
ENSG00000179630	C13orf31	0.05	0.432666
ENSG00000169902	TPST1	-0.05	0.433298
ENSG00000196470	SIAH1	0.03	0.434404
ENSG00000189042	ZNF567	-0.03	0.434987
ENSG00000184371	CSF1	0.05	0.439615
ENSG00000134326	CMPK2	-0.06	0.445127
ENSG00000085563	ABCB1	0.11	0.44697
ENSG00000083937	CHMP2B	-0.04	0.447464
ENSG00000198954	KIAA1279	-0.04	0.447572
ENSG00000116473	RAP1A	0.03	0.448862
ENSG00000155096	AZIN1	0.04	0.452204
ENSG00000121361	KCNJ8	-0.07	0.453588
ENSG00000120265	PCMT1	0.03	0.455261
ENSG00000182359	KBTBD3	-0.05	0.45591
ENSG00000064651	SLC12A2	-0.06	0.457027
ENSG00000124702	KLHDC3	-0.03	0.460396
ENSG00000166741	NNMT	0.09	0.461486
ENSG00000119401	TRIM32	-0.04	0.462141
ENSG00000165806	CASP7	-0.03	0.463231
ENSG00000102309	PIN4	-0.04	0.467888
ENSG00000120696	KBTBD7	-0.04	0.471155
ENSG00000144959	AADACL1	0.07	0.471723
ENSG00000157349	DDX19B	0.02	0.473908
ENSG00000137409	MTCH1	0.03	0.474659
ENSG00000181444	ZNF467	0.06	0.475806
ENSG00000257267	ZNF271	-0.03	0.478216
ENSG00000069667	RORA	-0.05	0.478392
ENSG00000120685	C13orf23	-0.03	0.484594
ENSG00000096654	ZNF184	-0.04	0.485989
ENSG00000148541	FAM13C1	0.07	0.488672
ENSG00000116729	GPR177	-0.06	0.488903
ENSG00000198515	CNGA1	-0.11	0.489762
ENSG00000139350	NEDD1	-0.03	0.494075
ENSG00000083099	LYRM2	0.03	0.496542
ENSG00000155465	SLC7A7	0.10	0.496517
ENSG00000139178	C1RL	0.05	0.498242
ENSG00000105697	HAMP	0.13	0.501755
ENSG00000116962	NID1	-0.06	0.501812
ENSG00000119771	KLHL29	-0.05	0.50193
ENSG00000170873	MTSS1	0.06	0.50367
ENSG00000171747	LGALS4	0.22	0.505924
ENSG00000125735	TNFSF14	0.11	0.506204
ENSG00000129460	NGDN	-0.03	0.507255
ENSG00000165029	ABCA1	0.05	0.508652
ENSG00000084733	RAB10	-0.03	0.51235
ENSG00000006534	ALDH3B1	0.06	0.515675
ENSG00000133985	TTC9	0.07	0.516746
ENSG00000157765	SLC34A2	0.19	0.521486
ENSG00000189410	SH2D5	0.08	0.521982
ENSG00000103769	RAB11A	-0.03	0.523401
ENSG00000176148	TCP11L1	-0.03	0.530759
ENSG00000124767	GLO1	0.03	0.534999
ENSG00000127129	EDN2	0.12	0.535308
ENSG00000171388	APLN	-0.06	0.535297
ENSG00000198453	ZNF568	-0.04	0.536405
ENSG00000185614	C3orf54	0.06	0.542864
ENSG00000154727	GABPA	-0.03	0.549619
ENSG00000134215	VAV3	-0.05	0.551948
ENSG00000182472	CAPN12	-0.08	0.553408
ENSG00000188643	S100A16	0.04	0.553792
ENSG00000102096	PIM2	0.07	0.554913
ENSG00000161542	PRPSAP1	0.02	0.55609
ENSG00000196418	ZNF124	-0.04	0.558823
ENSG00000114315	HES1	-0.05	0.561885
ENSG00000187778	MCRS1	0.03	0.562884
ENSG00000119673	ACOT2	0.05	0.563493
ENSG00000112130	RNF8	-0.02	0.566126
ENSG00000154258	ABCA9	-0.07	0.566435
ENSG00000143742	SRP9	0.03	0.567421
ENSG00000156298	TSPAN7	0.07	0.574813
ENSG00000134717	BTF3L4	0.02	0.574651
ENSG00000165795	NDRG2	0.05	0.574776
ENSG00000160226	C21orf2	-0.03	0.576416
ENSG00000160014	CALM3	-0.02	0.58077
ENSG00000105325	FZR1	-0.02	0.581334
ENSG00000106571	GLI3	0.05	0.585977
ENSG00000101574	METTL4	-0.03	0.586264
ENSG00000131015	ULBP2	-0.05	0.586419
ENSG00000240370	RPL13L	-0.03	0.587065
ENSG00000186469	GNG2	0.04	0.587113
ENSG00000180592	C10orf140	-0.06	0.590344
ENSG00000166770	LOC100128252	-0.06	0.591203
ENSG00000165118	C9orf64	0.03	0.59382
ENSG00000119408	NEK6	-0.04	0.597922
ENSG00000001036	FUCA2	-0.02	0.59926
ENSG00000129422	MTUS1	-0.04	0.600451
ENSG00000180745	CLRN3	0.07	0.604101
ENSG00000112335	SNX3	-0.03	0.605506
ENSG00000099864	PALM	-0.05	0.606786
ENSG00000167005	NUDT21	0.03	0.609153
ENSG00000138190	EXOC6	-0.03	0.614481
ENSG00000111875	ASF1A	0.03	0.615473
ENSG00000159579	RSPRY1	0.02	0.61992
ENSG00000132744	ACY3	0.06	0.625544
ENSG00000149798	CDC42EP2	-0.04	0.627898
ENSG00000169239	CA5B	-0.03	0.634874
ENSG00000265972	TXNIP	-0.03	0.635892
ENSG00000177888	ZBTB41	0.03	0.6365
ENSG00000134285	FKBP11	0.05	0.636587
ENSG00000163257	DCAF16	-0.03	0.640053
ENSG00000109332	UBE2D3	-0.02	0.640351
ENSG00000156510	HKDC1	-0.06	0.640853
ENSG00000128989	ARPP19	0.02	0.640967
ENSG00000072042	RDH11	0.04	0.64243
ENSG00000130948	HSD17B3	-0.08	0.64343
ENSG00000144677	CTDSPL	0.03	0.643988
ENSG00000242732	RGAG4	-0.03	0.644519
ENSG00000100601	ALKBH1	0.02	0.64622
ENSG00000225766	DHRS4L1	-0.05	0.648033
ENSG00000134574	DDB2	0.02	0.651871
ENSG00000101752	MIB1	0.03	0.659107
ENSG00000162688	AGL	-0.03	0.66083
ENSG00000205593	FAM116B	0.03	0.665458
ENSG00000111321	LTBR	0.02	0.66738
ENSG00000125249	RAP2A	-0.02	0.671722
ENSG00000069712	KIAA1107	-0.03	0.675005
ENSG00000107551	RASSF4	0.03	0.678666
ENSG00000188343	FAM92A1	0.03	0.683325
ENSG00000105401	CDC37	-0.02	0.686187
ENSG00000107771	FAM190B	-0.02	0.690401
ENSG00000127334	DYRK2	-0.02	0.690962
ENSG00000156052	GNAQ	-0.02	0.692586
ENSG00000086475	SEPHS1	0.01	0.69332
ENSG00000123091	RNF11	-0.02	0.693759
ENSG00000198015	MRPL42	-0.02	0.695563
ENSG00000154589	LY96	0.04	0.695677
ENSG00000181847	TIGIT	0.06	0.697396
ENSG00000165572	KBTBD6	-0.02	0.698184
ENSG00000132464	ENAM	-0.07	0.700154
ENSG00000198964	SGMS1	0.02	0.701018
ENSG00000119599	WDR21A	0.02	0.705079
ENSG00000182400	TRAPPC6B	0.02	0.70591
ENSG00000011198	ABHD5	0.02	0.709405
ENSG00000180694	TMEM64	0.03	0.709518
ENSG00000182749	PAQR7	-0.02	0.713437
ENSG00000177463	NR2C2	0.02	0.720548
ENSG00000124523	SIRT5	-0.02	0.720463
ENSG00000172831	CES2	0.03	0.721157
ENSG00000135744	AGT	-0.05	0.722965
ENSG00000156650	MYST4	-0.02	0.723191
ENSG00000173545	ZNF622	-0.02	0.731755
ENSG00000100711	ZFYVE21	-0.02	0.734435
ENSG00000155313	USP25	0.01	0.735765
ENSG00000241370	RPP21	0.02	0.739536
ENSG00000122378	C10orf58	-0.02	0.740796
ENSG00000148943	LIN7C	-0.02	0.741345
ENSG00000180354	C7orf41	-0.03	0.741127
ENSG00000176714	CCDC121	0.02	0.743146
ENSG00000023445	BIRC3	0.04	0.752259
ENSG00000204531	POU5F1	-0.04	0.752281
ENSG00000198835	GJC2	0.04	0.755435
ENSG00000148835	TAF5	-0.01	0.758343
ENSG00000115604	IL18R1	-0.03	0.761126
ENSG00000185651	UBE2L3	0.01	0.761693
ENSG00000205362	MT1A	0.06	0.76185
ENSG00000205133	C8orf83	-0.02	0.768405
ENSG00000117533	VAMP4	-0.01	0.771256

ENSG00000163743	RCHY1	-0.02	0.773458
ENSG00000174891	RSRC1	-0.01	0.773697
ENSG00000170502	NUDT9	-0.02	0.774843
ENSG00000204619	PPP1R11	-0.01	0.776748
ENSG00000102401	ARMCX3	-0.01	0.779586
ENSG00000153885	KCTD15	-0.02	0.779575
ENSG00000120256	LRP11	-0.02	0.780796
ENSG00000178150	ZNF114	0.07	0.782396
ENSG00000091490	SEL1L3	0.02	0.785945
ENSG00000138231	DBR1	0.01	0.787148
ENSG00000185220	PGBD2	0.01	0.787196
ENSG00000099246	RAB18	-0.01	0.790727
ENSG00000124659	TBCC	0.01	0.796491
ENSG00000136783	NIPSNAP3A	0.01	0.798014
ENSG00000136144	RCBTB1	-0.01	0.798331
ENSG00000183091	NEB	0.06	0.799234
ENSG00000169255	B3GALT3	-0.02	0.800577
ENSG00000126698	DNAJC8	0.01	0.80176
ENSG00000110321	EIF4G2	0.01	0.803068
ENSG00000161558	TMEM143	-0.01	0.805055
ENSG00000249673	C4orf10	-0.01	0.807951
ENSG00000173702	MUC13	-0.06	0.813431
ENSG00000235217	TSPYL3	0.03	0.815034
ENSG00000115310	RTN4	-0.01	0.816354
ENSG00000072609	CHFR	0.01	0.818678
ENSG00000176903	PNMA1	-0.01	0.821385
ENSG00000256667	KLRA1	0.03	0.824499
ENSG00000157869	RAB28	0.01	0.827294
ENSG00000134245	WNT2B	-0.04	0.827775
ENSG00000118922	KLF12	-0.01	0.828391
ENSG00000117595	IRF6	0.03	0.829335
ENSG00000107566	ERLIN1	-0.01	0.829764
ENSG00000095574	IKZF5	0.01	0.832414
ENSG00000104219	ZDHHC2	0.02	0.834024
ENSG00000170482	SLC23A1	0.03	0.837399
ENSG00000168538	C4orf41	0.01	0.837384
ENSG00000107819	SFXN3	-0.01	0.837978
ENSG00000155100	OTUD6B	-0.01	0.839855
ENSG00000136940	PDCL	0.01	0.840395
ENSG00000206053	HN1L	0.01	0.840964
ENSG00000109180	OCIAD1	-0.01	0.842292
ENSG00000197363	ZNF517	-0.01	0.845029
ENSG00000171953	ATPAF2	-0.01	0.846681
ENSG00000106537	TSPAN13	0.02	0.848012
ENSG00000172932	ANKRD13D	-0.01	0.848223
ENSG00000112096	SOD2	0.02	0.848837
ENSG00000165312	OTUD1	-0.01	0.850099
ENSG00000111530	CAND1	-0.01	0.857232
ENSG00000145979	TBC1D7	-0.01	0.860764
ENSG00000185986	SDHAP3	0.02	0.861361
ENSG00000131931	THAP1	-0.01	0.862304
ENSG00000177051	FBXO46	0.01	0.862793
ENSG00000148719	DNAJB12	0.01	0.863908
ENSG00000135966	TGFBRAP1	0.01	0.866817
ENSG00000160218	TMEM1	-0.01	0.868415
ENSG00000135045	C9orf40	-0.01	0.870224
ENSG00000140006	WDR89	-0.01	0.871415
ENSG00000185189	NRBP2	-0.02	0.872186
ENSG00000172331	BPGM	-0.01	0.872131
ENSG00000147119	CHST7	-0.01	0.872926
ENSG00000163785	RYK	-0.01	0.874463
ENSG00000121022	COPS5	-0.01	0.874934
ENSG00000150459	SAP18	0.01	0.877846
ENSG00000184675	FLJ39827	-0.01	0.879451
ENSG00000137486	ARRB1	-0.01	0.88567
ENSG00000166477	LEO1	0.00	0.893908
ENSG00000163082	SGPP2	0.01	0.895529
ENSG00000115896	PLCL1	0.01	0.896972
ENSG00000181191	PJA1	0.01	0.898818
ENSG00000197563	PIGN	0.01	0.901457
ENSG00000166275	C10orf32	-0.01	0.903365
ENSG00000072422	RHOBTB1	-0.01	0.903013
ENSG00000100916	BRMS1L	-0.01	0.905958
ENSG00000111335	OAS2	0.01	0.908884
ENSG00000205937	RNPS1	0.00	0.911049
ENSG00000111261	MANSC1	-0.01	0.911336
ENSG00000078177	N4BP2	-0.01	0.912297
ENSG00000185442	FAM174B	0.01	0.918099
ENSG00000143443	C1orf56	0.01	0.923557
ENSG00000127914	AKAP9	-0.01	0.924644
ENSG00000135537	LACE1	-0.01	0.924888
ENSG00000196407	THEM5	0.01	0.924802
ENSG00000272325	NUDT3	0.00	0.931265
ENSG00000083817	ZNF416	0.00	0.932456
ENSG00000108468	CBX1	0.00	0.934263
ENSG00000100897	WDR23	0.00	0.934645
ENSG00000171703	TCEA2	0.00	0.937098
ENSG00000164125	C4orf18	0.01	0.938105
ENSG00000151276	MAGI1	-0.01	0.945096
ENSG00000163132	MSX1	-0.01	0.944894
ENSG00000138138	ATAD1	0.00	0.944913
ENSG00000105369	CD79A	0.01	0.945818
ENSG00000189184	PCDH18	0.01	0.946309
ENSG00000054983	GALC	0.00	0.946296
ENSG00000164414	SLC35A1	0.00	0.946727
ENSG00000113575	PPP2CA	0.00	0.949487
ENSG00000101342	C20orf118	-0.01	0.950728
ENSG00000197121	PGAP1	0.00	0.951514
ENSG00000164251	F2RL1	-0.01	0.955927
ENSG00000188986	COBRA1	0.00	0.960416
ENSG00000065154	OAT	0.00	0.963179
ENSG00000158483	FAM86C	0.00	0.965679
ENSG00000121289	CCDC123	0.00	0.965297
ENSG00000165672	PRDX3	0.00	0.965925
ENSG00000224531	LOC221710	0.00	0.966614
ENSG00000141338	ABCA8	-0.01	0.969103
ENSG00000161091	C19orf28	0.00	0.97109
ENSG00000092140	KIAA1333	0.00	0.973871
ENSG00000277586	NEFL	0.01	0.974537
ENSG00000182768	NGRN	0.00	0.9757
ENSG00000189007	ADAT2	0.00	0.979739
ENSG00000175895	PLEKHF2	0.00	0.979854
ENSG00000166997	CNPY4	0.00	0.981594
ENSG00000177706	FAM20C	0.00	0.983216
ENSG00000177889	UBE2N	0.00	0.986722
ENSG00000132670	PTPRA	0.00	0.989556
ENSG00000140543	DET1	0.00	0.99331
ENSG00000164683	HEY1	0.00	0.997782

Supplementary table9

60 gene signature of tumour infiltrating leukocytes

Symbol	Cell Type
BLK	B-cells
CD19	B-cells
MS4A1	B-cells
TNFRSF17	B-cells
FCRL2	B-cells
KIAA0125	B-cells
PNOC	B-cells
SPIB	B-cells
TCL1A	B-cells
PTPRC	CD45
CD8A	CD8 T cells
CD8B	CD8 T cells
CTSW	Cytotoxic cells
GNLY	Cytotoxic cells
GZMA	Cytotoxic cells
GZMB	Cytotoxic cells
GZMH	Cytotoxic cells
KLRB1	Cytotoxic cells
KLRD1	Cytotoxic cells
KLRK1	Cytotoxic cells
PRF1	Cytotoxic cells
NKG7	Cytotoxic cells
CCL13	DC
CD209	DC
HSD11B1	DC
CD244	Exhausted CD8
EOMES	Exhausted CD8
LAG3	Exhausted CD8
PTGER4	Exhausted CD8
CD163	Macrophages
CD68	Macrophages
CD84	Macrophages
MS4A4A	Macrophages
MS4A2	Mast cells
TPSAB1	Mast cells
CPA3	Mast cells
HDC	Mast cells
TPSB2	Mast cells
CSF3R	Neutrophils
S100A12	Neutrophils
CEACAM3	Neutrophils
FCAR	Neutrophils
FCGR3B	Neutrophils
FPR1	Neutrophils
SIGLEC5	Neutrophils
IL21R	NK CD56dim cells
KIR2DL3	NK CD56dim cells
KIR3DL1	NK CD56dim cells
KIR3DL2	NK CD56dim cells
NCR1	NK cells
XCL2	NK cells
XCL1	NK cells
CD3D	T-cells
CD3E	T-cells
CD3G	T-cells
CD6	T-cells
SH2D1A	T-cells
TRAT1	T-cells
TBX21	Th1 cells
FOXP3	Treg

Supplementary Table 10

Gene-Disease association of top genes differentially expressed in RCC tissues of men and women

Class	Term	number of genes	%	Fold Enrichment	Adjusted P Value
Renal	Hypertension	8	0.5	3.6	4.80E-03
	Chronic renal failure|Kidney Failure, Chronic	99	6	1.3	5.90E-03
	Gout	9	0.5	2.9	1.10E-02
Neurological	Brain structure	5	0.3	5.9	7.10E-03
	Alzheimer's disease	106	6.5	1.3	2.40E-03
	Attention Deficit Disorder with Hyperactivity	21	1.3	1.7	2.80E-02
Immune	Interleukin-6	6	0.4	3.7	1.80E-02
Cardiovascular	cardiovascular disease	11	0.7	2.2	2.50E-02
	Heart Failure	44	2.7	1.4	3.70E-02
Metabolic	Metabolic Syndrome X	12	0.7	2.3	1.40E-02
	Type 2 Diabetes| edema | rosiglitazone	229	14	1.2	3.20E-04
	HDL cholesterol	8	0.5	2.9	1.80E-02
	Hypertriglyceridemia	7	0.4	3.1	2.30E-02
	Obesity	42	2.6	1.3	4.40E-02
	Cholesterol, HDL	49	3	1.3	3.30E-02
Chemodependence	Tobacco Use Disorder	283	17.3	1.1	3.30E-02

Supplementary table 11

eQTLs in ccRCC tissue

SNP	Chromosome	Position	**Overlapped Gene**	**Type**	**Nearest Upstream Gene**	**Type**	**Nearest Downstream Gene**	**Type**	A1	A2	MAF	Regulatory proteins bound	Chromatin structure (DNAse sensitive)	chromatin state	tifs altered	vious eQTL	Probe	Gene	Gene_chromosome	Interaction P-value
rs79349965	1	248009263			OR11L1	protein_coding	TRIM58	protein_coding	G	C	0.05	CEBPB		PHMs (BLD)	Pax-4,Sox		ILMN_169	LYPD6B	2	9.48E-14
rs75843723	6	159956456			RP3-393E18.1	pseudogene	RP3-393E18.2	lincRNA	G	A	0.05			EHMks (IPSC, FAT, MU	26 altered motif		ILMN_168	CLDN10	13	3.02E-14
rs117206196	9	2929393	CARM1P1	pseudogene, non-coding intronic					G	T	0.06				Barx1		ILMN_228	ARPP-21	3	1.43E-13
rs79676858	20	39368367			RP4-644L1.2	lincRNA	RNA5SP484	rRNA	A	T	0.05		BLD	Mks (IPSC, FAT, MU	AhR		ILMN_170	TRIM50	7	1.20E-13
																	ILMN_180	FOXI2	10	1.33E-13
																	ILMN_171	FBN3	19	2.17E-13
																	ILMN_177	RHCG	15	2.44E-13
																	ILMN_174	PLA2G4F	15	3.19E-13
																	ILMN_177	PIB5PA	22	3.12E-14

Supplementary table 12

Genes showing variable differential expression in early versus late stage renal cell carcinoma according to sex

			Women		Men	
Symbol	probe_ID	Probe_Chr	Log Fold Change	Q Value	Log Fold Change	Q Value
THSD1	ILMN_173	chr13	-0.16	5.07E-08	-0.03	7.63E-02
KRT19	ILMN_173	chr17	1.21	1.29E-07	0.33	8.07E-02
ABHD12	ILMN_174	chr20	0.32	9.42E-07	0.10	6.13E-02
SNORA61	ILMN_324	chr1	0.38	2.08E-06	0.01	8.31E-01
C9orf91	ILMN_180	chr9	0.20	3.83E-06	0.03	3.14E-01
TRIB2	ILMN_171	chr2	-0.41	5.12E-06	-0.11	1.36E-01
CORO2A	ILMN_181	chr9	0.21	5.24E-06	0.03	7.53E-02
FERMT2	ILMN_169	chr14	-0.31	6.27E-06	0.02	7.83E-01
DNAJB13	ILMN_168	chr11	0.18	7.28E-06	0.01	8.20E-01
LPL	ILMN_178	chr8	-0.36	7.83E-06	-0.11	5.02E-02
SGK1	ILMN_322	chr6	-0.53	8.59E-06	-0.14	7.51E-02
CDK5RAP1	ILMN_171	chr20	0.13	9.93E-06	0.04	2.00E-01
PDCL	ILMN_169	chr9	-0.16	1.15E-05	-0.05	6.41E-02
AGPAT2	ILMN_173	chr9	0.22	1.20E-05	-0.02	5.43E-01
DUSP6	ILMN_167	chr12	-0.25	1.33E-05	-0.07	8.63E-02
PCDH12	ILMN_167	chr5	-0.36	1.42E-05	-0.07	2.03E-01
ITPR3	ILMN_181	chr6	0.45	1.70E-05	0.09	2.35E-01
FOSB	ILMN_175	chr19	-0.93	1.84E-05	-0.23	1.68E-01
FYN	ILMN_168	chr6	-0.33	2.12E-05	-0.06	1.21E-01
SERINC2	ILMN_211	chr1	0.41	2.14E-05	0.12	9.54E-02
FOS	ILMN_166	chr14	-0.78	2.24E-05	-0.13	3.62E-01
ZNF704	ILMN_165	chr8	-0.06	2.35E-05	-0.02	5.04E-02
FANCC	ILMN_168	chr9	-0.16	2.35E-05	-0.05	5.14E-02
GALE	ILMN_179	chr1	0.25	2.36E-05	0.07	1.81E-01
KIAA0494	ILMN_169	chr1	-0.24	2.59E-05	-0.06	1.07E-01
HKDC1	ILMN_175	chr10	0.47	2.96E-05	0.09	2.26E-01
APOL1	ILMN_176	chr22	0.21	3.09E-05	0.10	1.16E-01
CYGB	ILMN_175	chr17	-0.41	3.18E-05	-0.05	5.12E-01
PRKD1	ILMN_178	chr14	-0.24	3.47E-05	-0.03	4.15E-01
LOC64501	ILMN_176	chr4	0.15	3.71E-05	0.05	7.17E-02
SPAG9	ILMN_176	chr17	-0.15	3.92E-05	-0.04	1.35E-01
ZMYND8	ILMN_165	chr20	0.19	3.98E-05	0.02	1.42E-01
ZNF711	ILMN_180	chrX	-0.08	4.34E-05	-0.02	1.11E-01
SLC12A2	ILMN_172	chr5	-0.26	4.52E-05	-0.09	5.26E-02
DTD1	ILMN_177	chr20	0.18	4.65E-05	0.06	1.03E-01
MED17	ILMN_324	chr11	-0.08	4.75E-05	-0.02	1.42E-01
ARAP3	ILMN_324	chr5	-0.33	4.85E-05	-0.11	6.46E-02
RAP2A	ILMN_167	chr13	-0.26	5.17E-05	-0.08	1.47E-01
SGCE	ILMN_167	chr7	-0.46	5.58E-05	-0.13	1.72E-01
FRMD6	ILMN_176	chr14	-0.25	5.67E-05	-0.01	7.01E-01
EPS8L1	ILMN_167	chr19	0.18	6.12E-05	0.07	1.02E-01
C3orf54	ILMN_169	chr3	-0.28	6.31E-05	-0.04	4.08E-01
MYEOV	ILMN_219	chr11	0.32	7.37E-05	0.05	5.57E-01
GABPA	ILMN_175	chr21	-0.19	7.38E-05	-0.04	2.24E-01
ZBTB46	ILMN_171	chr20	-0.22	7.66E-05	-0.07	1.20E-01
SNORA16A	ILMN_324	chr1	0.08	8.58E-05	0.01	5.18E-01
PTGS1	ILMN_233	chr9	0.48	9.48E-05	0.08	5.31E-01
IL17RD	ILMN_172	chr3	-0.05	1.04E-04	-0.01	4.19E-01
CNTN4	ILMN_177	chr3	-0.08	1.05E-04	0.00	7.68E-01
C1QL4	ILMN_180	chr12	-0.46	1.07E-04	-0.11	1.32E-01
GTF3A	ILMN_165	chr13	0.22	1.08E-04	0.07	1.14E-01
MDH2	ILMN_207	chr7	0.18	1.13E-04	0.03	4.19E-01
TWF2	ILMN_170	chr3	0.17	1.16E-04	0.06	6.41E-02
SNX1	ILMN_236	chr15	-0.11	1.18E-04	-0.04	5.90E-02
ABCA8	ILMN_179	chr17	-0.24	1.28E-04	-0.04	3.14E-01
MTSS1	ILMN_167	chr8	-0.22	1.35E-04	-0.04	2.90E-01
THAP11	ILMN_178	chr16	-0.17	1.38E-04	-0.05	2.11E-01
MSX1	ILMN_177	chr4	-0.29	1.55E-04	-0.03	6.29E-01
TSPYL4	ILMN_174	chr6	-0.11	1.65E-04	-0.04	5.73E-02
CALM3	ILMN_166	chr19	-0.20	1.66E-04	-0.02	5.80E-01
ZEB1	ILMN_176	chr10	-0.09	1.72E-04	-0.02	1.45E-01
ARHGEF9	ILMN_177	chrX	-0.06	1.82E-04	-0.02	6.95E-02
CSDC2	ILMN_180	chr22	-0.34	1.83E-04	-0.05	3.72E-01
EFNB3	ILMN_169	chr17	-0.16	1.95E-04	-0.01	7.77E-01
FAM177A1	ILMN_176	chr14	-0.22	2.03E-04	-0.04	3.06E-01
RNF103	ILMN_169	chr2	-0.16	2.09E-04	-0.05	9.93E-02
MAML2	ILMN_176	chr11	-0.09	2.15E-04	-0.01	6.96E-01
CHST7	ILMN_173	chrX	-0.25	2.30E-04	-0.05	2.56E-01
LOC72817	ILMN_327	chr5	0.13	2.51E-04	0.03	2.77E-01
DNAJB4	ILMN_222	chr1	-0.13	2.55E-04	-0.04	6.75E-02
PRKAR1A	ILMN_173	chr17	-0.35	2.76E-04	-0.11	1.13E-01
TMC7	ILMN_168	chr16	-0.05	3.11E-04	-0.02	7.62E-02
CTSZ	ILMN_166	chr20	0.37	3.36E-04	0.10	2.11E-01
LOC10013	ILMN_319	chr9	0.12	3.74E-04	0.05	5.12E-02
CCDC123	ILMN_167	chr19	0.10	3.89E-04	0.05	5.06E-02
FAM169B	ILMN_167	chr15	0.14	3.89E-04	0.03	3.45E-01
ALPL	ILMN_170	chr1	-0.68	4.01E-04	-0.25	8.58E-02
SIAH1	ILMN_238	chr16	-0.13	4.04E-04	-0.07	5.10E-02
PCDH18	ILMN_179	chr4	-0.18	4.34E-04	-0.06	6.99E-02
ITGB4	ILMN_233	chr17	0.28	4.43E-04	-0.01	8.85E-02
CCNYL1	ILMN_216	chr2	-0.12	5.21E-04	-0.01	7.30E-01
ITPKB	ILMN_170	chr1	-0.19	5.22E-04	-0.07	8.88E-02
MCC	ILMN_179	chr5	-0.05	5.88E-04	-0.01	1.85E-01
NP	ILMN_217	chr14	-0.22	6.39E-04	-0.01	7.62E-01
FATE1	ILMN_180	chrX	-0.14	6.43E-04	0.00	9.59E-01
BTN2A1	ILMN_236	chr6	-0.17	6.54E-04	-0.01	1.47E-01
RASSF4	ILMN_169	chr10	0.26	6.60E-04	-0.01	8.50E-01
ADCK4	ILMN_170	chr19	0.14	6.72E-04	0.04	2.35E-01
HEY1	ILMN_178	chr8	-0.32	7.14E-04	-0.11	1.47E-01
C16orf93	ILMN_217	chr16	0.17	7.57E-04	0.08	6.12E-02
SVIL	ILMN_167	chr10	-0.26	7.68E-04	0.02	7.38E-01
SPON1	ILMN_179	chr11	-0.64	7.89E-04	-0.23	8.83E-02
LOC64907	ILMN_323	chrX	0.14	8.38E-04	0.05	1.24E-01
PNMA1	ILMN_177	chr14	-0.13	8.57E-04	-0.03	3.12E-01
MUM1L1	ILMN_212	chrX	0.16	8.68E-04	0.03	4.46E-01
C20orf75	ILMN_176	chr20	0.17	8.73E-04	0.06	2.51E-01
DUSP23	ILMN_165	chr1	0.27	9.10E-04	0.09	1.88E-01
SYT13	ILMN_165	chr11	0.62	9.27E-04	0.26	6.32E-02
FLJ33630	ILMN_324	chr5	-0.08	9.46E-04	-0.03	8.10E-02
GJC2	ILMN_172	chr1	-0.27	9.55E-04	-0.01	8.51E-01
CNPY4	ILMN_175	chr7	-0.07	1.01E-03	0.03	3.26E-01
KCNN2	ILMN_168	chr5	-0.08	1.08E-03	-0.03	1.43E-01
PRDX1	ILMN_236	chr1	0.13	1.17E-03	0.02	6.31E-01
CETP	ILMN_168	chr16	-0.10	1.17E-03	-0.07	1.37E-01
P2RY14	ILMN_234	chr3	-0.09	1.23E-03	-0.02	2.09E-01
TARS2	ILMN_177	chr1	0.10	1.24E-03	0.03	1.96E-01
PBX2	ILMN_168	chr6	-0.14	1.26E-03	0.02	5.19E-01
CTCF	ILMN_178	chr16	-0.11	1.28E-03	-0.05	6.78E-02
ZNF543	ILMN_171	chr19	-0.07	1.30E-03	-0.02	1.76E-01
FAM46C	ILMN_171	chr1	0.33	1.32E-03	0.16	6.59E-02
SFRS7	ILMN_177	chr2	-0.17	1.33E-03	-0.02	5.17E-01
GNAQ	ILMN_165	chr9	-0.17	1.48E-03	-0.06	1.55E-01
STEAP4	ILMN_177	chr7	-0.10	1.49E-03	-0.04	7.48E-02
RNF5	ILMN_204	chr6	-0.16	1.54E-03	-0.07	7.49E-02
ASB9	ILMN_237	chrX	-0.13	1.54E-03	-0.04	1.70E-01
LOC10013	ILMN_319	chr1	0.23	1.63E-03	0.11	5.09E-02
C10orf118	ILMN_173	chr10	-0.08	1.63E-03	-0.02	1.99E-01
FAM190B	ILMN_212	chr10	-0.16	1.71E-03	-0.05	1.83E-01
LOC65316	ILMN_320	chr9	0.14	1.73E-03	-0.02	6.80E-02
SUCNR1	ILMN_168	chr3	-0.20	1.73E-03	-0.04	2.58E-01
NGFR	ILMN_175	chr17	-0.10	1.79E-03	-0.03	2.15E-01
HIP1	ILMN_170	chr7	-0.13	1.82E-03	-0.02	4.70E-01
ANKRD33	ILMN_166	chr12	0.32	1.84E-03	0.11	1.37E-01
KCTD15	ILMN_178	chr19	-0.13	1.85E-03	0.02	4.94E-01
PLCE1	ILMN_178	chr10	0.20	1.88E-03	-0.04	4.59E-01
SENP7	ILMN_238	chr3	-0.15	1.89E-03	-0.06	8.56E-02
RAP1A	ILMN_180	chr1	-0.06	1.91E-03	-0.01	5.86E-01
NID1	ILMN_167	chr1	-0.10	1.92E-03	-0.01	6.42E-01
TTLL12	ILMN_166	chr22	0.14	1.93E-03	0.04	3.66E-01
LOC10013	ILMN_324	chr3	0.20	2.03E-03	0.08	1.01E-01
SLC35F3	ILMN_181	chr1	0.16	2.08E-03	-0.01	9.04E-01
DLX1	ILMN_167	chr2	-0.09	2.13E-03	-0.03	8.13E-02
LOC10013	ILMN_329	chr6	0.26	2.20E-03	0.10	1.22E-01
NBL1	ILMN_240	chr1	0.36	2.21E-03	0.08	4.18E-01
ZFP36	ILMN_172	chr19	-0.44	2.28E-03	-0.01	9.47E-01
B3GNT9	ILMN_178	chr16	-0.05	2.29E-03	-0.01	3.18E-01
RARS	ILMN_175	chr5	0.13	2.32E-03	0.01	7.00E-01
C1orf55	ILMN_174	chr1	-0.15	2.32E-03	-0.04	2.31E-01
JUNB	ILMN_208	chr19	-0.27	2.35E-03	-0.09	1.92E-01
SF1	ILMN_171	chr11	-0.07	2.37E-03	-0.02	3.14E-01
C4orf49	ILMN_207	chr4	-0.42	2.50E-03	-0.14	1.40E-01
PHLDA3	ILMN_165	chr1	0.22	2.57E-03	0.09	7.04E-02
WRNIP1	ILMN_233	chr6	-0.14	2.60E-03	-0.03	1.65E-01
KIAA0391	ILMN_170	chr14	-0.14	2.66E-03	-0.06	5.68E-02
WDR23	ILMN_176	chr14	-0.11	2.74E-03	-0.03	2.46E-01
DDX3X	ILMN_179	chrX	-0.17	2.77E-03	-0.03	5.17E-01

HIGD2A	ILMN_177	chr5	0.19	2.78E-03	0.02	7.33E-01
C1orf186	ILMN_169	chr1	0.35	2.78E-03	-0.04	6.92E-01
NCOA7	ILMN_168	chr6	-0.24	2.79E-03	-0.06	3.30E-01
RPS6KA1	ILMN_171	chr1	0.18	2.83E-03	0.06	1.93E-01
RAB11A	ILMN_171	chr15	-0.12	2.99E-03	-0.04	2.18E-01
MCOLN2	ILMN_166	chr1	0.23	3.01E-03	0.09	1.63E-01
MOBKL2C	ILMN_179	chr1	-0.12	3.21E-03	0.00	8.95E-01
KCNE3	ILMN_178	chr11	-0.06	3.23E-03	0.02	1.62E-01
TP53I11	ILMN_171	chr11	-0.12	3.24E-03	-0.05	1.45E-01
GNG2	ILMN_180	chr14	-0.16	3.33E-03	-0.02	6.66E-01
NR2C2	ILMN_325	chr3	-0.07	3.43E-03	-0.03	1.51E-01
SMARCA1	ILMN_237	chrX	-0.15	3.43E-03	-0.06	1.54E-01
ARRB1	ILMN_232	chr11	-0.12	3.64E-03	0.03	2.74E-01
UGT2B28	ILMN_178	chr4	0.09	3.72E-03	-0.03	4.02E-01
PDXK	ILMN_167	chr21	0.22	3.72E-03	0.02	7.88E-01
COL21A1	ILMN_179	chr6	-0.07	3.76E-03	-0.02	1.59E-01
PDZD8	ILMN_169	chr10	0.10	3.79E-03	0.05	6.58E-02
GM2A	ILMN_222	chr5	0.13	3.85E-03	0.06	1.08E-01
ERGIC1	ILMN_166	chr5	0.18	3.92E-03	0.07	1.67E-01
LOC34318	ILMN_327	chr1	0.11	3.93E-03	0.04	8.32E-02
ZHX1	ILMN_240	chr8	-0.09	4.14E-03	-0.04	1.10E-01
DCLK2	ILMN_171	chr4	-0.10	4.33E-03	-0.04	9.54E-02
LOC10012	ILMN_326	chr2	0.22	4.35E-03	0.08	1.69E-01
PLEKHA1	ILMN_166	chr10	-0.22	4.43E-03	-0.04	4.79E-01
SLC34A2	ILMN_218	chr4	0.17	4.66E-03	0.07	2.49E-01
ARPP-21	ILMN_228	chr3	-0.10	4.97E-03	0.02	3.60E-01
GGT2	ILMN_326	chr22	-0.14	5.22E-03	-0.02	5.95E-01
LOC10012	ILMN_317	chr17	0.06	5.25E-03	0.02	2.75E-01
RAB18	ILMN_169	chr10	-0.11	5.37E-03	-0.02	4.36E-01
WFDC2	ILMN_330	chr20	0.49	5.38E-03	0.01	7.58E-01
TMEM233	ILMN_324	chr12	-0.07	5.44E-03	-0.03	1.00E-01
B3GALT5	ILMN_180	chr21	0.03	5.46E-03	0.02	5.56E-02
LAMC3	ILMN_168	chr9	-0.30	5.56E-03	-0.03	7.28E-01
TTC9	ILMN_324	chr14	-0.04	5.59E-03	-0.02	6.42E-02
LOC72967	ILMN_323	chr19	0.22	5.59E-03	0.08	1.59E-01
LRRN4	ILMN_324	chr20	0.06	5.78E-03	0.03	1.48E-01
SUV420H1	ILMN_169	chr11	-0.06	5.86E-03	-0.02	6.95E-02
OPCML	ILMN_174	chr11	-0.23	5.87E-03	-0.03	6.13E-01
RAPH1	ILMN_178	chr2	-0.16	5.89E-03	-0.03	6.73E-02
DAD1	ILMN_173	chr14	-0.20	5.99E-03	-0.07	2.38E-01
SRD5A1	ILMN_179	chr5	0.09	6.07E-03	-0.01	7.16E-01
PPP1R11	ILMN_174	chr6	-0.11	6.28E-03	-0.04	2.76E-01
LOC72803	ILMN_328	chr13	0.17	6.35E-03	0.03	5.62E-01
LOC55288	ILMN_323	chr12	-0.12	6.39E-03	-0.02	5.55E-01
LOC64629	ILMN_329	chr6	0.16	6.61E-03	0.04	3.88E-01
CRY1	ILMN_176	chr12	-0.13	6.81E-03	-0.05	9.54E-02
LOC64182	ILMN_166	chr7	0.09	6.88E-03	-0.03	1.98E-01
ZNF22	ILMN_179	chr10	-0.15	6.93E-03	-0.06	1.62E-01
LOC73002	ILMN_322	chr14	0.16	7.07E-03	0.05	2.02E-01
LOC64517	ILMN_321	chr17	0.21	7.09E-03	0.05	3.75E-01
HN1L	ILMN_170	chr16	0.14	7.32E-03	0.02	6.77E-01
LRIG1	ILMN_212	chr3	-0.19	7.36E-03	-0.02	7.36E-01
PLEKHH3	ILMN_180	chr17	-0.14	7.50E-03	-0.04	3.60E-01
SKAP1	ILMN_175	chr17	0.26	7.51E-03	0.06	3.94E-01
MRPS33	ILMN_174	chr7	0.18	7.65E-03	-0.03	5.69E-01
NEFL	ILMN_165	chr8	0.27	8.18E-03	0.13	1.01E-01
TM9SF1	ILMN_173	chr14	-0.12	8.36E-03	-0.04	2.46E-01
MUL1	ILMN_323	chr1	-0.11	8.42E-03	-0.06	6.18E-02
SFRS3	ILMN_172	chr6	-0.14	8.62E-03	-0.06	1.23E-01
VCL	ILMN_241	chr10	-0.12	8.73E-03	-0.03	4.19E-01
ARNTL	ILMN_240	chr11	-0.12	8.82E-03	-0.03	2.91E-01
SLC35A1	ILMN_174	chr6	-0.14	8.88E-03	-0.03	5.19E-01
C12orf10	ILMN_177	chr12	0.12	9.06E-03	0.08	5.70E-02
CSMD2	ILMN_177	chr1	-0.07	9.15E-03	0.00	9.83E-01
RGAG4	ILMN_175	chrX	-0.06	9.32E-03	-0.02	1.18E-01
PNOC	ILMN_167	chr8	0.08	9.32E-03	0.04	1.71E-01
TUBA3E	ILMN_165	chr2	0.23	9.88E-03	0.16	1.15E-01
GUSB	ILMN_166	chr7	0.12	9.91E-03	0.03	3.35E-01
DYRK3	ILMN_240	chr1	-0.08	9.97E-03	-0.03	1.76E-01
RAB42	ILMN_219	chr1	0.06	1.00E-02	0.01	7.02E-01
MLLT10	ILMN_174	chr10	-0.10	1.03E-02	-0.03	2.19E-01
ANKZF1	ILMN_167	chr2	0.12	1.04E-02	0.06	1.47E-01
RGS7	ILMN_168	chr1	-0.20	1.07E-02	0.00	9.82E-01
TNFSF14	ILMN_236	chr19	0.23	2.07E-02	0.20	8.43E-08
LOC72776	ILMN_329	chrY	-0.01	7.68E-01	0.13	1.57E-05
TIMP1	ILMN_171	chrX	0.28	2.09E-02	0.36	1.86E-05
CCL20	ILMN_165	chr2	0.41	1.89E-02	0.65	2.66E-05
SHRM	ILMN_168	chr4	-0.01	8.82E-01	-0.28	3.29E-05
DNAJA4	ILMN_177	chr15	-0.05	4.82E-01	-0.29	4.45E-05
IL27RA	ILMN_168	chr19	0.08	1.55E-01	0.20	4.77E-05
S100A16	ILMN_172	chr1	0.10	1.30E-01	0.21	5.05E-05
N4BP2	ILMN_222	chr4	0.13	1.80E-01	0.09	5.12E-05
SHROOM3	ILMN_324	chr4	0.02	8.01E-01	-0.21	6.55E-05
SIRT5	ILMN_179	chr6	-0.07	3.77E-02	-0.18	7.13E-05
C21orf71	ILMN_324	chr21	-0.04	7.75E-02	-0.05	9.52E-05
C10orf47	ILMN_175	chr10	-0.07	3.61E-02	-0.10	1.52E-04
LOC72992	ILMN_322	chr7	-0.05	5.17E-02	-0.10	1.63E-04
EDN2	ILMN_168	chr1	-0.07	3.09E-01	-0.26	1.82E-04
PDE10A	ILMN_212	chr6	0.04	2.43E-02	0.05	2.13E-04
ZNF667	ILMN_180	chr19	-0.08	1.75E-02	-0.06	2.81E-04
ACTN1	ILMN_223	chr14	0.10	3.32E-01	0.27	3.27E-04
CIDEC	ILMN_177	chr3	0.03	1.44E-02	0.23	3.42E-04
PPP2R2D	ILMN_180	chr10	-0.05	1.51E-01	-0.09	3.57E-04
TRIM54	ILMN_241	chr2	0.29	2.16E-02	0.14	3.71E-04
LTBR	ILMN_166	chr12	0.10	1.52E-01	0.19	3.94E-04
STXBP6	ILMN_217	chr14	0.20	9.85E-02	0.31	4.22E-04
TSPYL3	ILMN_173	chr20	-0.11	1.23E-01	-0.18	4.43E-04
FGF14	ILMN_175	chr13	0.01	5.73E-01	-0.06	4.51E-04
LDHA	ILMN_325	chr11	0.10	3.09E-02	0.14	4.60E-04
TRAK1	ILMN_228	chr3	-0.13	1.54E-02	-0.13	5.60E-04
EMR1	ILMN_178	chr19	0.01	5.90E-01	0.09	5.75E-04
SLC44A4	ILMN_171	chr6	0.13	1.01E-01	-0.23	6.74E-04
GHITM	ILMN_172	chr10	-0.14	2.64E-02	-0.17	7.55E-04
PIP5K1B	ILMN_173	chr9	-0.03	6.14E-02	-0.04	7.67E-04
BTBD11	ILMN_170	chr12	0.24	1.41E-02	0.28	7.87E-04
MYO1G	ILMN_169	chr7	0.15	2.75E-02	0.17	7.92E-04
NUDT9	ILMN_168	chr4	-0.09	6.73E-02	-0.12	8.46E-04
DIRAS2	ILMN_215	chr9	-0.19	3.28E-02	-0.17	9.37E-04
BPGM	ILMN_235	chr7	-0.10	8.62E-02	-0.16	1.03E-03
CNDP2	ILMN_172	chr18	-0.09	2.63E-01	-0.20	1.03E-03
GYS1	ILMN_171	chr19	0.04	7.18E-02	0.07	1.18E-03
HSD11B2	ILMN_181	chr16	-0.33	2.18E-02	-0.34	1.20E-03
NNMT	ILMN_171	chr11	0.23	1.85E-01	0.38	1.24E-03
ZNF533	ILMN_165	chr2	-0.11	3.31E-01	-0.30	1.36E-03
IGLL3	ILMN_208	chr22	0.25	1.27E-02	0.31	1.40E-03
FMOD	ILMN_178	chr1	0.16	2.13E-02	0.20	1.44E-03
LLGL2	ILMN_241	chr17	-0.05	5.16E-02	-0.05	1.45E-03
ZNF684	ILMN_175	chr1	-0.10	1.21E-02	-0.10	1.74E-03
ASGR2	ILMN_234	chr17	0.03	3.55E-01	0.06	1.94E-03
PLEKHF2	ILMN_210	chr8	-0.09	1.24E-02	-0.08	2.17E-03
PNPLA4	ILMN_166	chrX	-0.09	2.03E-02	-0.07	2.20E-03
SP5	ILMN_177	chr2	0.06	1.82E-02	0.08	2.23E-03
HDHD2	ILMN_170	chr18	-0.05	3.02E-01	-0.12	2.24E-03
SP8	ILMN_230	chr7	0.01	4.54E-01	0.06	2.25E-03
LOC10012	ILMN_325	chr19	-0.04	1.60E-02	-0.03	2.30E-03
SLC13A1	ILMN_180	chr7	-0.28	1.61E-02	-0.24	2.32E-03
EPB41L5	ILMN_204	chr2	-0.05	1.84E-01	-0.09	2.32E-03
KIAA1107	ILMN_179	chr1	-0.05	3.07E-02	-0.06	2.55E-03
GSTM2	ILMN_171	chr1	-0.04	1.94E-01	-0.07	2.55E-03
CCDC64B	ILMN_323	chr16	0.07	1.41E-01	0.15	2.69E-03
ZNF407	ILMN_174	chr18	-0.03	4.30E-01	-0.08	2.84E-03
IL1R2	ILMN_177	chr2	0.31	1.74E-02	0.38	2.85E-03
KLHL7	ILMN_176	chr7	-0.11	2.93E-02	-0.11	2.96E-03
RTN4	ILMN_174	chr2	-0.18	1.15E-01	-0.25	3.01E-03
DIO3OS	ILMN_324	chr14	0.06	5.21E-02	0.12	3.08E-03
PGLS	ILMN_179	chr19	0.09	4.17E-02	0.11	3.16E-03
RUFY2	ILMN_224	chr10	0.02	2.43E-01	-0.04	3.19E-03
BCKDHB	ILMN_239	chr6	-0.04	1.25E-01	-0.11	3.27E-03
ANK1	ILMN_168	chr8	-0.04	1.38E-01	-0.08	3.33E-03
METTL7B	ILMN_206	chr12	0.09	3.05E-01	0.21	3.39E-03
TBX3	ILMN_171	chr12	-0.10	1.08E-02	-0.07	3.48E-03
GLRB	ILMN_166	chr4	0.02	7.14E-01	-0.13	3.61E-03
LOC10013	ILMN_323	chr1	-0.09	1.15E-01	-0.13	3.97E-03
MYO6	ILMN_172	chr6	-0.11	2.04E-02	-0.10	4.00E-03
ARC	ILMN_171	chr8	-0.03	9.31E-02	-0.03	4.24E-03
ATAD1	ILMN_165	chr10	-0.16	1.13E-02	-0.14	4.28E-03
HPS6	ILMN_171	chr10	-0.07	6.06E-02	-0.09	4.40E-03
LOC14578	ILMN_172	chr15	-0.05	1.11E-02	-0.05	4.58E-03
LOC10012	ILMN_325	chr19	0.24	2.30E-02	0.22	4.59E-03
LOC72998	ILMN_322	chr5	0.03	5.84E-02	0.03	4.61E-03
C1QTNF1	ILMN_169	chr17	0.04	1.82E-01	0.31	4.63E-03
IRF6	ILMN_207	chr1	-0.05	2.13E-01	-0.09	4.64E-03
NRBP2	ILMN_173	chr8	0.16	5.43E-02	0.20	4.67E-03
LOC14643	ILMN_180	chr16	0.08	9.68E-02	0.14	4.77E-03
WDSUB1	ILMN_173	chr2	-0.06	2.55E-01	-0.11	4.79E-03
MGAT1	ILMN_176	chr5	0.17	1.20E-02	0.14	4.96E-03
METTL4	ILMN_173	chr18	-0.07	2.38E-02	-0.06	5.02E-03
ALDH8A1	ILMN_168	chr6	-0.14	3.91E-02	-0.20	5.19E-03
FAM82A2	ILMN_172	chr15	-0.07	1.16E-01	-0.10	5.31E-03
MAD1L1	ILMN_235	chr7	0.09	3.88E-02	-0.07	5.40E-03
SNAI2	ILMN_165	chr8	-0.01	7.69E-01	0.18	5.55E-03
NPY6R	ILMN_206	chr5	-0.15	1.60E-02	-0.11	5.86E-03
C5orf46	ILMN_171	chr5	0.41	4.39E-02	0.50	5.95E-03
ARSD	ILMN_168	chrX	-0.02	5.87E-01	-0.12	5.98E-03

Supplementary table 13

Pathway enrichment of genes showing sex-specific effect with stage

	Adjusted P Value
Gastrin-CREB signalling pathway via PKC and MAPK_Homo sapiens_R-HSA-881907	0.0002471
VEGFR2 mediated cell proliferation_Homo sapiens_R-HSA-5218921	0.0009713
Signaling by EGFR_Homo sapiens_R-HSA-177929	0.001333
VEGFA-VEGFR2 Pathway_Homo sapiens_R-HSA-4420097	0.00127
Signaling by FGFR4_Homo sapiens_R-HSA-5654743	0.001821
Signaling by FGFR3_Homo sapiens_R-HSA-5654741	0.001875
Signaling by VEGF_Homo sapiens_R-HSA-194138	0.001619
Signaling by FGFR1_Homo sapiens_R-HSA-5654736	0.002045
GRB2 events in EGFR signaling_Homo sapiens_R-HSA-179812	0.001875
TNF receptor superfamily (TNFSF) members mediating non-canonical NF-kB pathway_Homo sapiens_R-H	0.00278
Glutathione conjugation_Homo sapiens_R-HSA-156590	0.01349
Metabolism_Homo sapiens_R-HSA-1430728	0.02973
Metabolism of carbohydrates_Homo sapiens_R-HSA-71387	0.04262

Supplementary table14

Association of sex-biased genes with 5 years OS of ccRCC

	IARC					TCGA					
Gene	HR	95%CI-low	95%CI-upp	P Value	Q Value	HR	95%CI-low	95%CI-upp	P Value	Q Value	Independent replication summary
ETV6	2.24	1.58	3.19	6.9E-06	1.2E-02	1.87	1.33	2.61	2.7E-04	1.4E-03	Replicated in TCGA
BAIAP2L1	1.61	1.29	2.02	3.4E-05	1.5E-02	1.38	1.20	1.60	1.0E-05	2.0E-04	Replicated in TCGA
M6PRBP1	2.13	1.49	3.05	3.5E-05	1.5E-02	1.02	0.79	1.30	9.0E-01	0.92	Not significant in TCGA
RARRES1	1.38	1.19	1.61	2.7E-05	1.5E-02	1.11	1.01	1.21	2.6E-02	4.7E-02	Replicated in TCGA
GOLSYN	1.47	1.22	1.77	4.8E-05	1.7E-02	1.02	0.94	1.11	6.7E-01	0.74	Not significant in TCGA
CDC14B	0.27	0.14	0.51	5.9E-05	1.7E-02	0.60	0.47	0.78	1.1E-04	8.6E-04	Replicated in TCGA
ANK1	0.11	0.04	0.33	8.1E-05	2.0E-02	0.90	0.82	1.00	4.0E-02	0.07	Borderline significant in TCGA
C11orf70	1.95	1.38	2.76	1.4E-04	2.0E-02	1.26	1.13	1.41	2.3E-05	2.3E-04	Replicated in TCGA
IL20RB	1.27	1.13	1.43	1.1E-04	2.0E-02	1.08	1.01	1.15	1.7E-02	3.2E-02	Replicated in TCGA
LRG1	1.74	1.31	2.31	1.4E-04	2.0E-02	0.99	0.91	1.09	8.7E-01	0.92	Not significant in TCGA
MUC13	1.86	1.35	2.55	1.3E-04	2.0E-02	1.00	0.91	1.09	9.2E-01	0.92	Not significant in TCGA
PPAP2A	0.29	0.16	0.55	1.1E-04	2.0E-02	0.65	0.55	0.77	3.3E-07	1.3E-05	Replicated in TCGA
LOC439949	2.32	1.49	3.61	1.9E-04	2.3E-02	NA	NA	NA	NA	NA	NA
POLD4	2.97	1.68	5.26	1.9E-04	2.3E-02	1.31	0.97	1.77	8.4E-02	0.12	Borderline significant in TCGA
NDRG4	1.74	1.30	2.34	2.4E-04	2.6E-02	1.28	1.09	1.51	2.7E-03	6.4E-03	Replicated in TCGA
RAPGEF2	0.44	0.28	0.68	2.4E-04	2.6E-02	0.64	0.50	0.82	3.2E-04	1.4E-03	Replicated in TCGA
CDCP1	1.55	1.22	1.96	2.7E-04	2.7E-02	1.17	1.06	1.29	1.3E-03	4.4E-03	Replicated in TCGA
CRY2	0.48	0.32	0.72	3.3E-04	2.7E-02	0.66	0.52	0.83	3.7E-04	1.5E-03	Replicated in TCGA
RNF128	1.62	1.24	2.10	3.3E-04	2.7E-02	0.90	0.81	1.00	5.1E-02	0.08	Borderline inverse association in TCGA
TMEM45B	1.84	1.32	2.56	3.1E-04	2.7E-02	0.93	0.83	1.05	2.7E-01	0.36	Not significant in TCGA
TSPAN7	0.62	0.48	0.81	3.0E-04	2.7E-02	0.80	0.71	0.90	3.2E-04	1.4E-03	Replicated in TCGA
ASGR2	4.00	1.87	8.56	3.6E-04	2.8E-02	1.06	0.92	1.23	4.4E-01	0.51	Not significant in TCGA
ID4	0.18	0.07	0.47	3.7E-04	2.8E-02	0.78	0.69	0.89	2.9E-04	1.4E-03	Replicated in TCGA
C17orf39	0.12	0.04	0.39	4.5E-04	2.9E-02	NA	NA	NA	NA	NA	NA
DNER	1.68	1.26	2.25	4.6E-04	2.9E-02	1.02	0.96	1.09	4.4E-01	0.51	Not significant in TCGA
FYN	0.39	0.23	0.66	4.4E-04	2.9E-02	0.73	0.58	0.92	6.5E-03	1.3E-02	Replicated in TCGA
UGP2	1.95	1.34	2.83	4.4E-04	2.9E-02	0.87	0.62	1.22	4.3E-01	0.51	Not significant in TCGA
C5orf62	1.60	1.22	2.09	5.8E-04	3.1E-02	NA	NA	NA	NA	NA	NA
LOC729768	1.87	1.31	2.67	5.7E-04	3.1E-02	NA	NA	NA	NA	NA	NA
PDZD8	3.26	1.67	6.39	5.5E-04	3.1E-02	0.67	0.51	0.86	1.9E-03	5.4E-03	Inverse association in TCGA
PREP	4.08	1.83	9.10	5.7E-04	3.1E-02	1.09	0.67	1.76	7.3E-01	0.79	Not significant in TCGA
TTC39C	2.19	1.40	3.41	5.5E-04	3.1E-02	1.37	1.11	1.69	3.3E-03	7.3E-03	Replicated in TCGA
ANKRD47	0.34	0.18	0.63	6.1E-04	3.1E-02	0.78	0.66	0.91	2.0E-03	5.4E-03	Replicated in TCGA
HKDC1	1.51	1.19	1.92	7.7E-04	3.9E-02	0.91	0.80	1.04	1.5E-01	0.22	Not significant in TCGA
APOB	1.30	1.12	1.52	8.4E-04	4.0E-02	0.98	0.93	1.04	5.7E-01	0.66	Not significant in TCGA
RBP7	0.67	0.53	0.85	8.4E-04	4.0E-02	0.81	0.71	0.92	1.4E-03	4.4E-03	Replicated in TCGA
ALG2	0.03	0.00	0.22	8.6E-04	4.0E-02	0.69	0.48	1.00	5.1E-02	0.08	Borderline significant in TCGA
FMOD	1.77	1.26	2.48	9.3E-04	4.2E-02	1.09	0.99	1.20	7.2E-02	0.11	Borderline significant in TCGA
PGLS	2.44	1.43	4.15	1.1E-03	4.7E-02	1.22	0.92	1.61	1.7E-01	0.23	Not significant in TCGA
ICAM2	0.61	0.45	0.82	1.2E-03	4.8E-02	0.74	0.61	0.90	2.5E-03	6.3E-03	Replicated in TCGA
SHE	0.21	0.08	0.54	1.2E-03	4.8E-02	0.70	0.59	0.82	1.5E-05	2.0E-04	Replicated in TCGA
SLC7A1	1.55	1.19	2.02	1.2E-03	4.8E-02	1.09	0.90	1.31	3.8E-01	0.49	Not significant in TCGA
TRIB3	1.33	1.12	1.57	1.2E-03	4.8E-02	1.24	1.09	1.42	1.4E-03	4.4E-03	Replicated in TCGA
CLEC3B	0.67	0.52	0.85	1.3E-03	5.0E-02	0.84	0.74	0.94	3.7E-03	7.7E-03	Replicated in TCGA

Supplementary table 15

Pathway enrichment of genes differentially expressed in men and women and associated with survival in KEGG and PANTHER

	adjusted P Value
Galactose metabolism	0.002
Vasopressin synthesis Homo sapiens	0.02
Glycolysis Homo sapiens	0.03

Supplementary_table16

Association of sex-interacting eQTLs with 5 years overall survival of ccRCC

eQTL	Allele	HazardRatio	95%CI	P Value
	COMBINED			
rs79349965	Reference	ref	-	-
	Variant	0.55	[0.17;1.72]	0.3
rs75843723	Reference	ref	-	-
	Variant	1.31	[0.61;2.83]	0.49
rs117206196	Reference	ref	-	-
	Variant	0.46	[0.19;1.12]	0.09
rs79676858	Reference	ref	-	-
	Variant	1.49	[0.85;2.61]	0.16
	FEMALE			
rs79349965	Reference	ref	-	-
	Variant	1.83	[0.25;13.38]	0.55
rs75843723	Reference	ref	-	-
	Variant	2.14	[0.51;8.89]	0.29
rs117206196	Reference	ref	-	-
	Variant	0.76	[0.23;2.43]	0.64
rs79676858	Reference	ref	-	-
	Variant	1.22	[0.44;3.39]	0.71
	MALE			
rs79349965	Reference	ref	-	-
	Variant	0.41	[0.10;1.69]	0.22
rs75843723	Reference	ref	-	-
	Variant	1.17	[0.47;2.94]	0.73
rs117206196	Reference	ref	-	-
	Variant	0.29	[0.07;1.19]	0.09
rs79676858	Reference	ref	-	-
	Variant	1.7	[0.86;3.34]	0.13
